# Design and Optimization of Full‐Stokes Hyperspectro‐Polarimetric Encoding Metasurfaces Based on Conditional Multi‐Task Deep Learning

**DOI:** 10.1002/advs.202523143

**Published:** 2026-02-17

**Authors:** Chenjie Gong, Haodong Shi, Qi Wang, Yingchao Li, Hongyu Sun, Jiayu Wang, Guanlin Li, Jian Zhang, Qiang Fu, Huilin Jiang

**Affiliations:** ^1^ Jilin Provincial Key Laboratory of Space Optoelectronics Technology Changchun University of Science and Technology Changchun Jilin China; ^2^ Jilin Engineering Research Center of Photoelectric Measurement & Control Instruments Changchun Jilin China; ^3^ School of Optoelectronic Engineering Changchun University of Science and Technology Changchun Jilin China

**Keywords:** deep learning, inverse design, metasurface, spectro‐polarimetric encoding

## Abstract

Metasurfaces, as two‐dimensional metamaterials, offer an unprecedented avenue for multidimensional optical detection. However, existing full‐Stokes hyperspectro‐polarimetric encoding metasurface (FHPEM) designs rely on iterative trial‐and‐error processes and single‐structure optimization, limiting design efficiency and broadband precision. Here, an end‐to‐end conditional multitask learning framework is proposed that integrates physical encoding constraints into the metasurface design process. By explicitly incorporating correlation constraints as physical conditions during network training, this framework establishes an on‐demand metasurface design paradigm that directly generates structures with controlled encoding independence. Joint multitask learning further enables unified forward and inverse modeling across multiple metasurface configurations. Furthermore, a metasurface array screening strategy is developed by integrating forward prediction and correlation‐conditioned inverse design networks. Using this strategy, metasurface arrays with sizes of 4 × 4, 6 × 6, 8 × 8, and 10 × 10 are designed, achieving average Relevances reductions of 19.7%, 34.6%, 26.9%, and 36.5%, respectively, compared with arrays obtained through manual selection or machine‐learning‐based methods. A 4 × 4 metasurface array is fabricated and experimentally validated, demonstrating full‐Stokes spectro‐polarimetric reconstruction over 400–900 nm with a spectral resolution of 4 nm. These results demonstrate the potential of the proposed approach for high‐performance and highly integrated multidimensional optical systems.

## Graphical abstract

A conditional multi‐task deep learning framework is developed for designing and optimizing Full‐Stokes Hyperspectro‐Polarimetric Encoding Metasurfaces (FHPEMs). This framework achieves joint spectro‐polarimetric learning and unified forward–inverse design. Two novel FHPEM structures and an array screening strategy are developed, enabling automatic generation of arrays at different scales with low inter‐correlation and reconstruction errors.

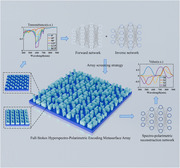

## 1 Introduction

Spectro‐polarimetric technology plays a crucial role in target detection. By integrating both the spectral [1, 2, 3, 4] and polarization information [5, 6] of a target, it substantially enhances information dimensionality [7, 8, 9, 10] and provides distinct advantages for target recognition [11, 12] and detection performance [13]. Owing to these merits, this technology has found wide applications in remote sensing [14], biology [15], medical diagnostics [16], and agricultural monitoring [17]. However, most existing spectro‐polarimetric imaging systems follow traditional designs that rely heavily on spectral elements, such as gratings [18], interferometers [19], and filters [20], together with polarization elements [21]. Although these systems enable the simultaneous acquisition of spectral and polarization information, achieving high spectral resolution and full‐Stokes information is challenging. Complex spectrometers, multi‐channel optical paths, repeated measurements and calibrations are required to maintain accuracy. Consequently, such systems are often bulky, structurally complicated, inefficient, and difficult to maintain high measurement accuracy.

The emergence of metasurface technology has opened new possibilities for innovative spectro‐polarimetric systems designs. Metasurfaces are artificially engineered planar structures composed of subwavelength meta‐atoms [22, 23] that can precisely manipulate the phase, amplitude, and polarization of electromagnetic waves [24, 25, 26, 27]. By carefully designing and arranging nanoscale structures, spectral and polarization information can be directly encoded into the optical field. The encoded information can then be decoded to reconstruct the complete spectro‐polarimetric data [28, 29]. This approach significantly reduces system size and simplifies the optical path, while enhancing imaging efficiency and real‐time measurement capability. However, conventional metasurface design methods rely heavily on the prior knowledge of researchers and iterative trial‐and‐error methods using full‐wave simulations [30]. When spectral and polarization information are encoded simultaneously, nanostructure parameters need to be repeatedly optimized for different wavelengths and polarization states. This process dramatically increases computational cost and reduces optimization efficiency, making rapidly identification of optimal structures under multiple objectives and constraints difficult. Consequently, the development of metasurfaces for high spectral resolution and high‐precision full‐Stokes detection remains limited.

In recent years, deep learning (DL) has shown great potential in metasurface design, providing an efficient new design paradigm [31, 32]. Through end‐to‐end training, DL significantly reduces manual intervention and can automatically generate desired predictions in both forward and inverse design processes [33, 34]. This capability accelerates optimization in complex nonlinear optical response tasks. Various generative models, including generative adversarial networks (GANs) [35, 36], variational autoencoders (VAEs) [37, 38], diffusion models (DMs) [39, 40], and Transformer architectures [41], have been applied to metasurface design. These models enable rapid construction and optimization of structures with diverse functionalities. However, in practice, these models often suffer from unbalanced subnetwork performance and an excessive number of hyperparameters. Such issues substantially increase computational complexity, slow training convergence, and limit model stability and usability [42, 43]. Moreover, existing DL‐based studies also focus on specific structure or single optical properties, lacking the ability to jointly optimize spectral and polarization responses and to generalize across different metasurface types. Additionally, no studies have reported the systematic integration of deep learning for both forward prediction and inverse optimization of full‐Stokes hyperspectro‐polarimetric encoding metasurfaces (FHPEMs).

In FHPEM systems, the correlation among different metasurface units directly determines the encoding capability for spectro‐polarimetric information as well as the reconstruction accuracy [28, 29, 44]. Consequently, the systematic generation of encoding metasurface units with low mutual correlation remains a central challenge. Most existing studies address this problem through manual selection from large structure libraries [28, 29] or by employing genetic‐algorithm‐based screening strategies [45]. Manual selection is strongly dependent on prior experience and make it difficult to guarantee global optimality in high‐dimensional parameter spaces. In contrast, genetic algorithms are typically limited to discrete structural representations and require repeated random generation and evaluation of candidate structures, resulting in rapidly increasing computational costs as the number of encoding channels and the array size grow. Moreover, these discrete and search‐based optimization methods hinder continuous control over correlation levels and limit efficient transferability across different system configurations. As the encoding dimensionality increases, such experience‐driven or discrete‐search approaches encounter pronounced limitations in both efficiency and scalability. Therefore, a system‐level metasurface array construction strategy that enables efficient, controllable, and scalable generation of low‐correlation spectro‐polarimetric encoding metasurfaces, while remaining applicable to multiple metasurface structure types, is highly desirable.

In this work, a conditional multitask deep learning framework with embedded physical encoding constraints is proposed for on‐demand metasurface design in spectro‐polarimetric systems. Unlike conventional encoding‐decoding schemes or isolated forward and inverse design strategies, the proposed model explicitly incorporates encoding‐related physical constraints into the learning process. As a result, system‐level design objectives can be directly satisfied during metasurface generation. By jointly learning spectral and polarization responses within a shared feature space, the framework establishes a unified forward‐inverse modeling paradigm. Under specified correlation constraints, multiple types of metasurface structures can be simultaneously predicted and generated. Based on this framework, two novel FHPEMs are designed, enabling coordinated control of spectro‐polarimetric information. Furthermore, a metasurface array generation and screening strategy is developed by integrating the trained forward prediction and correlation‐conditioned inverse design networks. This strategy allows FHPEM arrays that satisfy low‐correlation requirements to be constructed directly at the design stage. Arrays of different scales, including 4 × 4, 6 × 6, 8 × 8, and 10 × 10, are successfully generated, achieving Relevance metrics lower than those of the best‐performing existing metasurface arrays. As a proof of concept, a 4 × 4 metasurface array is fabricated and experimentally validated. The results demonstrate accurate reconstruction over a broad spectral range from 400 to 900 nm, together with high spectral resolution, reconstruction accuracy and stability. These results validate the efficiency and feasibility of the proposed FHPEM structures and design framework. This work establishes a scalable and physics‐aware paradigm for system‐level metasurface design, providing a solid foundation for next‐generation multidimensional optical detection and imaging systems.

## 2 Results

### 2.1 Mathematical Model of FHPEM

Our FHPEM comprises hundreds of spectro‐polarimetric superpixels, each consisting of arrays of judiciously designed meta‐atoms. A theoretical analysis of the spectro‐polarimetric information encoding process is first conducted based on a mathematical model. Specifically, as incident light passes through the metasurface, spectral and polarization information are modulated and encoded into the intensity of the transmitted light. This intensity encapsulates both the spectral energy distribution across different wavelengths and the corresponding polarization state information. To more precisely characterize this modulation and encoding process, the Stokes parameters *S* = [*S*_0_,*S*_1_,*S*_2_,*S*_3_] and the Mueller matrix are used to model the full‐Stokes spectro‐polarimetric behavior. Consequently, the intensity *I_i_* after passing through the *i*‐th meta‐atom can be expressed as follows

(1) $$I_{i}=\sum_{j=0}^{3}\int_{\lambda_{1}}^{\lambda_{m}}M_{0j}^{i}\left(\lambda \right)\cdot S_{j}\left(\lambda \right)d\lambda$$

where *m* denotes the number of spectral channels, and *M*_0*j*_(λ) represents the element in the first row and *j*‐th column of the Mueller matrix at wavelength λ. Accordingly, the spectro‐polarimetric transmission equation composed of *n* meta‐atoms can be expressed as follows

(2) $$\begin{array}{c} I=M_{0}\cdot S+e\\ I={\left(I_{1},I_{2},\text{…},I_{n}\right)}^{T}\\ e={\left(e_{1},e_{2},\text{…},e_{n}\right)}^{T}\\ S=\left(S_{0}\left(\lambda_{1}\right),S_{1}\left(\lambda_{1}\right),S_{2}\left(\lambda_{1}\right),S_{3}\left(\lambda_{1}\right),\text{…},S_{0}\left(\lambda_{m}\right),S_{1}\left(\lambda_{m}\right),\right.\\ \;{\left.S_{2}\left(\lambda_{m}\right),S_{3}\left(\lambda_{m}\right)\right)}^{T}\\ M_{0}=\left(\begin{array}{c} M_{00}^{1}\left(\lambda_{1}\right)M_{01}^{1}\left(\lambda_{1}\right)M_{02}^{1}\left(\lambda_{1}\right)M_{03}^{1}\left(\lambda_{1}\right)\text{…}M_{00}^{1}\left(\lambda_{m}\right)\\ M_{01}^{1}\left(\lambda_{m}\right)M_{02}^{1}\left(\lambda_{m}\right)M_{03}^{1}\left(\lambda_{m}\right)\\ \text{…}\text{…}\text{…}\text{…}\\ \text{…}\text{…}\text{…}\text{…}\text{…}\\ M_{00}^{n}\left(\lambda_{1}\right)M_{01}^{n}\left(\lambda_{1}\right)M_{02}^{n}\left(\lambda_{1}\right)M_{03}^{n}\left(\lambda_{1}\right)\text{…}M_{00}^{n}\left(\lambda_{m}\right)\\ M_{01}^{n}\left(\lambda_{m}\right)M_{02}^{n}\left(\lambda_{m}\right)M_{03}^{n}\left(\lambda_{m}\right) \end{array}\right) \end{array}$$

where ***e*** denotes the detection errors of each sensor unit, and **M**_0_ represents the spectro‐polarimetric encoding matrix (see Section S1 for details).

According to Equation (2), the spectral and polarization information can be recovered by directly pseudo‐inverting the spectro‐polarimetric encoding matrix **M**_0_. To ensure the accuracy and stability of the reconstruction process, it is essential to design *n* highly anisotropic meta‐atoms with significantly distinct spectral and polarization responses. This minimized the correlation coefficients between different rows of **M**_0_. The correlation coefficient *C*_*i*,*j*_ between the *i*‐th and *j*‐th meta‐atoms is defined as follows

(3) $$C_{i,j}=\frac{cov\left(M_{0}^{i},M_{0}^{j}\right)}{\sigma_{M_{0}^{i}}\sigma_{M_{0}^{j}}}$$

where $M_{0}^{i}$ and $M_{0}^{j}$ denote the *i*‐th and *j*‐th rows of **M**_0_, respectively; cov and σ represent the covariance and standard deviation, respectively. A larger value of *C*_*i*,*j*_ indicates higher correlation and lower distinctiveness between the meta‐atoms, which reduces accuracy of spectro‐polarimetric reconstruction. Conversely, a smaller *C*_*i*,*j*_ implies lower correlation and greater distinctiveness, thereby enhancing the reconstruction accuracy of the spectro‐polarimetric information.

**FIGURE 1:**
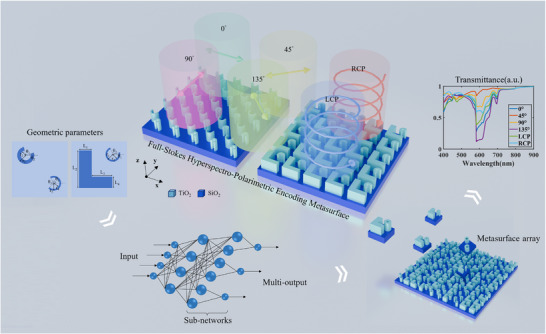
Schematic of the FHPEM. The metasurface is designed with a lattice period of 500 nm and a nanopillar height of 600 nm, covering a spectral range of 400–900 nm.

To realize metasurfaces with distinct spectro‐polarimetric responses and minimal correlation, two different FHPEM structures are designed, as shown in Figure 1. Titanium dioxide (TiO_2_) is chosen as the nanopillar material due to its high refractive index, low optical loss, and excellent electromagnetic performance in the visible and near‐infrared ranges. Additionally, TiO_2_ is highly compatible with conventional lithography and deposition techniques [46]. The nanopillars are fabricated on a SiO_2_ substrate, ensuring both structural and optical stability, while also facilitating subsequent fabrication and integration.

In terms of structural configuration design, both metasurface configurations adopt diatomic unit cells composed of two nanostructures with different geometries, which increases the number of supported modes and lifts modal degeneracies. In the first configuration, two split‐ring resonators are arranged diagonally within the unit cell, positioned at the upper‐left and lower‐right corners. This layout breaks both the centrosymmetry and mirror symmetry while maintaining a compact footprint. As a result, multiple anisotropic resonant modes that are highly sensitive to the incident polarization state can be excited. The relative orientation and spatial separation between the two split‐ring resonators further enhance near‐field coupling effects, resulting in pronounced differences in intensity distributions under different polarization conditions. The second configuration introduces a combination of a split‐ring resonator and an L‐shaped nanostructure, with the split‐ring located at the upper‐right corner and the L‐shaped structure at the lower‐left corner. This design simultaneously introduces asymmetry in geometry, orientation, and spatial arrangement, which leads to a more diverse set of supported resonant modes. Compared with the dual split‐ring configuration, this structure exhibits stronger polarization selectivity and more complex modal coupling behavior. Consequently, it shows response characteristics that are distinctly different from those of the first configuration in both the spectral and polarization domains.

From a physical perspective, the spectro‐polarimetric diversity of the two metasurface configurations mainly originates from the scattering, interference, and coupling of Bloch modes in the periodic metasurface structures [47, 48]. The geometric asymmetry of the nanostructures breaks the original modal degeneracy, allowing different Bloch mode branches to be selectively excited under different incident polarization states. As a result, the resonance positions and bandwidths become highly sensitive to the structural layout. By employing two metasurface configurations with distinct symmetry‐breaking mechanisms, complementary and weakly correlated spectro‐polarimetric responses can be achieved without significantly increasing fabrication complexity. This characteristic is particularly important for enhancing feature independence in subsequent computational imaging or information decoding processes.

Numerical simulations of the metasurface structures were performed using the finite‐difference time‐domain (FDTD) method. For each design, the geometric parameters were systematically varied and analyzed under incident light with linear polarization angles of 0°, 45°, 90°, and 135°, as well as under left‐ and right‐handed circular polarizations (LCP and RCP). The lattice period and nanopillar height were fixed at 500 and 600 nm, respectively, across a spectral range of 400–900 nm, sampled at 501 points. To suppress near‐field coupling effects, adjacent meta‐atoms were separated by at least 100 nm [49, 50]. In total, 2900 spectro‐polarimetric datasets were generated for each structure (see Section S2 for details).

In addition, to further elucidate the physical mechanisms of the two metasurface structures under different polarization conditions, we numerically simulated their electric‐field distributions. The corresponding results are presented in Section S3. The simulation results show that, for the same structure, the electromagnetic modes excited under different polarization states exhibit pronounced differences in both spatial distribution and local field intensity. Meanwhile, under identical polarization illumination, the two structures display distinctly different local electric‐field distributions. These differences are clearly observed in both the transverse plane of the nanostructures (x‐y plane) and the longitudinal plane along the propagation direction (x‐z plane). This indicates that structural asymmetry plays a crucial role in determining polarization selectivity and modal coupling behavior.

### 2.2 The Conditional Multitask Network Architecture

**FIGURE 2:**
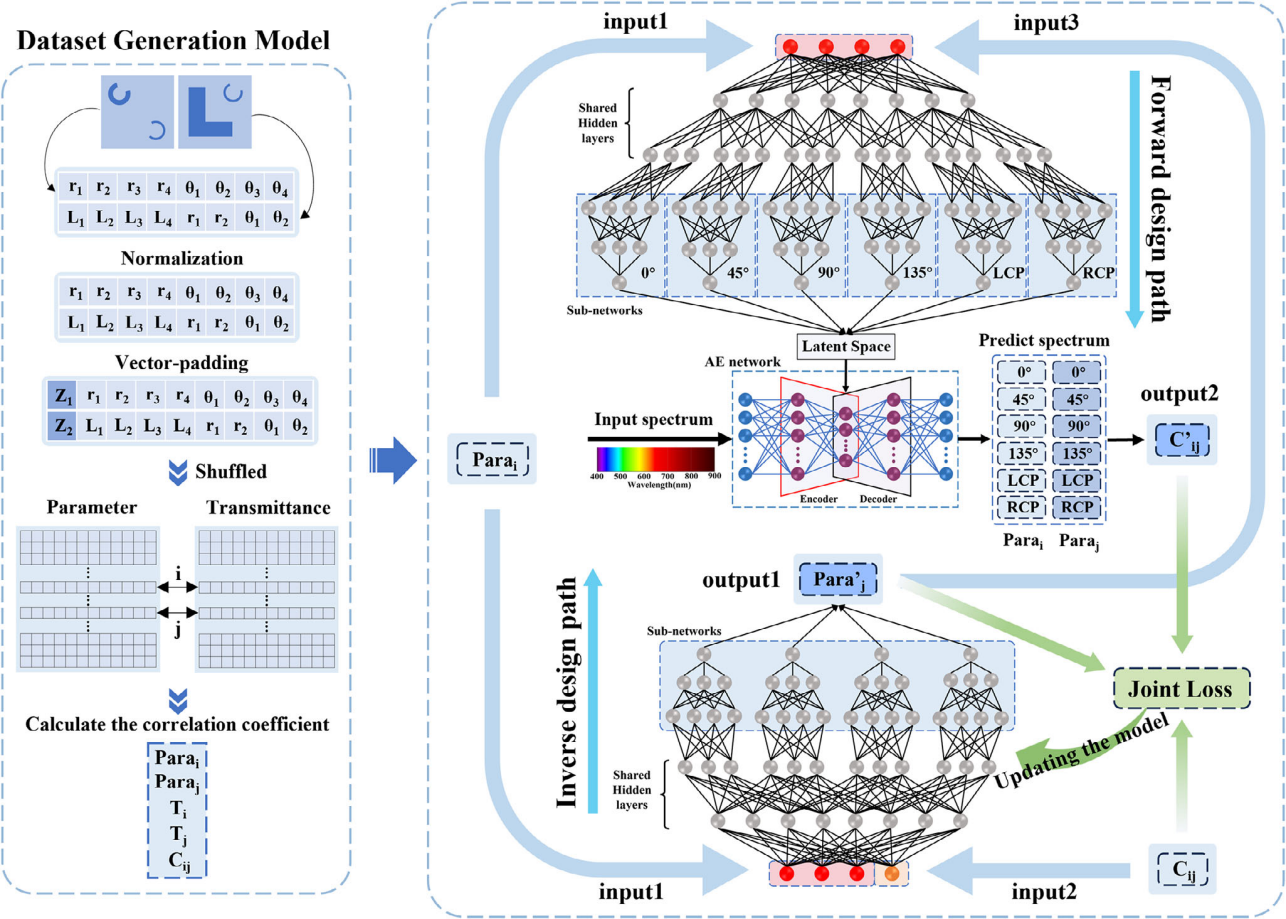
Deep learning framework for FHPEM design. The model consists of three components: a dataset generation module, a forward design network, and an inverse design network. Within the network, Para_i_ is provided as input1 to both networks, while C_ij_ serves as input2 to the inverse network. The inverse network outputs Para′_j_ (a combination of four parameters), which is then used as input3 to the forward network. The forward network subsequently predicts C′_ij_ (a combination of four correlation coefficients) as output2. Finally, the losses for Para′_j_ and C_ij_ are computed and back‐propagated through the inverse network for iterative updating.

Figure 2 illustrates the deep learning framework developed for the FHPEM. The model consists of three main components: a dataset generation module, a forward design network, and an inverse design network, which together form an efficient data‐driven optimization framework. The dataset generation module constructs the database required for training. By incorporating conditional information (latent vectors **Z**) into the geometric parameters, it not only enhances the diversity and coverage of the data but also enables the network to effectively distinguish between different types of metasurface structures. Both the forward and inverse design networks employ a multitask learning strategy and operate in tandem. This configuration improves network adaptability and generalization while addressing the one‐to‐many mapping problem in structural parameter design. The forward design network takes metasurface structures as input and predicts the spectro‐polarimetric response, enabling efficient simulation of complex optical properties. The inverse network generates new metasurface structures based on given structures and correlation coefficients. This approach overcomes the high computational cost and low search efficiency of traditional inverse design methods. By integrating conditional multitask learning with a joint optimization strategy, the model can directly predict paired structures with target correlation coefficients. This significantly improves the design efficiency of the FHPEM.

#### 2.2.1 Forward Design Network

In the forward design network, the input data were normalized to improve training performance and convergence speed. Data normalization scales input features of different magnitudes to a common range, mitigating the risk of gradient vanishing or explosion caused by large discrepancies in feature scales. This process ensures stable gradients during backpropagation and allows model parameters to be updated efficiently and smoothly [51].

Because the two proposed metasurface structures differ significantly in their geometric parameters, directly training a single neural network on both datasets may prevent the network from effectively capturing the distinct features of each structure, thereby limiting its learning capacity and prediction accuracy. To improve the model's ability to discriminate between different metasurface data and accurately predict the corresponding responses, latent vectors **Z** were introduced into the input data (e.g., sampled from normal distributions with different means and standard deviations). After optimization, the latent vector dimension was set to 4 (see Section S4 for optimization details). Incorporating latent vectors into the geometric parameters enhances the model's robustness to local feature variations. This enables the neural network to more effectively differentiate the characteristics of the two metasurface structures.

The conditional multitask forward design network developed in this work comprises a shared layer and six parallel subnetworks. Each subnetwork is dedicated to processing a specific polarization response. This structure enhances both prediction accuracy and generalization. Before training, the 5800 data samples are randomly shuffled to improve the network's adaptability to diverse structural combinations and to enhance robustness. Following dimensional transformation and feature extraction, the input data are partitioned into six sub‐vectors. These sub‐vectors are fed into structurally identical but independent subnetworks for deep feature extraction and reinforcement. In this network, the 1 × 12 input geometric parameters must be mapped to 501 × 6 outputs. This large dimensional mismatch increases network instability, particularly in spectral regions with large fluctuations, potentially hindering convergence [52]. To address this issue, an autoencoder is incorporated into the forward network. The autoencoder expands each subnetwork's 200‐dimensional output to 501 dimensions, generating 501‐dimensional data for six polarization directions (see Section S5 for details of the autoencoder). The loss function Forward_Loss of the forward network is defined as follows

(4) $$\mathrm{Forward}\_\mathrm{Loss}=\frac{1}{k_{1}}\cdot \sum_{i=1}^{k_{1}}\alpha_{i}\cdot {\left(y_{i}^{T}-F_{i}^{T}\left(x^{p},w_{1},b_{1}\right)\right)}^{2}$$

where, *k*_1_ denotes the number of tasks (corresponding to polarization directions of 0°, 45°, 90°, 135°, LCP, and RCP); *y^T^* represents the simulated transmittance; and *F^T^* is the forward network function, which takes geometric parameters *x^p^*, weights *w*_1_, and biases *b*_1_ as inputs to predict the output responses. α is the weighting factor for each task. To ensure the model learns features across all polarization directions in a balanced manner, α is set to 1 for all tasks. The Adam moment estimation stochastic optimization approach is employed to compute an adaptive learning rate for each of the internal parameters of the model [53].

**FIGURE 3:**
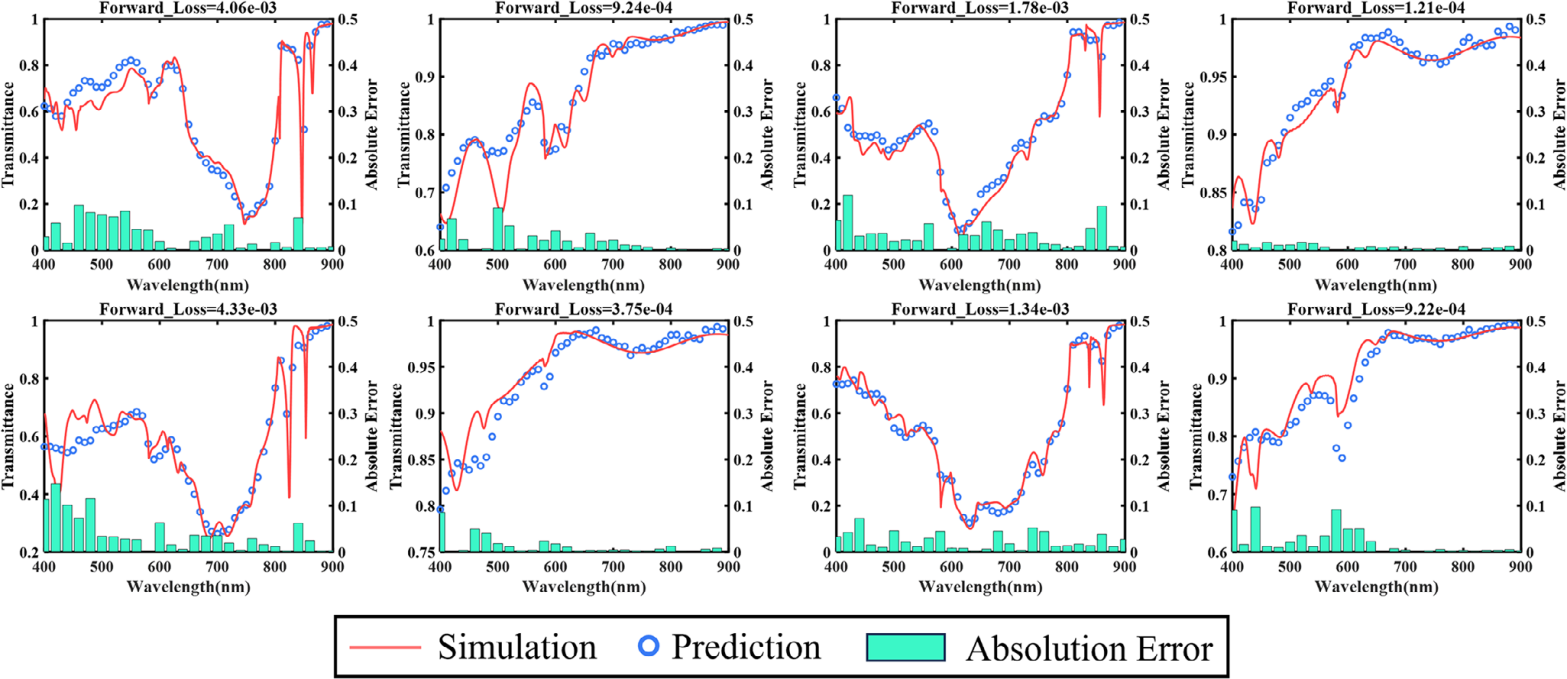
The training results of forward design network.

Independently optimizing the subnetworks corresponding to different polarization directions enhances feature representation for each specific direction while minimizing interference between polarization channels. This approach improves both prediction accuracy and the model's generalization capability [54, 55]. The 5,800 data samples were divided into a training set (80%), a validation set (10%), and a test set (10%). The model was evaluated using the test set after training (see Section S6 for details of the forward network). Figure 3 shows spectral predictions randomly selected from the test set, demonstrating that the forward network achieves high‐accuracy predictions across different spectral responses. In Section S6 (Figure S11), the predicted spectral responses for six polarization directions are shown for two different FHPEM structures, randomly selected from the dataset.

**FIGURE 4:**
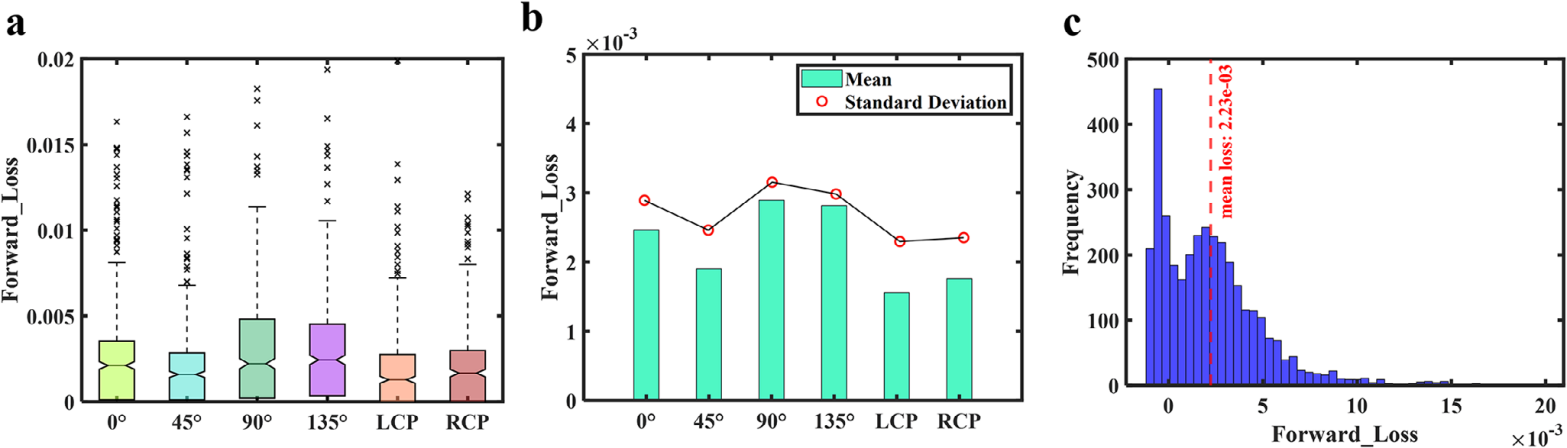
Statistical analysis of the forward design network. (a) Box plots of prediction errors for different polarization directions. (b) Mean and standard deviation of prediction errors across polarization directions. (c) Histogram of overall prediction errors across all six polarization directions.

Furthermore, a statistical analysis was conducted on the test set, and the results are presented in Figure 4. The box plots in Figure 4a indicate that the prediction errors for different polarization directions are highly consistent in terms of box length, median position, and the distribution of outliers. This result demonstrates similar error distribution patterns, comparable dispersion, and overall symmetry across all polarization directions. Figure 4b presents the mean and standard deviation of prediction errors for each polarization direction. Both the mean and standard deviation remain around 0.0025 for all polarization states. This indicates a smooth overall trend across different polarization directions and confirms the model's robustness and consistency. Figure 4c shows the histogram of the overall prediction errors across the six polarization directions. The horizontal axis represents prediction error and the vertical axis represents frequency. The error interval with the highest frequency aligns closely with the mean error of 0.0023, indicating that the prediction errors are concentrated and highly representative. Collectively, these results demonstrate that the proposed forward network can accurately learn the highly complex and nonlinear mapping between structural parameters and spectro‐polarimetric responses. This approach provides a reliable foundation for subsequent inverse design task.

#### 2.2.2 Inverse Design Network

As described above, the design of the FHPEM relies on the use of the correlation coefficient in Equation (3) to select metasurface structures with distinct spectral and polarization responses. To this end, we propose a conditional multitask inverse design network, which takes geometric parameters with latent vectors and the correlation coefficients as inputs. This network employs a conditional multitask learning strategy to predict multiple sets of geometric parameters with latent vectors that satisfy the target correlation coefficients. Furthermore, the forward and inverse networks are combined in tandem to form a composite conditional multitask tandem network, as illustrated in Figure 2. This architecture effectively addresses the “one‐to‐many” problem in inverse design [56], significantly enhancing controllability and stability. Compared to traditional trial‐and‐error approaches [28, 29, 44] or conventional machine learning methods [45, 46, 47, 48, 49, 50, 51, 52, 53, 54, 55, 56, 57], the proposed method demonstrates clear advantages in both training time and optimization efficiency [58, 59, 60, 61].

Before training the network, a new dataset was constructed to meet the requirements of the multitask inverse design network. First, the 5800 samples with latent vectors were combined in all possible pairings (total number of combinations: $C_{5800}^{2}$), denoted as Combination 1 and Combination 2, ensuring a one‐to‐one correspondence between geometric parameters and transmittance data. For each pair of transmittance data from the two combinations, the correlation coefficients of the Mueller matrix elements M_00_, M_01_, M_02_, and M_03_ were computed according to Equation (3). The absolute values were averaged (denoted as C) to quantify the overall correlation between each pair of metasurfaces. Next, for each parameter set in Combination 1 (para1), parameter sets from Combination 2 with the same correlation coefficient were selected. At least four matching sets were ensured, and four sets were randomly retained (para2). Finally, to limit the dataset size, 10,000 samples were randomly selected as the final dataset. This dataset includes para1, C, and para2 (comprising four parameter sets: para2_1, para2_2, para2_3, para2_4) (see Section S7 for a subset of the dataset).

The conditional multitask network takes para1 and C as inputs to the inverse network to predict the corresponding para2. Subsequently, para1 and para2 are fed into the forward network to obtain transmittance for different polarization directions. From this transmittance, the four sets of correlation coefficients are computed. However, using only the correlation coefficients as the loss can improve the network's ability to fit the target correlation values but does not accurately reconstruct the latent vectors with a normal distribution. This limitation makes it difficult to distinguish between different structures. To overcome this issue, a more comprehensive loss function Inverse_Loss is introduced as follows

(5) $$\begin{array}{ccc} \mathrm{Inverse}\_\mathrm{Loss} & = & \eta_{1}\cdot \frac{1}{k_{2}}\cdot \sum_{i=1}^{k_{2}}\beta_{i}^{1}\cdot {\left(y_{i}^{z}-I_{i}^{z}\left(x^{p},x^{c},w_{2},b_{2}\right)\right)}^{2}\\ & & +\;\eta_{2}\cdot \frac{1}{k_{2}}\cdot \sum_{i=1}^{k_{2}}\beta_{i}^{2}\cdot {\left(y^{c}-F_{i}^{c}\left(x^{p},x^{c},w_{3},b_{3}\right)\right)}^{2} \end{array}$$

where, *k*_2_ denotes the number of tasks (including the four parameter sets); η_1_ and η_2_ are weighting factors for the latent vectors and correlation coefficients, respectively; β^1^ and β^2^ are task‐specific weighting factors, all set to 1 to ensure balanced learning across the different parameter sets. *y^z^* represents the simulated latent vectors of the four geometric parameter sets. *I^z^* denotes the inverse network function, which takes geometric parameters *x^p^*, correlation coefficients *x^c^*, weights *w*_2_, and biases *b*_2_ as inputs and outputs the predicted latent vectors of the four parameter sets. *y^c^* represents the simulated correlation coefficients. *F^c^* denotes the tandem network function, which takes geometric parameters *x^p^*, correlation coefficients *x^c^*, weights *w*_3_, and biases *b*_3_ as inputs and outputs the predicted four sets of correlation coefficients. Ultimately, the inverse network and the tandem network output the corresponding geometric parameters and correlation coefficients, respectively. By minimizing the errors between these outputs, the tandem network backpropagates to iteratively optimize the inverse network and generate the optimal geometric parameter pairs.

**FIGURE 5:**
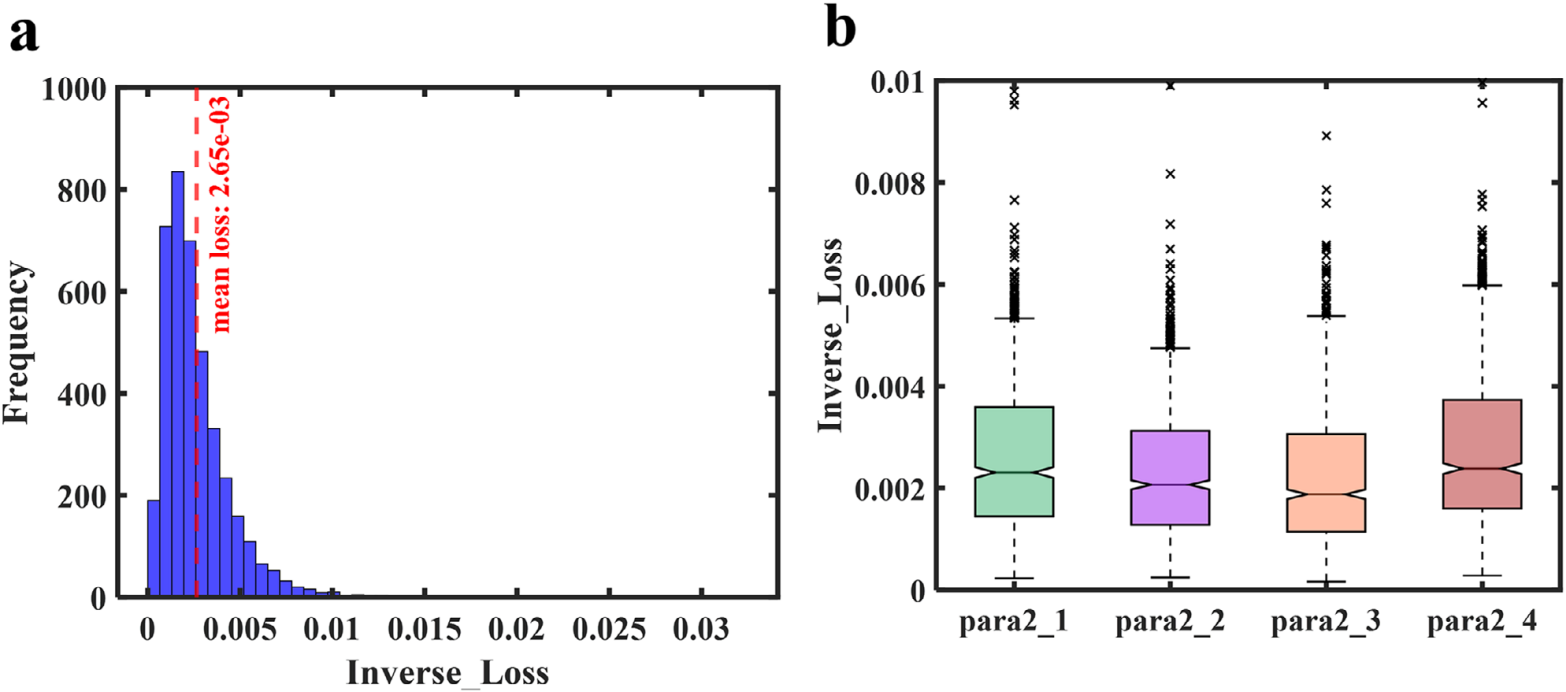
Prediction error distribution of the inverse design network. (a) Histogram of prediction errors. (b) Box plots of prediction errors for the individual predicted parameter sets.

During network training, the 10 000 data samples were divided into a training set (80%), a validation set (10%), and a test set (10%). Detailed network architecture and parameter settings are provided in Section S8. To evaluate prediction performance on the test set, the error distributions for the four target geometric parameter sets were analyzed, as shown in Figure 5. Figure 5a presents the overall distribution of prediction errors for all four parameter sets. Most errors are concentrated around 0.00265 and exhibit a relatively symmetric and narrow distribution, indicating good overall prediction accuracy and stability. Figure 5b shows the prediction error statistics for each parameter sets as box plots. The plots indicate that the prediction errors are highly consistent across all parameter sets in terms of box length, median position, and outlier distribution. Medians are near 0.002 and maximum errors are below 0.004. These results demonstrate that the model achieves robust and consistent predictive performance across multiple parameter dimensions.

**FIGURE 6:**
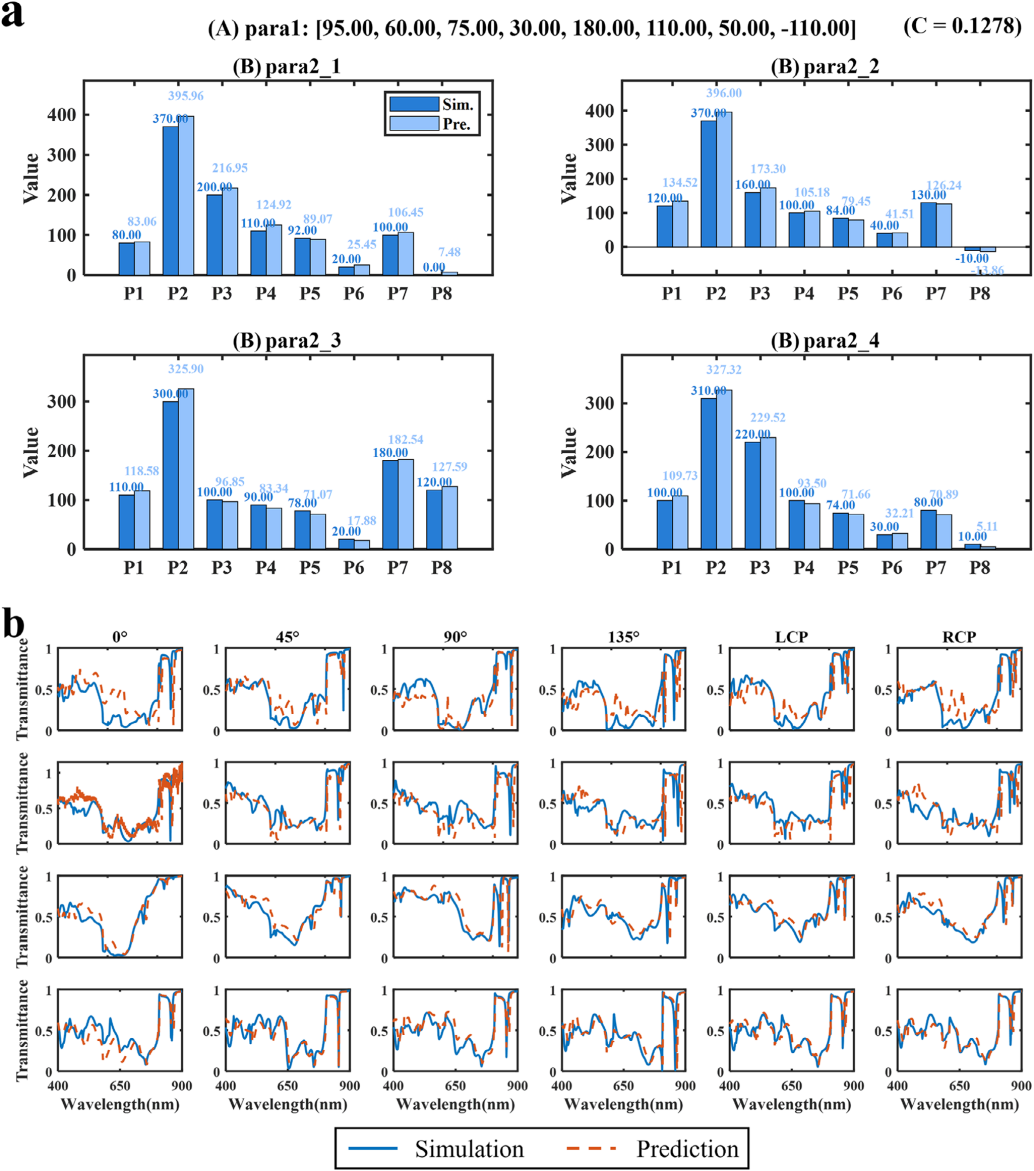
Comparison of prediction results from the inverse design network. (a) Comparison between simulated and predicted geometric parameters (units: nm). (b) Comparison of transmittance curves corresponding to the simulated and predicted parameters in (a). A and B denote two different types of metasurface structures.

Five different combinations of input parameters para1 and C were selected, and their predicted results were compared with simulated values. Figure 6a presents the prediction results for C = 0.1278, while other results are provided in Section S8, Figure S13. In the figure, A and B correspond to the two types of metasurface structures. The four predicted sets of geometric parameters closely match the true values, demonstrating that the constructed inverse design network exhibits strong generalization and stability. Notably, even under different correlation coefficient conditions, the network accurately distinguishes and predicts the corresponding structure types and parameter combinations. To verify the physical validity of the predicted parameters, the results in Figure 6a and Figure S13 were used to calculate the transmittance distributions under different polarization directions via FDTD simulations. The simulation results are shown in Figure 6b and Figure S14. They closely match the transmittance curves computed from the predicted parameters, indicating that the inverse predictions are physically realizable.

Furthermore, the correlation between the predicted parameters in Figure 6a and Figure S13 and the corresponding transmittance results in Figure 6b and Figure S14 were calculated, as summarized in Table 1. The table lists the true correlation coefficients (C_Sim) and the correlation coefficients between the four sets of predicted parameters and the input parameters para1 (C_Pre_para2_x, x = 1, 2, 3, 4). The results show that the correlation coefficients of all predicted structures are close to the true values, with a maximum deviation of only 0.0492. These findings indicate that the inverse design network not only generates reasonable structural parameters but also maintains consistency with the input structures at the level of optical response.

**TABLE 1:** Correlation coefficients between simulated and predicted geometric parameters.

	C_Sim	C_Pre_para2_1	C_Pre_para2_2	C_Pre_para2_3	C_Pre_para2_4
Figure 6a	0.1278	0.1188	0.0848	0.1093	0.1122
Figure S13a	0.2686	0.3164	0.2766	0.2861	0.2603
Figure S13b	0.3464	0.3584	0.3956	0.3359	0.3162
Figure S13c	0.4669	0.4558	0.4611	0.4591	0.4548
Figure S13d	0.5049	0.5189	0.4875	0.5231	0.5116

Therefore, the proposed conditional multitask network provides a general framework for system‐level, on‐demand design of spectro‐polarimetric encoding metasurfaces. Unlike conventional inverse design approaches that establish a single mapping from optical responses to geometric parameters, the proposed inverse network explicitly incorporates encoding correlation as a key physical constraint. A forward network is cascaded with the inverse network to evaluate the inter‐structure correlation and provide supervision during training, enabling direct control of the intercorrelation among different metasurface units at the design stage. Based on the two metasurface configurations proposed in this work, designers only need to select a structure type and specify its geometric parameters along with a target correlation coefficient. The network can then conditionally predict a matched set of geometric parameters, ensuring that the generated structure pair satisfies the prescribed correlation requirement. This process does not rely on empirical selection or large‐scale exhaustive searches and enables the automatic generation of structure pairs that meet target correlation constraints.

#### 2.2.3 Design of the FHPEM Array and Spectro‐Polarimetric Reconstruction

To verify the feasibility of the proposed network for practical metasurface arrays (superpixel) design, the trained inverse design network was employed to generate meta‐atoms. Since the network generates structural parameters that satisfy the correlation requirements only with respect to a given input parameter, additional constraints are necessary at the array level to ensure global correlation consistency. Specifically, low correlations must be maintained among the meta‐atoms within the array. To achieve this, we propose a metasurface array screening strategy based on the conditional multitask network, as illustrated in Figure 7a.

FIGURE 7Design and characterization of the FHPEM arrays. (a) Schematic illustration of the metasurface array design process based on the conditional multitask network. Here, C represents the averaged absolute correlation coefficient of the Mueller matrix elements (M_00_, M_01_, M_02_, and M_03_). (b–e) Correlation coefficient distributions of the Mueller matrix components for metasurface arrays with *n* = 16, 36, 64, and 100, respectively. The color depth indicates the degree of correlation.

**Figure:**
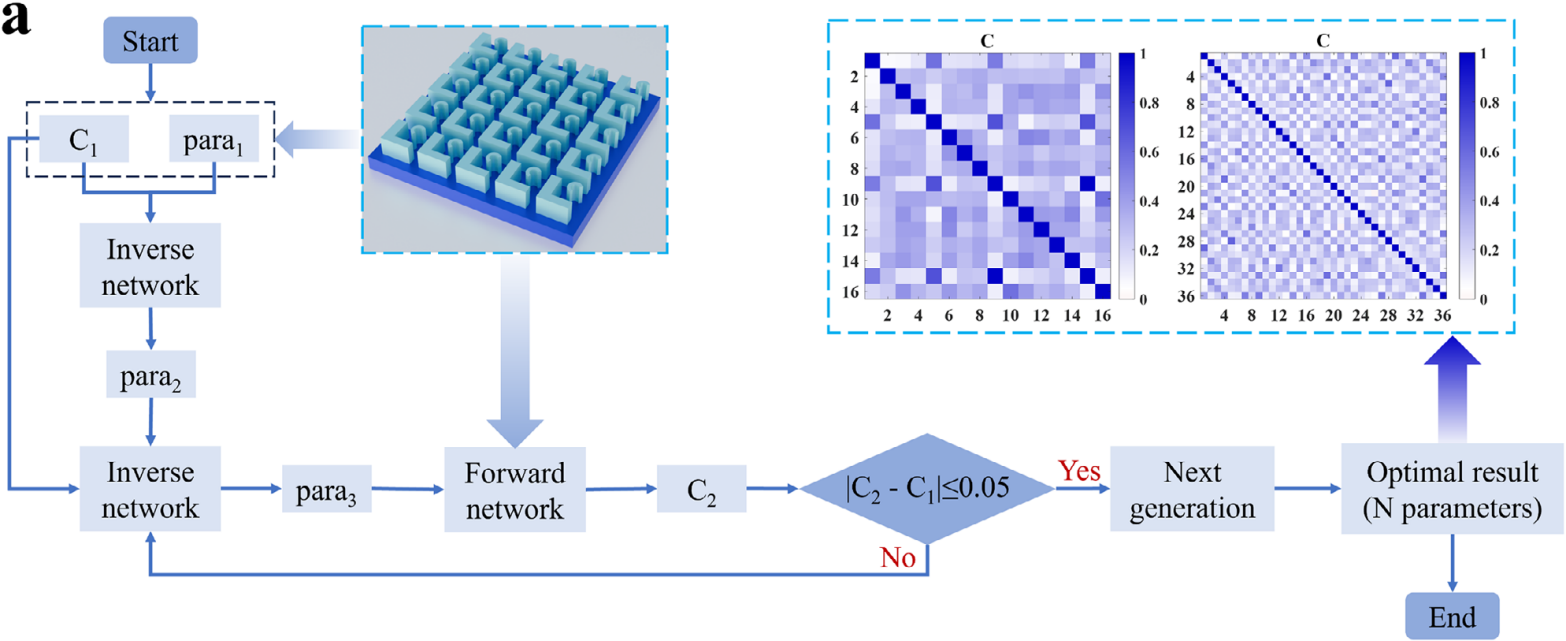

**Figure:**
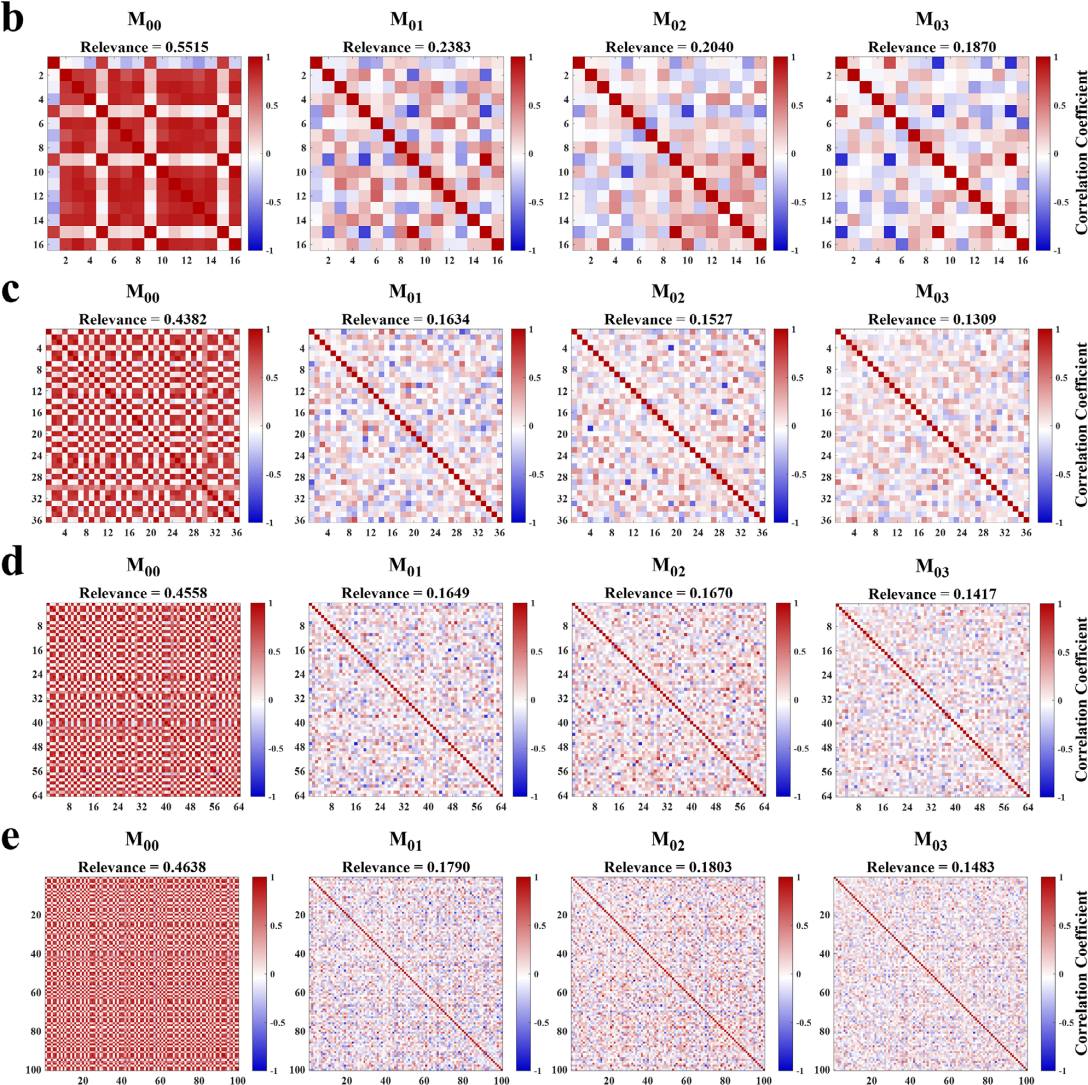

First, a set of geometric parameters para_1_ is selected and a target correlation coefficient C_1_ is defined. These are fed into the inverse design network to generate four candidate sets of geometric parameters, from which one set, para_2_, is selected. Subsequently, para_2_ and C_1_ are re‐input into the inverse network to generate four new candidates, and one set, para_3_, is chosen. Next, para_1_ and para_3_ are fed into the forward design network to calculate the corresponding correlation coefficient C_2_. Considering the maximum correlation deviation presented in Table 1, para_3_ is accepted if the absolute difference between C_2_ and C_1_ is less than 0.05. Otherwise, the process returns to the inverse design step to regenerate para_3_. This iterative procedure continues until *n* sets of geometric parameters satisfying the target correlation constraint are obtained.

Finally, using the proposed strategy, four metasurface arrays of sizes 4 × 4, 6 × 6, 8 × 8, and 10 × 10 (corresponding *n* = 16, 36, 64, and 100) were generated. The configurations of these arrays are provided in Section S9. Based on the obtained geometric parameters, the corresponding spectro‐polarimetric encoding matrix **M**_0_ was constructed. The overall performance of each array was quantitatively evaluated using the Relevance metric [45], which characterizes the degree of correlation and distinctiveness in their spectro‐polarimetric encoding behavior.

(6) $$\mathrm{Relevance}=\frac{1}{n(n-1)}\sum_{i}^{n}\sum_{j\left(i\neq j\right)}^{n}\left|C_{i,j}\right|$$

Figure 7b–e present the correlation coefficient distributions of the Mueller matrix components for the four metasurface arrays. It can be observed that the correlation coefficients of M_00_ are higher than those of M_01_, M_02_, and M_03_. As discussed in Section S1, M_00_ corresponds to the averaged intensity component, which exhibits weak polarization sensitivity and therefore limited encoding capability. In contrast, M_01_, M_02_, and M_03_ are more sensitive to polarization variations, resulting in lower correlations and stronger information distinguishability. Notably, despite the relatively high correlation in M_00_, the overall reconstruction accuracy remains unaffected.

We selected metasurface arrays from references [28, 29, 45] for comparison. References [28, 29] employed manually selected array strategies, while reference [45] adopted a machine learning‐based screening method. The comparative results are summarized in Table 2. As shown, the arrays designed by our proposed method exhibit consistently lower Relevance for M_00_, M_01_, M_02_, and M_03_ across all four array scales. Although for *n* = 16, the Relevance of M_00_ is slightly higher than that reported in [45], the remaining three components demonstrate significantly better performance, resulting in overall superior performance. Compared with the lowest Relevance reported in [45], our arrays achieve reductions of 19.7%, 34.6%, 26.9%, and 36.5% in average Relevance for the four array scales, respectively. These results further confirm that, compared with manual selection and machine learning‐based approaches, our proposed design strategy consistently produces metasurface arrays with lower Relevance.

**TABLE 2:** Comparison with existing metasurface arrays.

*N*	Refs.	Relevance (M_00_)	Relevance (M_01_)	Relevance (M_02_)	Relevance (M_03_)	Average relevance
16	[28]	0.9123	0.8342	0.7684	0.6017	0.7792
	[29]	0.8321	0.6223	0.5526	0.6728	0.6699
	[45]	0.4195	0.3186	0.4314	0.3013	0.3677
	ours	0.5515	0.2383	0.2040	0.1870	0.2952
36	[28]	0.7996	0.7054	0.6629	0.7041	0.7180
	[29]	0.6758	0.5321	0.4310	0.5567	0.5489
	[45]	0.4382	0.3842	0.3205	0.2116	0.3386
	ours	0.4382	0.1634	0.1527	0.1309	0.2213
64	[28]	0.8362	0.6679	0.7250	0.6503	0.7199
	[29]	0.7658	0.5690	0.4695	0.6390	0.6108
	[45]	0.3858	0.3658	0.2538	0.2658	0.3178
	ours	0.4558	0.1649	0.1670	0.1417	0.2324
100	[28]	0.7681	0.9394	0.8672	0.6545	0.8073
	[29]	0.9791	0.4536	0.5348	0.4226	0.5975
	[45]	0.4984	0.3705	0.3797	0.2817	0.3826
	ours	0.4638	0.1790	0.1803	0.1483	0.2429

**FIGURE 8:**
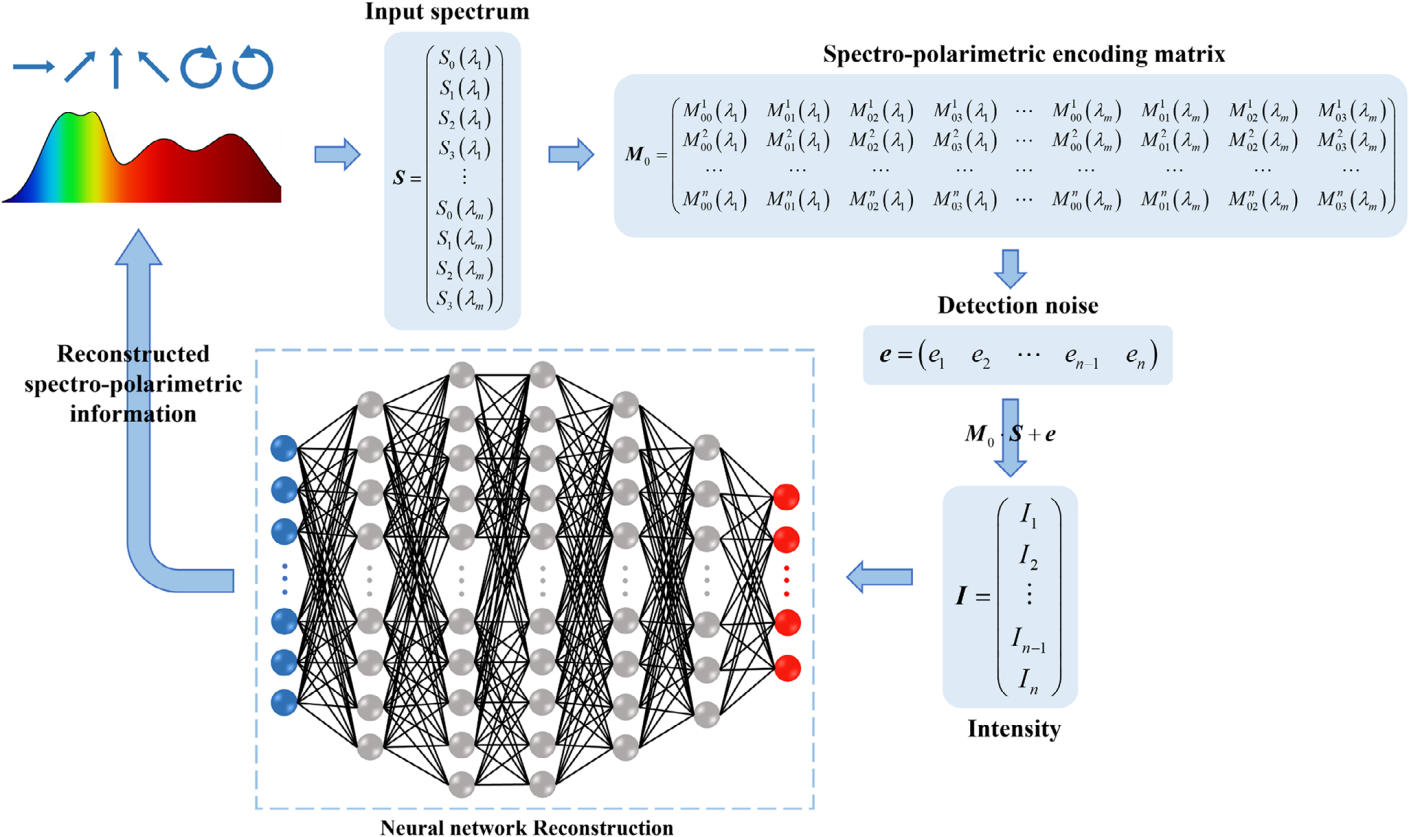
Schematic illustration of the spectro‐polarimetric reconstruction network.

To evaluate the full‐Stokes spectro‐polarimetric reconstruction performance of the generated metasurface arrays, we developed a reconstruction network, as illustrated in Figure 8. The network is composed of multiple fully connected layers and is designed to accurately reconstruct the incident full‐Stokes spectro‐polarimetric information from the measured intensity data. In the network architecture, the incident full‐Stokes spectro‐polarimetric information ***S*** is modulated by the encoding matrix **M**_0_, and the detection errors ***e*** to generate the intensity data ***I***. This intensity signal is then fed into the network, which outputs the reconstructed full‐Stokes spectro‐polarimetric information through multiple nonlinear mappings. Before training, a diverse spectro‐polarimetric dataset was constructed to facilitate model learning and evaluation. The dataset was generated by superimposing a series of Gaussian distribution functions and was divided into training (70%), validation (20%), and testing (10%) sets. Representative examples are provided in Section S10. The reconstruction accuracy was quantitatively evaluated using mean squared error (MSE), peak signal‐to‐noise ratio (PSNR), and structural similarity (SSIM) index.

**FIGURE 9:**
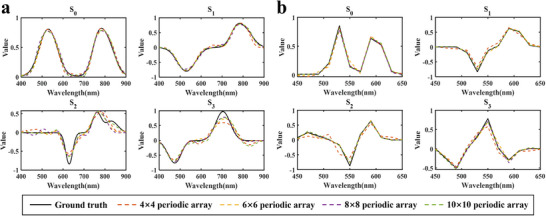
Spectro‐polarimetric information reconstruction results. (a) Reconstruction of different metasurface arrays over the wavelength range of 400–900 nm, with a spectral sampling interval of 1 nm. (b) Reconstruction results for the wavelength range of 450–650 nm, with a spectral sampling interval of 20 nm.

After completing network training, spectro‐polarimetric reconstruction was performed for the four metasurface arrays (4 × 4, 6 × 6, 8 × 8, and 10 × 10) across the wavelength range of 400–900 nm. As shown in Figure 9a, the network stably and accurately predicts the spectro‐polarimetric information for all arrays. The test wavelength range was then narrowed to 450–650 nm with a spectral sampling interval of 20 nm, and the corresponding reconstruction results are presented in Figure 9b. The results demonstrate that even under this coarser sampling conditions, the network maintains high reconstruction accuracy. These results highlight the robustness and practical applicability of the proposed method for high‐precision, narrow‐band reconstruction tasks. This capability is particularly valuable for reducing experimental acquisition costs and improving data utilization efficiency.

The MSE, PSNR, and SSIM of the reconstruction results shown in Figure 9 were calculated, and the corresponding values are summarized in Tables 3, 4, 5. Overall, for different metasurface array sizes, the reconstructed Stokes parameters exhibit low MSE, high PSNR, and high SSIM, indicating that the proposed method can accurately reconstruct spectro‐polarimetric information. As the array size increases, reconstruction performance shows an overall improvement. In particular, for the S1‐S3 components, larger metasurface arrays generally correspond to lower reconstruction errors and higher PSNR and SSIM values. This trend suggests that increasing the array size provides more degrees of freedom for the modulation and encoding of spectro‐polarimetric information, thereby enhancing the network's ability to reconstruct polarization‐related features. It should be noted that under small array configurations, some polarization components, especially S2 and S3, exhibit relatively lower SSIM values, on the order of approximately 0.5. This is mainly because SSIM is intrinsically sensitive to spatial misalignment and local structural differences. As a result, even slight spatial shifts in the reconstructed results can lead to a noticeable reduction in SSIM. In addition, the S2 and S3 components characterize the relative relationships between orthogonal polarization components. They are therefore more sensitive to noise and system imperfections. Their reconstruction is also more affected by limited array size and reduced modulation degrees of freedom, which increases the reconstruction difficulty. Nevertheless, even under these conditions, the MSE remains on the order of 0.01 and the PSNR stays around 20 dB, indicating that the network can still accurately recover the main spectro‐polarimetric information. Overall, these results confirm that the proposed structure screening strategy can effectively adapt to metasurface arrays of different scales while maintaining robust reconstruction performance under varying array sizes and spectral sampling conditions.

**TABLE 3:** MSE of reconstructed spectro‐polarimetric information for different metasurface arrays.

MSE	4 × 4 array		6 × 6 array		8 × 8 array		10 × 10 array	
	Figure 9a	Figure 9b	Figure 9a	Figure 9b	Figure 9a	Figure 9b	Figure 9a	Figure 9b
S0	0.0006	0.0004	0.0009	0.0023	0.0010	0.0005	0.0008	0.0003
S1	0.0032	0.0061	0.0009	0.0012	0.0014	0.0009	0.0007	0.0009
S2	0.0108	0.0093	0.0084	0.0014	0.0102	0.0017	0.0037	0.0016
S3	0.0098	0.0067	0.0067	0.0023	0.0032	0.0015	0.0099	0.0010

**TABLE 4:** PSNR of reconstructed spectro‐polarimetric information for different metasurface arrays.

PSNR (dB)	4 × 4 array		6 × 6 array		8 × 8 array		10 × 10 array	
	Figure 9a	Figure 9b	Figure 9a	Figure 9b	Figure 9a	Figure 9b	Figure 9a	Figure 9b
S0	30.51	32.51	28.88	25.20	28.11	31.85	29.37	34.44
S1	29.08	25.59	34.66	32.69	32.83	33.68	35.64	33.82
S2	22.09	21.94	23.79	32.15	22.93	31.43	27.17	31.54
S3	23.08	23.93	26.54	28.82	29.68	30.34	24.79	32.44

**TABLE 5:** SSIM of reconstructed spectro‐polarimetric information for different metasurface arrays.

SSIM	4 × 4 array		6 × 6 array		8 × 8 array		10 × 10 array	
	Figure 9a	Figure 9b	Figure 9a	Figure 9b	Figure 9a	Figure 9b	Figure 9a	Figure 9b
S0	0.9571	0.9467	0.9519	0.9369	0.9490	0.9963	0.9485	0.9976
S1	0.7100	0.8890	0.8675	0.9906	0.8893	0.9804	0.9038	0.9922
S2	0.5460	0.7797	0.5251	0.8837	0.5222	0.8491	0.6888	0.8492
S3	0.6580	0.7010	0.7502	0.9299	0.7886	0.8128	0.5728	0.8509

**FIGURE 10:**
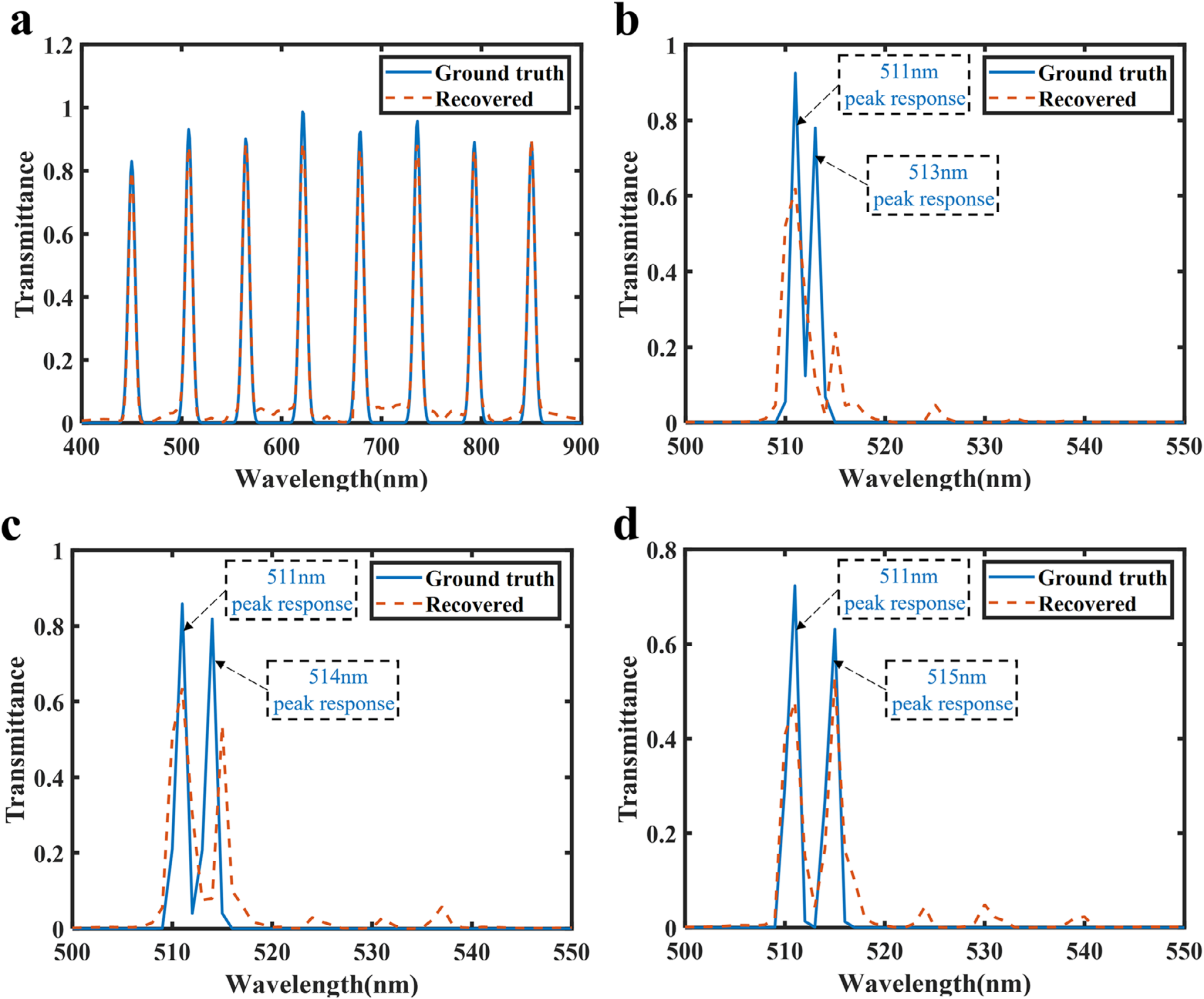
Spectral reconstruction results under 0° linearly polarized incidence. (a) Reconstruction results of the narrowband spectrum. (b–d) Reconstruction of dual‐peak sources with peak separations of 2, 3, and 4 nm, respectively.

As shown in Tables 3, 4, 5, although the MSE, PSNR, and SSIM of the 4 × 4 array are slightly lower than those of the 6 × 6, 8 × 8, and 10 × 10 arrays, the reconstruction accuracy remains sufficiently high, with MSE below 0.01, PSNR above 20 dB, and SSIM exceeding 0.5. However, increasing the array size generally reduces spatial resolution [62] and increases fabrication complexity and cost. Therefore, the 4 × 4 array was selected for further spectral analysis and subsequent experimental validation. Figure 10 presents the reconstruction results under 0° linearly polarized light incidence. In Figure 10a, multiple narrowband spectra are reconstructed with high precision, showing minimal cross‐talk between spectral components even when multiple peaks are present. Figure 10b–d illustrate the reconstruction of dual‐peak sources with peak separations of 2, 3, and 4 nm, respectively. The reconstruction accuracy improves as the peak separation increases. At a 3 nm separation, the dual‐peak feature is partially resolved, though slight peak shifts still cause some reconstruction error. At a 4 nm separation, the dual peaks are clearly distinguished, and the reconstructed peak positions closely match the ground truth. These results indicate that the spectral resolution of the designed 4 × 4 metasurface array reaches 4 nm.

#### 2.2.4 Experimental Characterization of Designed FHPEM Array

**FIGURE 11:**
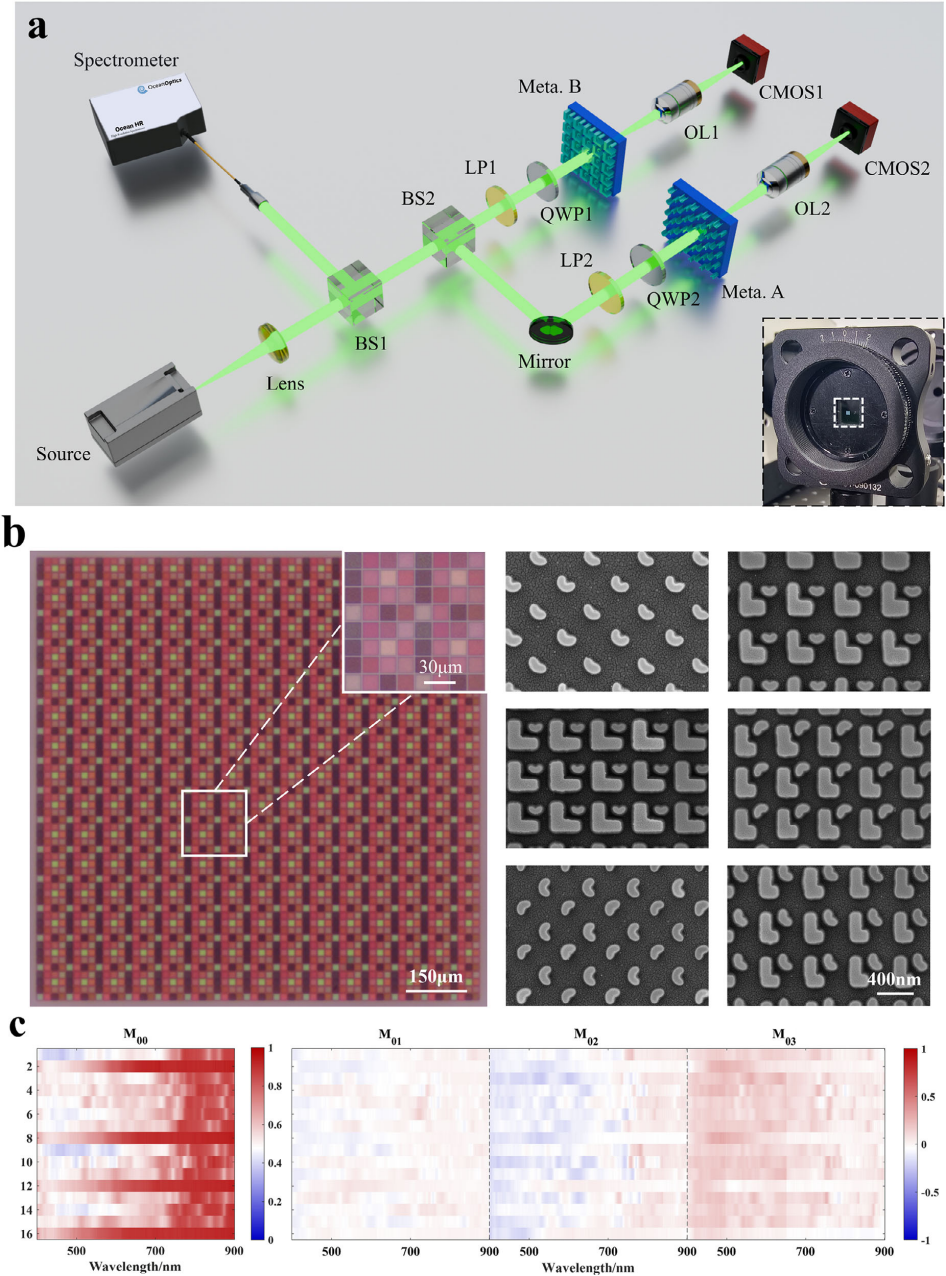
Experimental characterization of the metasurface sample. (a) Schematic of the spectro‐polarimetric measurement setup. The light source controls the incident spectral profile; a spectrometer measures the spectral information; a beam splitter (BS) separates and directs light to different optical paths; a linear polarizer (LP) and a quarter‐wave plate (QWP) control the polarization state of the incident light; an objective lens (OL) collects the transmitted light through the metasurface; and CMOS camera images the back focal plane. (b) Optical image (left) and SEM image (right) of the metasurface sample. (c) Experimentally measured spectro‐polarimetric encoding matrix for the selected 16 meta‐atoms.

To experimentally validate the spectro‐polarimetric reconstruction performance of the 4 × 4 metasurface array designed using the proposed structure screening strategy, the sample was fabricated via electron‐beam lithography. The detailed fabrication process is provided in Section S11. The fabricated metasurface sample covered an area of 1020 × 1020µm^2^ and consisted of 15 × 15 superpixels. An experimental setup was constructed to measure the spectral responses of the sample under different polarization states, as shown in Figure 11a, with additional details given in Section S12. Figure 11b shows the optical and scanning electron microscopy (SEM) images of the metasurface sample, while SEM images of 16 meta‐atoms are shown in Section S13. By testing different meta‐atoms within the metasurface, the corresponding spectro‐polarimetric responses were obtained, and the spectro‐polarimetric encoding matrix **M**_0_ was calculated, as shown in Figure 11c.

**FIGURE 12:**
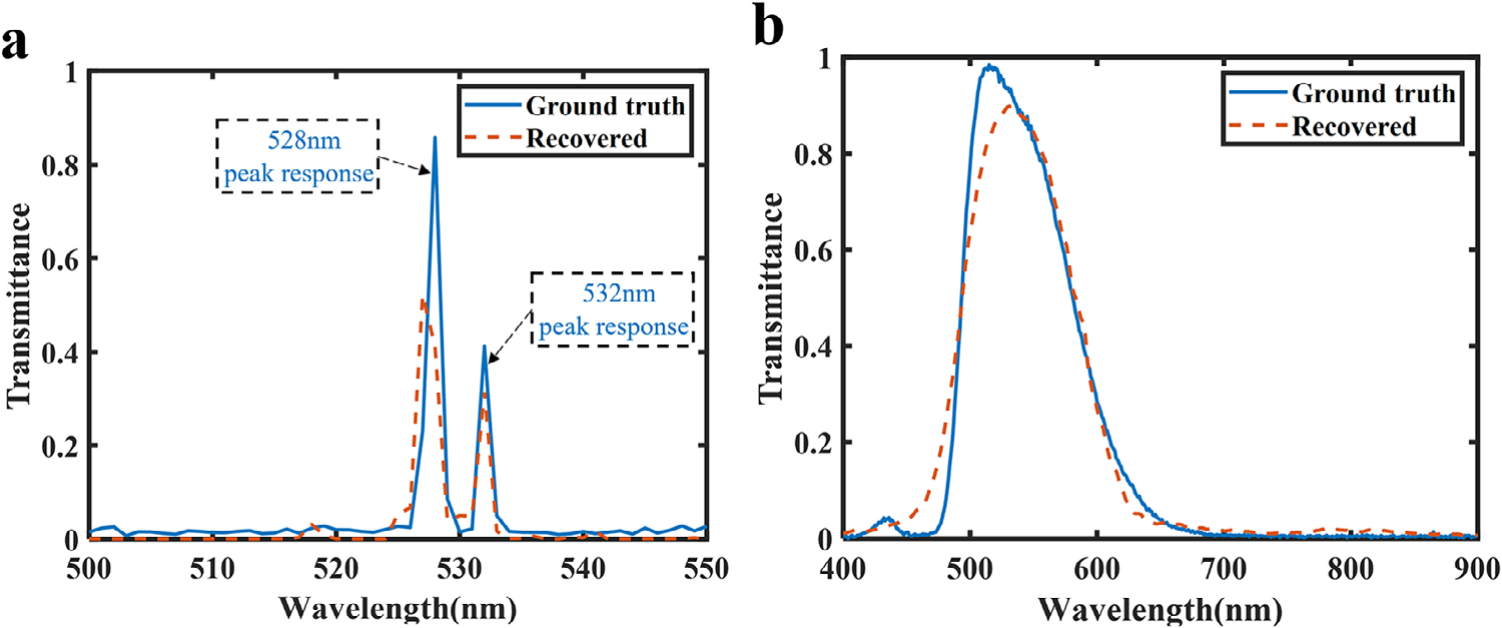
Spectral reconstruction results. (a) Verification of spectral resolution under 0° linearly polarized incidence. (b) Broadband spectral reconstruction under 0° linearly polarized incidence.

After obtaining the spectro‐polarimetric encoding matrix **M**_0_, the spectral resolution of the metasurface sample was experimental verified. Under 0° linearly polarized light, a combination of 528 and 532 nm sources was generated using a beam splitter and transmitted through the sample, with the transmitted light detected by a photodetector. The experimental setup is described in Section S15. The reconstructed spectra are shown in Figure 12a. At a spectral resolution of 4 nm, the designed metasurface accurately reconstructs the incident spectral information, shown good agreement with the ground truth. Compared with the 6 nm simulation results in Ref. [45] and the 7.5 nm experimental results in Ref. [28] , the metasurface arrays designed in this work exhibit superior spectral resolution performance.

Subsequently, under 0° linearly polarized light, a broadband source was incident on the metasurface, and the reconstructed spectra are presented in Figure 12b. The reconstructed results closely match the ground truth, with an MSE of 0.0025, a PSNR of 25.94 dB, and an SSIM of 0.7879. To further evaluate the spectral resolution and reconstruction accuracy under different polarization conditions, we also performed measurements with 45°, 90°, and 135° linear polarizations, as well as left‐ and right‐handed circular polarizations (LCP and RCP). The corresponding reconstruction results are provided in Section S16. The optimal MSE, PSNR, and SSIM achieved were 0.0017, 27.48 dB, and 0.8611, respectively. These results further confirm the robustness and consistency of the proposed spectro‐polarimetric encoding metasurface arrays across different polarization states. Despite variations in the incident polarization, the encoding arrays consistently provide sufficient and stable spectro‐polarimetric modulation, ensuring the effectiveness of the reconstruction algorithm under diverse polarization conditions.

Finally, an experimental setup was built to perform spectro‐polarimetric reconstruction. Due to experimental constraints, two fixed‐wavelength light sources were used, and different polarization combinations were generated by adjusting a linear polarizer and a quarter‐wave plate. The details of the experimental configuration are provided in Section S15. The reconstruction results for various polarization combinations are shown in Figure 13. Specifically, (a) corresponds to 0° and 45° linear polarization; (b) to 0° and 90° linear polarization; (c) to 45° and 90° linear polarization; (d) to 90° linear polarization and right circular polarization (RCP); (e) to 135° linear polarization and left circular polarization (LCP); and (f) to RCP and LCP.

FIGURE 13Spectro‐polarimetric reconstruction results for different polarization combinations. (a) 0° and 45° linear polarizations. (b) 0° and 90° linear polarizations. (c) 45° and 90° linear polarizations. (d) 90° linear polarization and RCP. (e) 135° linear polarization and LCP. (f) RCP and LCP.

**Figure:**
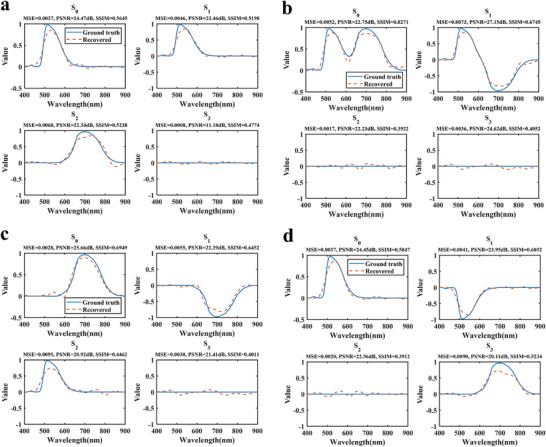

**Figure:**
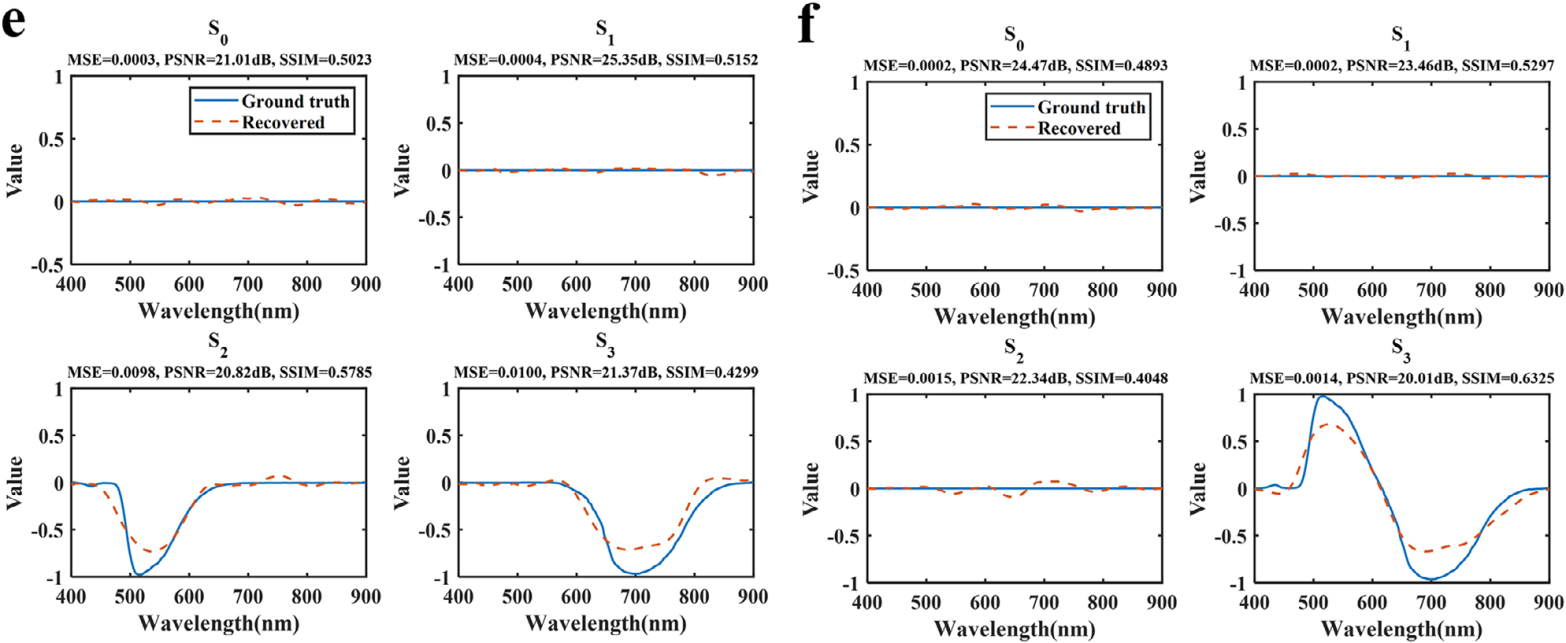

As shown in the Figure 13, the metasurface samples achieve high‐precision reconstruction across various polarization combinations. For all experimental results, the MSE remains below 0.1, the PSNR exceeds 20 dB and the SSIM is higher than 0.39, indicating robust and consistent performance under different polarization conditions. A closer comparison reveals that combinations of two linear polarizations, as in (a–c), generally yield higher reconstruction accuracy than those involving circular polarization. This suggests that linear polarization pairs provide slightly better information redundancy and signal stability under the current experimental setup. Although mixed linear and circular polarization inputs exhibit a modest decrease in reconstruction performance, the corresponding MSE, PSNR, and SSIM still reach 0.0100, 20.11 dB, and 0.3912, respectively, confirming the stability and reliability of the proposed method for diverse polarization states. Moreover, the combination of right‐ and left‐circular polarization (i.e., fully circular input) still achieves an MSE of 0.0015, a PSNR of 20.01 dB, and an SSIM of 0.4048, demonstrating the metasurface's ability to accurately reconstruct arbitrary polarization inputs.

To further validate the spectro‐polarimetric imaging performance of the metasurface array, outdoor experiments were conducted on different target scenes. During dataset construction, the same targets were imaged using a pushbroom spectro‐polarimetric imaging system [18] and the FHPEM imaging system. To obtain circular polarization information, a quarter‐wave plate with a pre‐calibrated fast‐axis orientation was placed in front of the pushbroom system [63]. In total, 500 encoded images and their corresponding spectral and polarization images were obtained. The experimental setup and data processing procedures are provided in Section S17. After dataset construction, the encoded images and the corresponding spectro‐polarimetric images were used as the input and output of the reconstruction network, respectively. The network architecture is described in Section S18. Figure 14 presents the reconstructed spectro‐polarimetric images of different target scenes from 450 to 850 nm with a wavelength interval of 100 nm. The ground‐truth spectro‐polarimetric images are shown in Figure S25.

FIGURE 14Spectro‐polarimetric image reconstruction results for different target scenes. The original images show the RGB images of the corresponding targets.

**Figure:**
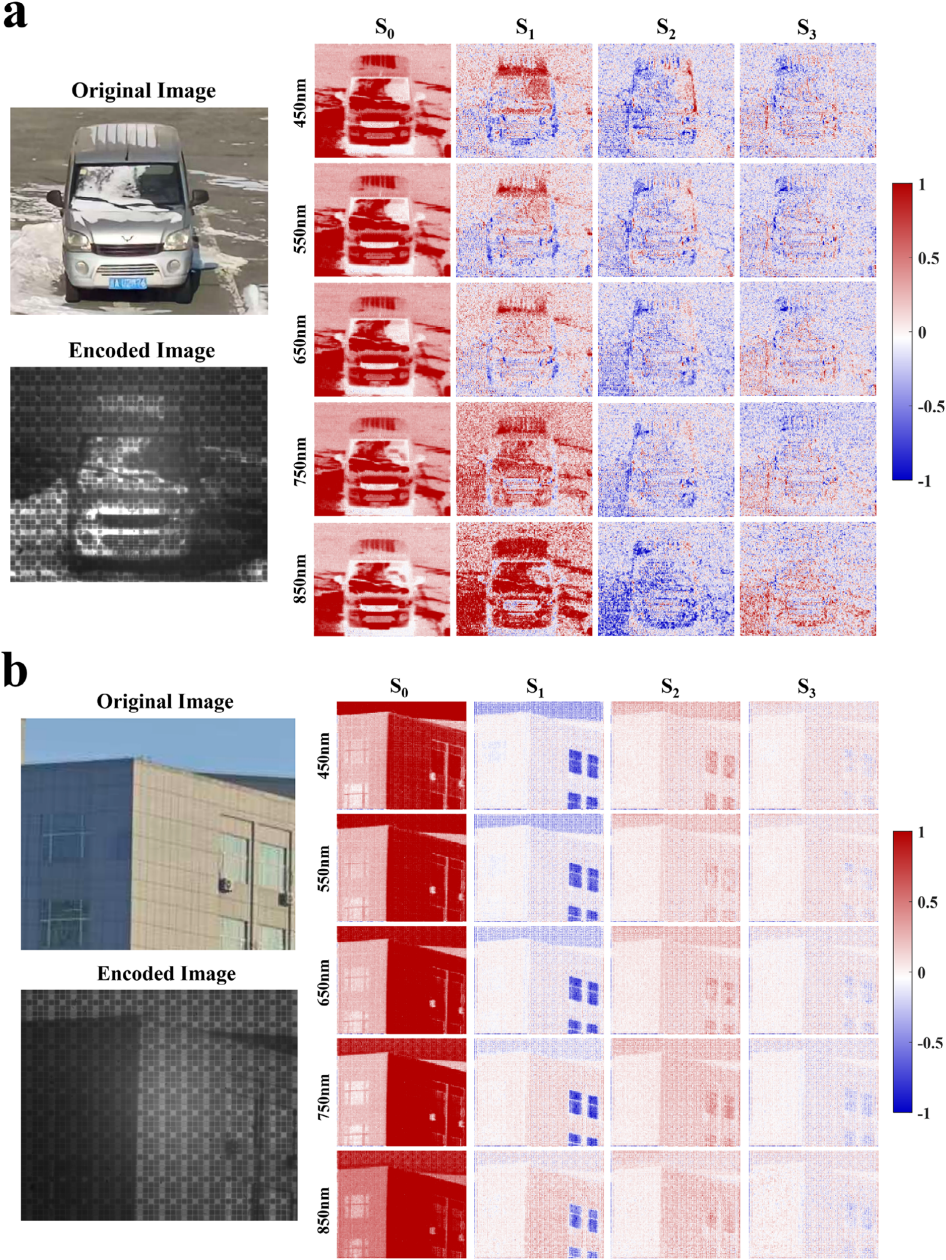

**Figure:**
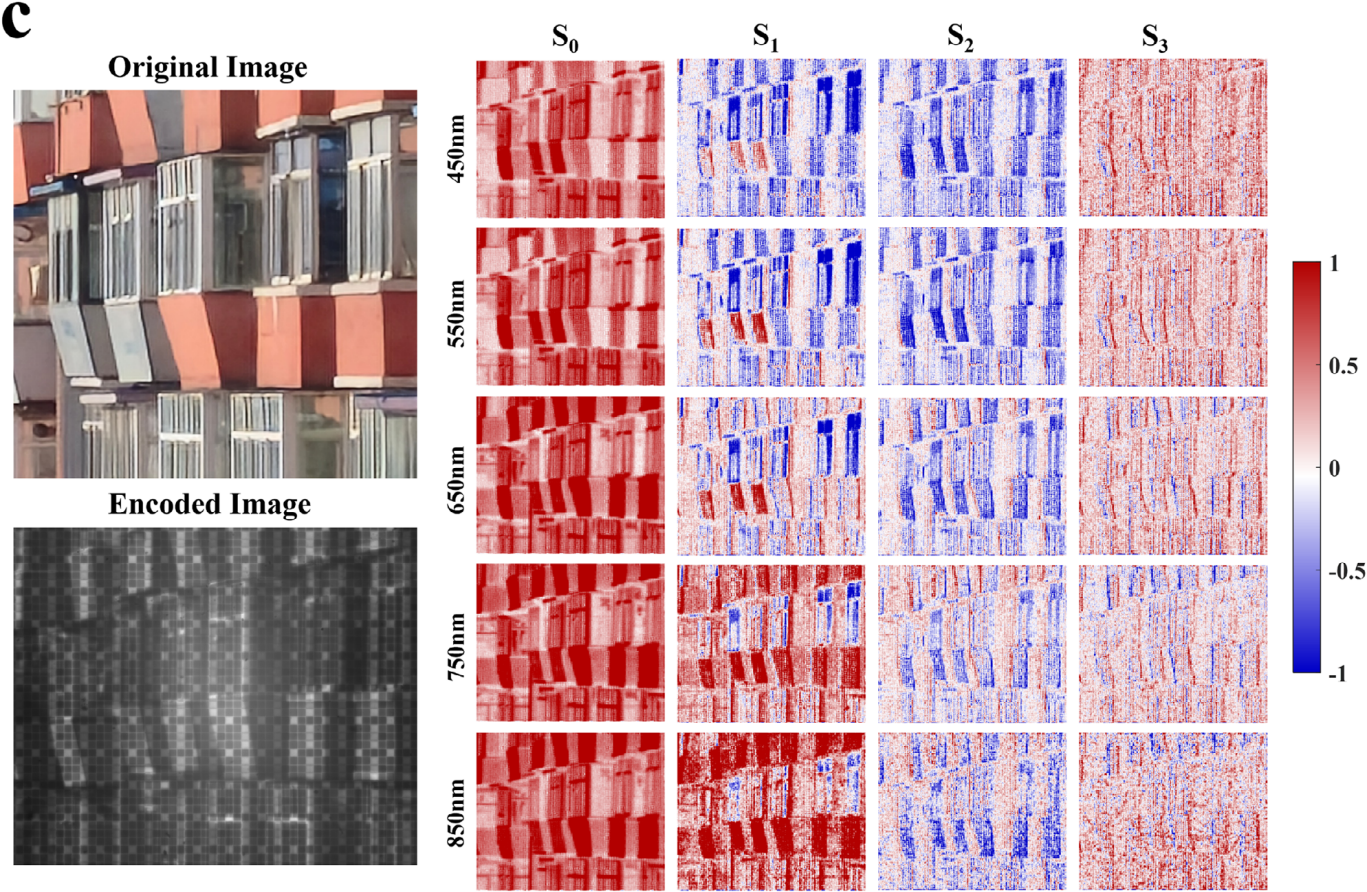

As shown in the figure, the designed metasurface array enables effective spectro‐polarimetric image reconstruction across all three target scenes. In particular, for the vehicle shown in Figure 14a, the blue license plate exhibits clear spectral discrimination at 550 nm, along with consistent polarization contrast. These results indicate that the proposed encoding metasurface can accurately capture subtle spectro‐polarimetric features of man‐made objects. For the building scene in Figure 14b,c, the window regions exhibit strong polarization‐dependent responses and are consistently reconstructed in the corresponding Stokes images, indicating accurate recovery of polarization information. Meanwhile, the reddish wall regions of the building mainly present distinct spectral characteristics, which are faithfully reconstructed across the visible spectrum. Together, these results demonstrate that the proposed metasurface array is capable of simultaneously capturing and reconstructing both polarization‐sensitive and spectrally distinctive features in complex scenes, highlighting its robustness and versatility for spectro‐polarimetric imaging applications.

**FIGURE 15:**
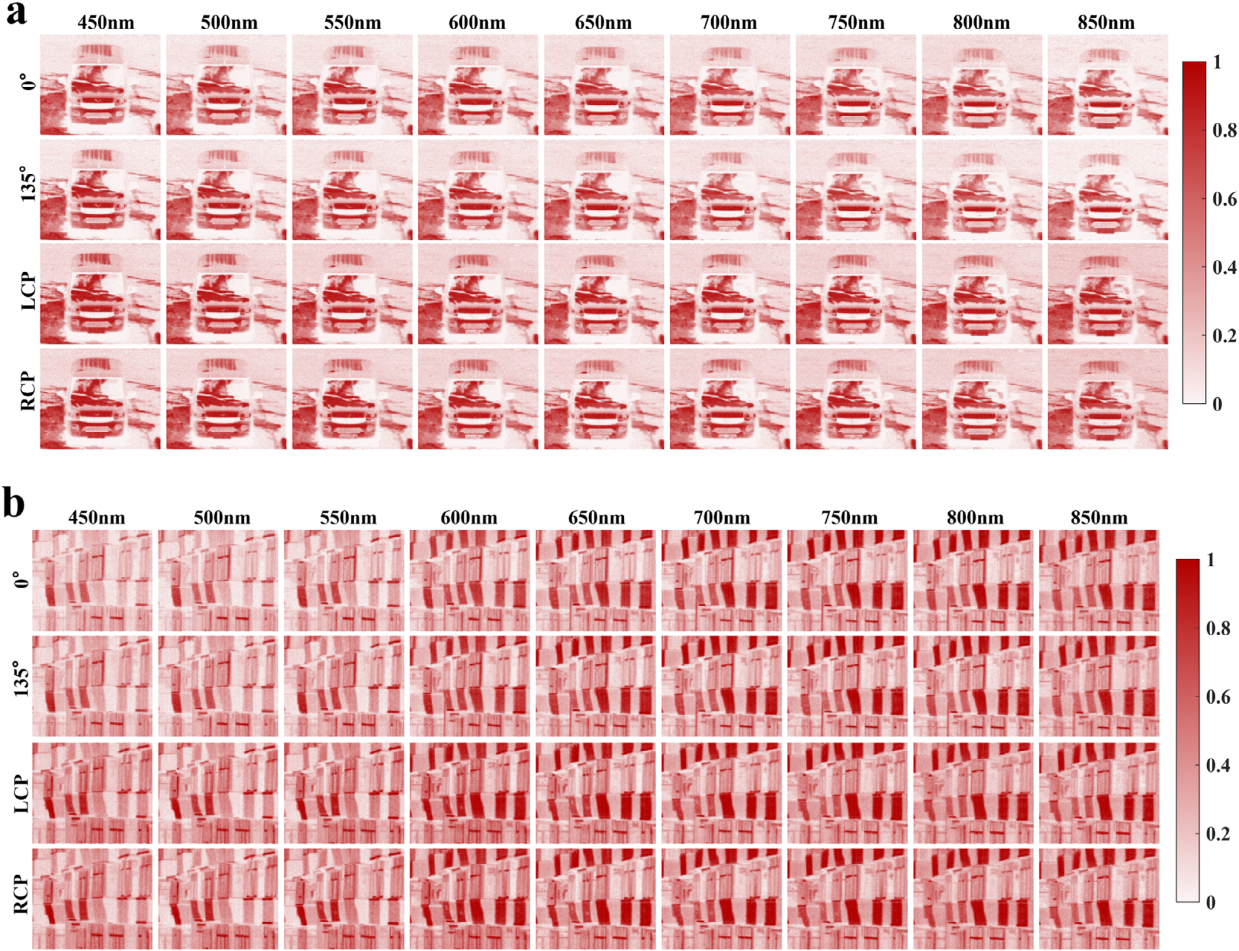
Polarization‐resolved spectral reconstructions under four representative polarization states (0°, 135°, LCP, and RCP).

To further examine the polarization‐resolved imaging capability of the proposed metasurface array, independent spectral reconstructions under specific polarization states were additionally performed. The typical target scenes shown in Figure 14a,c were selected for this analysis. In particular, spectral images corresponding to four representative polarization states, including 0°, 135°, left‐handed circular polarization (LCP), and right‐handed circular polarization (RCP), were reconstructed over a wavelength range from 450 to 850 nm with a spectral interval of 50 nm. The reconstructed results are shown in Figure 15, while the corresponding ground‐truth images and the architecture of the reconstruction network are provided in Figure S26 and Section S21, respectively.

The reconstructed results reveal clear polarization‐dependent spectral contrasts across the scene, indicating that the proposed encoding metasurface can effectively decouple and recover different polarization components with high spectral fidelity. In particular, the spectral features of the vehicle license plate in Figure 15a are well recovered, while the reddish wall in Figure 15b is also faithfully reconstructed across the visible spectrum. Moreover, the polarization‐dependent differences of the glass regions in Figure 15b are captured with good fidelity, demonstrating effective recovery of subtle polarization information. These polarization‐resolved spectral reconstructions cover the fundamental polarization components required for ellipsometric parameter extraction, and in some cases, the reconstruction quality exceeds that obtained from the full‐Stokes spectral images. Overall, these results verify the imaging robustness and generalization capability of the proposed spectro‐polarimetric encoding metasurface array in complex outdoor environments, highlighting its potential for advanced spectro‐polarimetric and ellipsometric imaging applications.

Overall, the proposed design approach goes beyond a simple encoding‐decoding scheme for spectro‐polarimetric information [64], and associated design network is fundamentally different from conventional forward and inverse design methods [33, 34]. The core contribution of this work lies in the deep integration of physical constraints inherent to the encoding process with a deep learning‐based design network, thereby establishing an on‐demand generative metasurface design paradigm tailored for spectro‐polarimetric systems. This constraint‐driven, unified network architecture significantly improves the efficiency, controllability, and stability of multi‐channel spectro‐polarimetric metasurface design. Specifically, based on system‐level requirements for encoding independence, channel number, and fabrication feasibility, a target correlation coefficient can be predefined and jointly used with initial geometric parameters as conditional inputs to the network. The network automatically learns the mapping among input geometries, correlation constraints, and output geometries, enabling direct generation of spectro‐polarimetric encoding metasurface arrays with low inter‐element correlation while satisfying fabrication constraints at the design stage. Compared with traditional design strategies that lack explicit correlation control and therefore rely heavily on manual screening, the proposed approach substantially enhances both the efficiency and controllability of high‐dimensional encoding metasurface array design.

## 3 Conclusion

In conclusion, this work proposes two novel full‐Stokes hyperspectro‐polarimetric encoding metasurface (FHPEM) structures and a conditional multitask, end‐to‐end deep learning framework that explicitly incorporates encoding‐related physical constraints. By introducing conditional information, the proposed framework enables collaborative and efficient optimization of the two metasurface types. The forward prediction and inverse design networks are jointly leveraged to establish an effective metasurface array screening strategy, which allows direct regulation of inter‐element correlation during array construction while avoiding reliance on empirical tuning or exhaustive search. Using the proposed framework, metasurface arrays with various scales, including 4 × 4, 6 × 6, 8 × 8, and 10 × 10, are successfully designed. Compared with the lowest Relevance values reported in existing metasurface array generation methods, the average Relevance is reduced by 19.7%, 34.6%, 26.9%, and 36.5%, respectively. Spectro‐polarimetric reconstruction experiments based on the 4 × 4 metasurface array demonstrate high‐accuracy spectral and polarization image reconstruction over the 400–900 nm wavelength range, achieving a spectral resolution of 4 nm. These results confirm the feasibility and effectiveness of the complete structure‐design‐reconstruction pipeline proposed in this work.

In summary, the proposed conditional multitask network provides an automated, scalable, and physically constrained solution for the array design of spectro‐polarimetric encoding metasurfaces. Unlike existing approaches that mainly rely on empirical screening or discrete search strategies based on genetic algorithms, this work emphasizes the system‐level designability and scalability of encoding metasurfaces. The proposed framework eliminates the need for empirical exploration and repeated random optimization within large structural libraries. It enables collaborative optimization of multiple metasurface types within a unified design framework and supports on‐demand generation of low‐correlation encoding arrays under predefined target correlation constraints. The proposed method demonstrates superior performance in both array correlation metrics and final spectro‐polarimetric reconstruction accuracy compared with existing approaches. More importantly, this work extends the application scope of deep learning in spectro‐polarimetric encoding metasurface design and marks a critical transition from encoding performance validation toward system‐level automated array design. This paradigm offers a new promising pathway toward large‐scale, high‐precision spectro‐polarimetric sensing systems.

## 4 Experimental Section

### 4.1 Fabrication Details

The complete fabrication process is provided in Section S11. The detailed steps are as follows: (1) Cleaning of SiO_2_ substrate: The SiO_2_ substrate was immersed in acetone and ultrasonically cleaned for 3 min to remove organic contaminants. It was then rinsed with isopropanol to eliminate residual acetone and other organic residues, followed by deionized water rinsing to remove inorganic salts and particulates. Finally, the substrate was dried using a nitrogen gun. (2) Si_3_N_4_ deposition: A 600 nm thick silicon nitride (Si_3_N_4_) film was deposited on the cleaned substrate using plasma‐enhanced chemical vapor deposition (PECVD), with silane (SiH_4_), ammonia (NH_3_), and nitrogen (N_2_) serving as the reaction gases. The deposition time and parameters were precisely controlled to ensure uniform film thickness and surface quality. (3) Photoresist coating: The substrate was mounted on a spin coater, and an appropriate amount of ZEP520 positive photoresist was dispensed onto its surface. The resist was distributed via spin coating, followed by prebaking at 90°C –120°C for 2 min to harden the photoresist. (4) Electron‐beam lithography (EBL): The sample was loaded into an electron‐beam lithography system. Based on the designed pattern, the beam current, exposure dose, and scanning speed were set accordingly. After exposure, the sample was developed in a developer solution to remove the exposed resist regions, rinsed with isopropanol to stop the development process, and dried with nitrogen. (5) Reactive ion etching (RIE): The developed sample was placed into a reactive ion etching (RIE) system. Using CHF_3_ as the etching gas, the exposed silicon nitride layer was etched. The remaining photoresist served as a mask to protect the unexposed areas from etching. (6) Photoresist removal: After etching, the sample was immersed in methyl isobutyl ketone (MIBK) solution and ultrasonically cleaned for 5–10 min to completely remove the photoresist. It was then rinsed with isopropanol to remove residual MIBK, followed by deionized water rinsing, and finally dried with nitrogen. (7) Dicing and characterization: The etched sample was coated with UV‐curable adhesive for surface protection and then diced into the required dimensions using a precision dicing saw. The UV adhesive and cutting debris were removed by acetone cleaning, followed by deionized water rinsing. The final samples were characterized using a scanning electron microscope (SEM) to examine morphology, linewidth, and edge roughness.

### 4.2 Measurement Details

The measurement setup for the Full‐Stokes Hyperspectro‐Polarimetric Encoding Metasurface employed a monochromator (Zolix, Omni‐λ3047i) as the incident light source. The output light sequentially passed through a linear polarizer (LP) and a quarter‐wave plate (QWP) to produce monochromatic light with various polarization states. The polarized light was then directed onto the metasurface, collected by an objective lens, and finally detected by a photodetector.

To ensure the wavelength accuracy of the monochromator output, a beam splitter was placed in front of the monochromator to direct a portion of the emitted beam into a spectrometer (Ocean Optics, Ocean HR2) for real‐time wavelength calibration. By adjusting the monochromator, LP, and QWP, transmittance spectra were measured over the wavelengths range of 400–900 nm under 0°, 45°, 90°, and 135° linear polarization, as well as left‐ and right‐circular polarizations.

### 4.3 Calculation

All the calculations and neural network training were performed on a Windows PC with the hardware: CPU: Intel Core i7‐12700KF; RAM: 24 GB; GPU: NVIDIA RTX 4090. The involved software packages include Python 3.9, Pytorch 2.5.1.

## Conflicts of Interest

The authors declare no conflict of interest.

## Supporting information




**Supporting File**: advs74470‐sup‐0001‐SuppMat.docx

## Data Availability

The data that support the findings of this study are available from the corresponding author upon reasonable request.
