# Supplementary material for: Design and Optimization of Full‐Stokes Hyperspectro‐Polarimetric Encoding Metasurfaces Based on Conditional Multi‐Task Deep Learning
Source: Adv Sci (Weinh). 2026 Feb 17;13(24):e23143. doi: 10.1002/advs.202523143 (PMC13116081; doi:10.1002/advs.202523143)
Supplement: Supplementary file 1 — Supporting File: advs74470‐sup‐0001‐SuppMat.docx [file ADVS-13-e23143-s001.docx]

Supplementary Information for

**Design and Optimization of Full-Stokes Hyperspectro-Polarimetric Encoding Metasurfaces Based on Conditional Multi-Task Deep Learning**

Chenjie Gong^1,3^, Haodong Shi^*,1,3^, Qi Wang^1,3^, Yingchao Li^1,3^, Hongyu Sun^1,3^, Jiayu Wang^1,3^, Guanlin Li^1,3^, Jian Zhang^2,3^, Qiang Fu^1,3^, Huilin Jiang^1,3^

^1^Jilin Provincial Key Laboratory of Space Optoelectronics Technology, Changchun University of Science and

Technology, Changchun 130022, Jilin, China.

^2^Jilin Engineering Research Center of Photoelectric Measurement & Control Instruments, Changchun 130022, Jilin, China.

*^3^School of Optoelectronic Engineering, Changchun University of Science and Technology, Changchun 130022, Jilin, China.*

**Corresponding author:* [*shihaodong08@163.com*](mailto:shihaodong08@163.com) *(H. Shi)*

**Content:**

[**Supplementary Note 1: Theoretical modeling of the FHPEM. 3**](#_Toc220332189)

[**Supplementary Note 2: Design of metasurface structure and dataset generation. 4**](#_Toc220332190)

[**Supplementary Note 3: Analysis of the electric field distribution in the metasurface structure. 6**](#_Toc220332191)

[**Supplementary Note 4: Analysis of latent vector. 8**](#_Toc220332192)

[**Supplementary Note 5: AE network training. 10**](#_Toc220332193)

[**Supplementary Note 6: Forward network training. 13**](#_Toc220332194)

[**Supplementary Note 7: Inverse network dataset. 14**](#_Toc220332195)

[**Supplementary Note 8: Inverse network training. 15**](#_Toc220332196)

[**Supplementary Note 9: Arrays of the FHPEM. 19**](#_Toc220332197)

[**Supplementary Note 10: Dataset for the reconstruction network. 20**](#_Toc220332198)

[**Supplementary Note 11: Fabrication Details of the FHPEM. 20**](#_Toc220332199)

[**Supplementary Note 12: Experimental Setup. 21**](#_Toc220332200)

[**Supplementary Note 13: The SEM images of the metasurface sample. 21**](#_Toc220332201)

[**Supplementary Note 14: Experimental setup for spectral resolution. 22**](#_Toc220332202)

[**Supplementary Note 15: Experimental setup for the information reconstruction. 22**](#_Toc220332203)

[**Supplementary Note 16: Validation of spectral resolution and reconstruction accuracy under different polarization states. 23**](#_Toc220332204)

[**Supplementary Note 17: Experimental setup for spectro-polarimetric imaging and dataset construction. 25**](#_Toc220332205)

[**Supplementary Note 18: Spectro-Polarimetric Image Reconstruction Network. 26**](#_Toc220332206)

[**Supplementary Note 19: Ground-truth spectro-polarimetric images for different target scenes. 26**](#_Toc220332207)

[**Supplementary Note 20: Ground-truth polarization-resolved spectral images for different target scenes. 28**](#_Toc220332208)

[**Supplementary Note 21: Network architecture for polarization-resolved spectral image reconstruction. 29**](#_Toc220332209)

[**Reference 30**](#_Toc220332210)

**Supplementary Note 1: Theoretical modeling of the FHPEM.**

A mathematical model is first established to elucidate the spectral and polarization encoding mechanism of the metasurface, thereby providing theoretical guidance for structural design. In general, the polarization state of light can be characterized by the Stokes parameters $S=\left[S_{0},S_{1},S_{2},S_{3}\right]$, which comprehensively describe the complete polarization information of the incident light. The Stokes parameters are expressed as follows

$$\begin{matrix} S_{0}=I_{0^{\circ}}+I_{90^{\circ}}=I_{45^{\circ}}+I_{135^{\circ}}=I_{R}+I_{L}, \\ S_{1}=I_{0^{\circ}}-I_{90^{\circ}}, \\ S_{2}=I_{45^{\circ}}-I_{135^{\circ}}, \\ S_{3}=I_{R}-I_{L} \end{matrix}$$

where, $I_{0^{\circ}},I_{90^{\circ}},I_{45^{\circ}},I_{135^{\circ}}$ represent the intensities under linear polarizations at 0°, 90°, 45°, and 135°, respectively, while $I_{R},I_{L}$ denote the intensities of right- and left-handed circularly polarized light (RCP and LCP). Therefore, at wavelength $\lambda$, the input and output full-Stokes spectro-polarimetric information can be related through the Mueller matrix $\mathbf{M}\left(\lambda\right)$, which can be expressed as follows

$$S^{'}\left(\lambda\right)=\left(\begin{matrix} S_{0}^{'}\left(\lambda\right) \\ S_{1}^{'}\left(\lambda\right) \\ S_{2}^{'}\left(\lambda\right) \\ S_{3}^{'}\left(\lambda\right) \end{matrix}\right)=\mathbf{M}\left(\lambda\right)\cdot S\left(\lambda\right)=\left(\begin{matrix} M_{00}\left(\lambda\right) & M_{01}\left(\lambda\right) & M_{02}\left(\lambda\right) & M_{03}\left(\lambda\right) \\ M_{10}\left(\lambda\right) & M_{11}\left(\lambda\right) & M_{12}\left(\lambda_{i}\right) & M_{13}\left(\lambda\right) \\ M_{20}\left(\lambda\right) & M_{21}\left(\lambda\right) & M_{22}\left(\lambda\right) & M_{23}\left(\lambda\right) \\ M_{30}\left(\lambda\right) & M_{31}\left(\lambda\right) & M_{32}\left(\lambda\right) & M_{33}\left(\lambda\right) \end{matrix}\right)\cdot\left(\begin{matrix} S_{0}\left(\lambda\right) \\ S_{1}\left(\lambda\right) \\ S_{2}\left(\lambda\right) \\ S_{3}\left(\lambda\right) \end{matrix}\right)$$

where, $S\left(\lambda\right)$ and $S^{'}\left(\lambda\right)$ represent the incident and output Stokes parameters at different wavelengths, respectively. Since the detector capture only intensity information, the first row of the Mueller matrix is used to describe the output spectro-polarimetric intensity response. The output intensity after passing through the *i*-th meta-atom can be expressed as follows

$$\begin{matrix} I_{i}=\int_{\lambda_{1}}^{\lambda_{m}} \mathbf{M}_{0}^{i}\left(\lambda\right)\cdot S\left(\lambda\right)\text{d}\lambda+\mathbf{e} \\ =\sum_{j=0}^{3} \int_{\lambda_{1}}^{\lambda_{m}} M_{0j}^{i}\left(\lambda\right)\cdot S_{j}\left(\lambda\right)\text{d}\lambda+\mathbf{e} \end{matrix}$$

where, $m$ denotes the number of spectral channels, $M_{0}^{i}\left(\lambda\right)=\left(M_{00}^{i}(\lambda) M_{01}^{i}(\lambda) M_{02}^{i}(\lambda) M_{03}^{i}(\lambda)\right)$, and $\mathbf{e}=\left(e_{1},e_{2},...,e_{n}\right)^{\text{T}}$ represents the detection errors of each sensor unit. The spectro-polarimetric transmission equation for $n$ meta-atoms can thus be expressed as follows

$$\begin{matrix} \mathbf{I}=\mathbf{M}_{0}\cdot\mathbf{S}+\mathbf{e} \\ \left(\begin{matrix} I_{1} \\ \cdots\\ I_{n} \end{matrix}\right)=\left(\begin{matrix} \sum_{j=0}^{3} \int_{\lambda_{1}}^{\lambda_{m}} M_{0j}^{1}\left(\lambda\right)\cdot S_{j}\left(\lambda\right)\text{d}\lambda\\ \cdots\\ \sum_{j=0}^{3} \int_{\lambda_{1}}^{\lambda_{m}} M_{0j}^{n}\left(\lambda\right)\cdot S_{j}\left(\lambda\right)\text{d}\lambda\end{matrix}\right)+\mathbf{e} \end{matrix}$$

where，

$$\begin{matrix} \mathbf{S}=\left(S_{0}\left(\lambda_{1}\right),S_{1}\left(\lambda_{1}\right),S_{2}\left(\lambda_{1}\right),S_{3}\left(\lambda_{1}\right),...,S_{0}\left(\lambda_{m}\right),S_{1}\left(\lambda_{m}\right),S_{2}\left(\lambda_{m}\right),S_{3}\left(\lambda_{m}\right)\right)^{\text{T}} \\ \mathbf{M}_{0}=\left(\begin{matrix} M_{00}^{1}\left(\lambda_{1}\right) & M_{01}^{1}\left(\lambda_{1}\right) & M_{02}^{1}\left(\lambda_{1}\right) & M_{03}^{1}\left(\lambda_{1}\right) & ... & M_{00}^{1}\left(\lambda_{m}\right) & M_{01}^{1}\left(\lambda_{m}\right) & M_{02}^{1}\left(\lambda_{m}\right) & M_{03}^{1}\left(\lambda_{m}\right) \\ ... & ... & ... & ... & ... & ... & ... & ... & ... \\ M_{00}^{n}\left(\lambda_{1}\right) & M_{01}^{n}\left(\lambda_{1}\right) & M_{02}^{n}\left(\lambda_{1}\right) & M_{03}^{n}\left(\lambda_{1}\right) & ... & M_{00}^{n}\left(\lambda_{m}\right) & M_{01}^{n}\left(\lambda_{m}\right) & M_{02}^{n}\left(\lambda_{m}\right) & M_{03}^{n}\left(\lambda_{m}\right) \end{matrix}\right) \end{matrix}$$

As shown in the above equation, the spectral and polarization information can be recovered by directly pseudo-inverting the spectro-polarimetric encoding matrix $\mathbf{M}_{0}$. However, to conserve spatial resources and reduce computational and fabrication costs, the number of meta-atoms (*n*) is typically much smaller than the number of spectral channels (*m*). Therefore, accurate reconstruction in such an underdetermined framework requires $n$ highly anisotropic meta-atoms with distinct spectral and polarization responses. Each superpixel must capture sufficient information to ensure reliable recovery. In other words, each row of $\mathbf{M}_{0}$ should exhibit strong inter-row diversity to achieve effective information decoupling and high reconstruction fidelity. The explicit form of $M_{0}$ can be expressed as follows

$$M_{0}=\left(\begin{matrix} M_{00} \\ M_{01} \\ M_{02} \\ M_{03} \end{matrix}\right)^{\text{T}}=\left(\begin{matrix} \frac{1}{2}\left(T_{0}+T_{90}\right) \\ \frac{1}{2}\left(T_{0}-T_{90}\right) \\ T_{45}-\frac{1}{2}\left(T_{0}+T_{90}\right) \\ \frac{1}{2}\left(T_{0}+T_{90}\right)-T_{L} \end{matrix}\right)^{\text{T}}$$

where, $T_{0},T_{90},T_{45},T_{L}$ denote the transmittances for 0°, 90°, 45°, and left-handed circularly polarized (LCP) light, respectively.

**Supplementary Note 2: Design of metasurface structure and dataset generation.**

To realize metasurface unit cells with anisotropic properties to different spectral and polarization states, two distinct metasurface structures are designed, as shown in Figure S1. The nanopillars are composed of titanium dioxide (TiO_2_) on a silicon dioxide (SiO_2_) substrate. Both structures adopt diatomic nanopillar patterns, where in-plane symmetry breaking modulates their optical responses. The distinct spectral and polarization characteristics arise from the complex scattering and coupling of Bloch modes within the metasurface layer [1-2]. Specifically, the relative arrangement of nanopillars and precise tuning of geometric parameters enable sensitivity to various polarization states. The introduction of in-plane symmetry breaking allows the unit cells to exhibit different spectral behaviors under different polarization conditions, thereby expanding the functional design space. Moreover, by adjusting the nanostructures geometry, the spectral and polarization responses can be flexibly tailored, enabling the realization of metasurface units with unique customized optical functionalities.

Finite-difference time-domain (FDTD) simulations were conducted for the two proposed metasurface structures. Each structure was analyzed under four linear polarization states (0°, 45°, 90°, and 135°) as well as left- and right-handed circularly polarized (LCP and RCP) incident light to obtain their spectral responses. The geometrical parameters of the both structures were systematically scanned, while the period and nanopillar height were fixed at 500 nm and 600 nm, respectively. The spectral were sampled at 501 wavelength points. A minimum spacing of 100 nm between adjacent meta-atoms was ensured to avoid near-field coupling [3-4]. In total, a dataset comprising 2,900 samples under different polarization conditions was generated for each structure, with geometrical parameter dimensions of 2,900×8 and spectro-polarimetric data dimensions of 2,900×501×6. Figure S2 and Figure S3 show the spectral response curves of the two structures under four different sets of geometrical parameters and various polarization states, demonstrating rich spectral diversity.


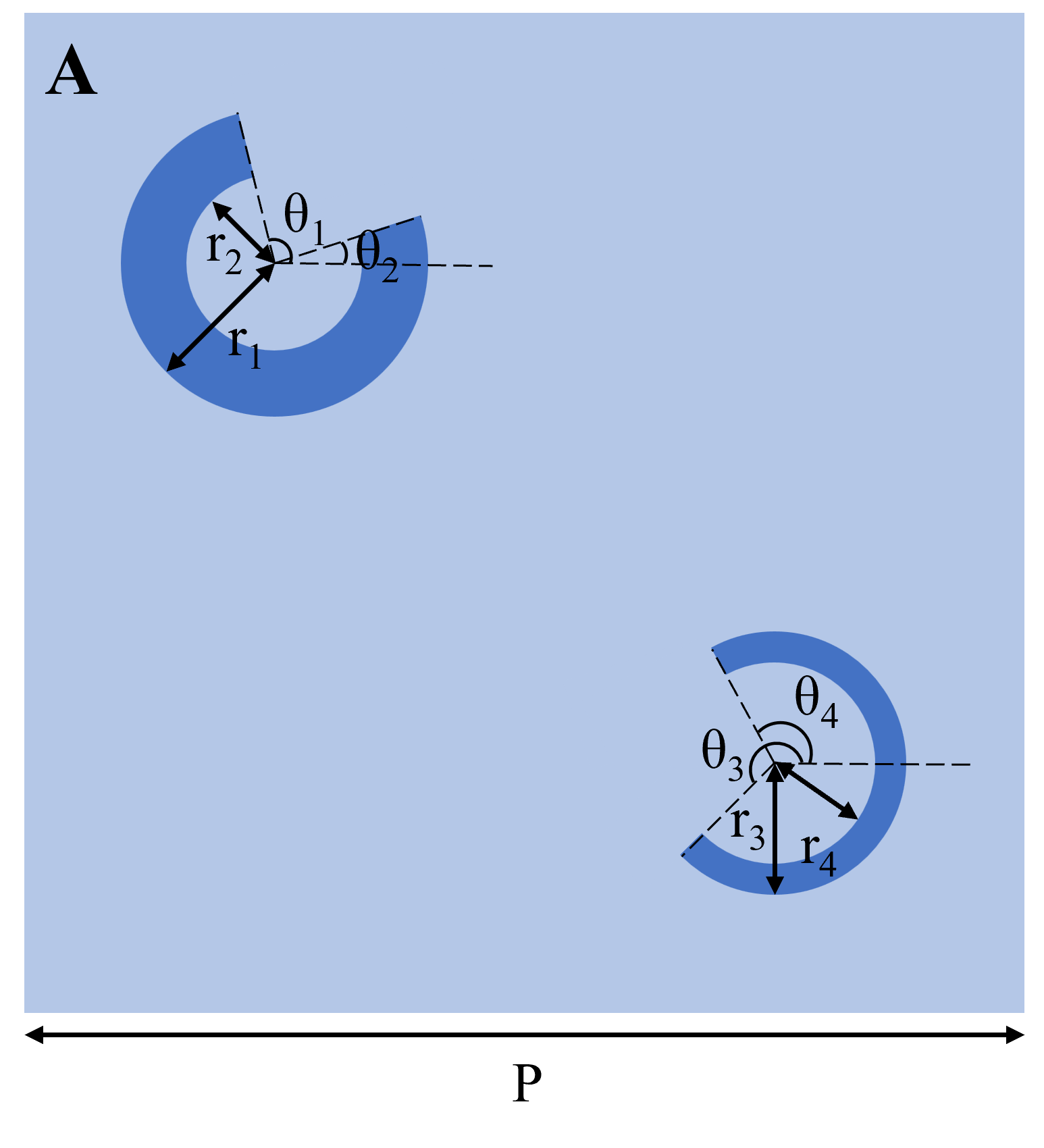

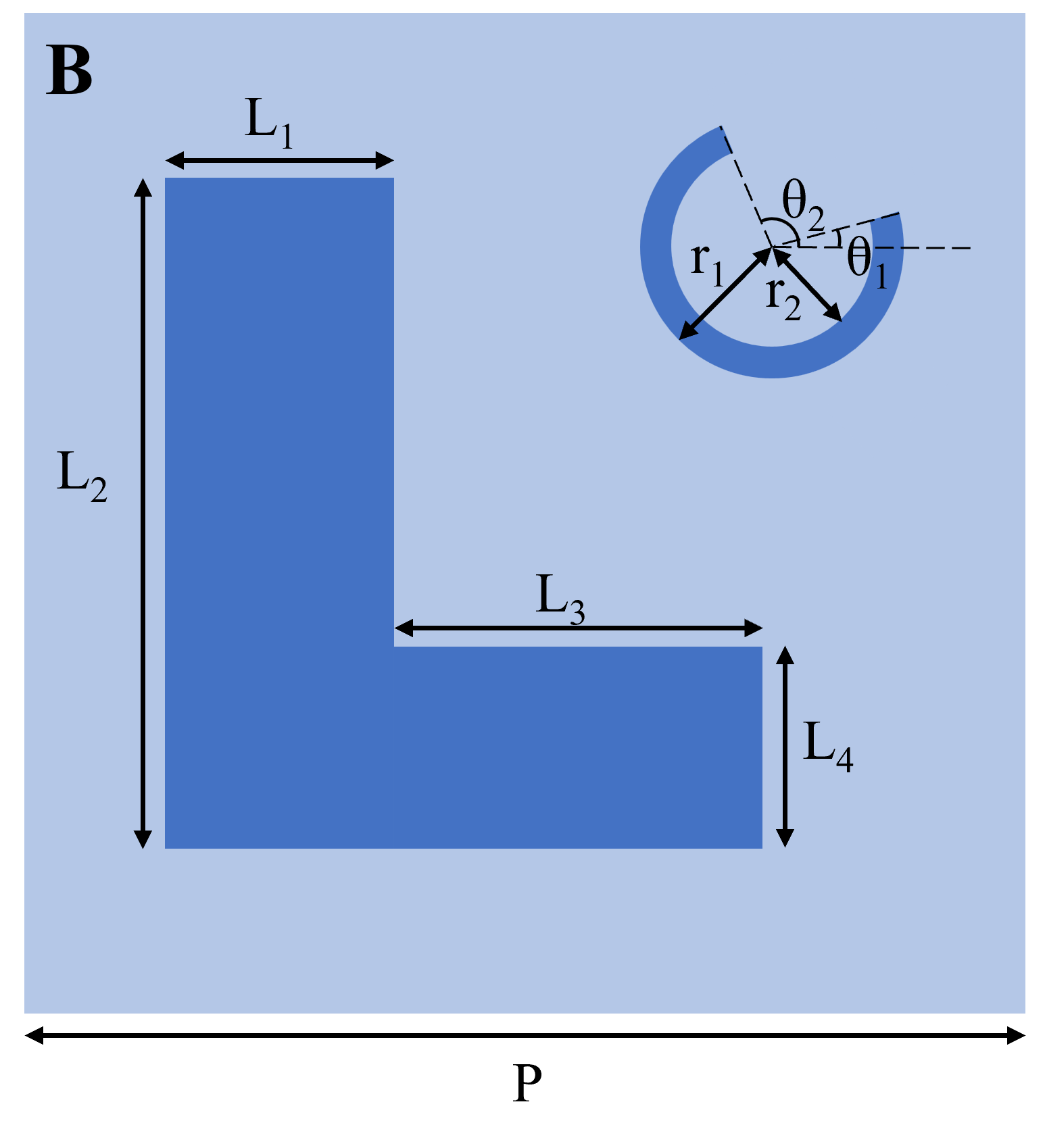


Figure S1. Schematic of the metasurface unit cell and definition of geometric parameters.


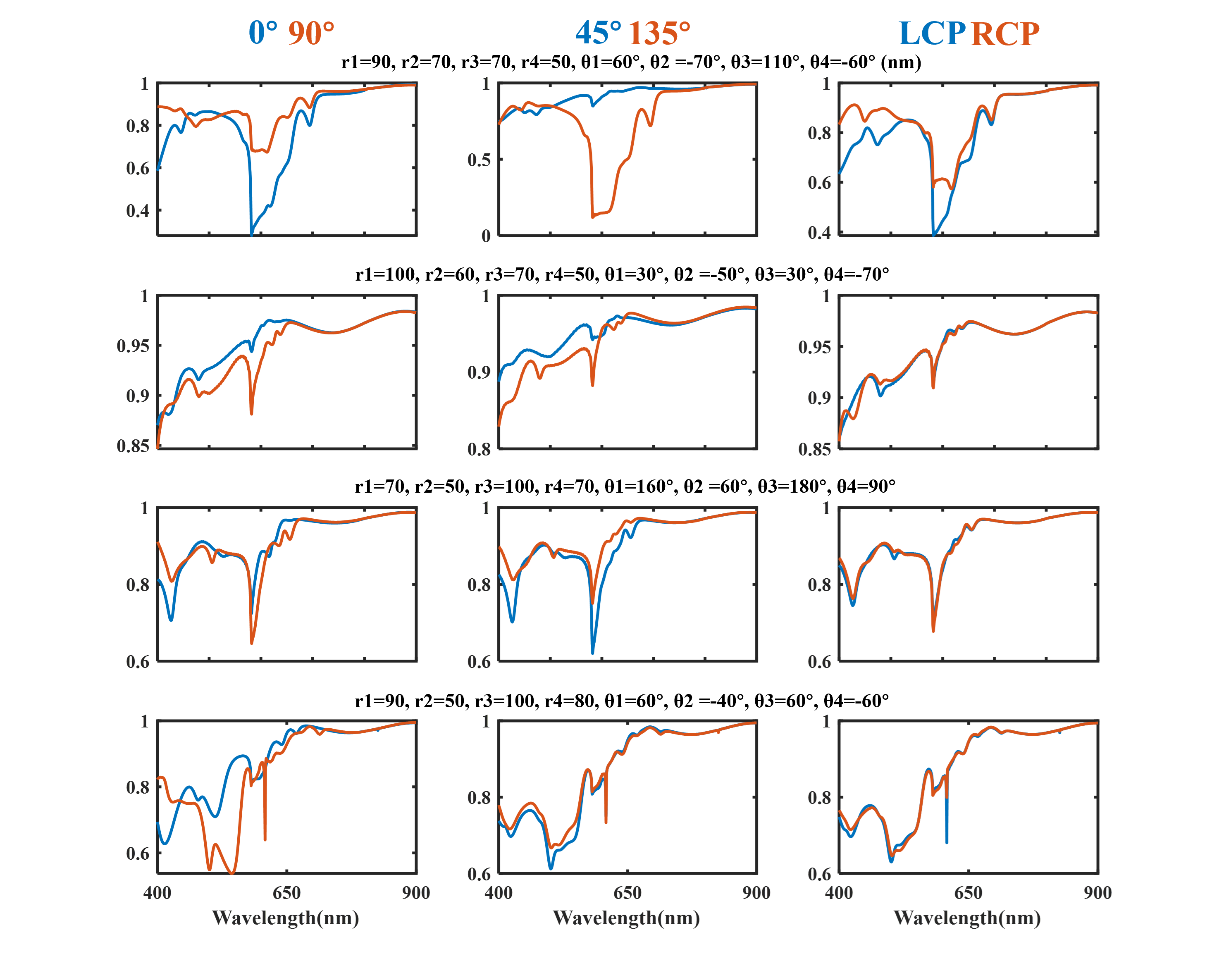


Figure S2. Spectral response curves of Structure A for various geometric parameters and polarization states.


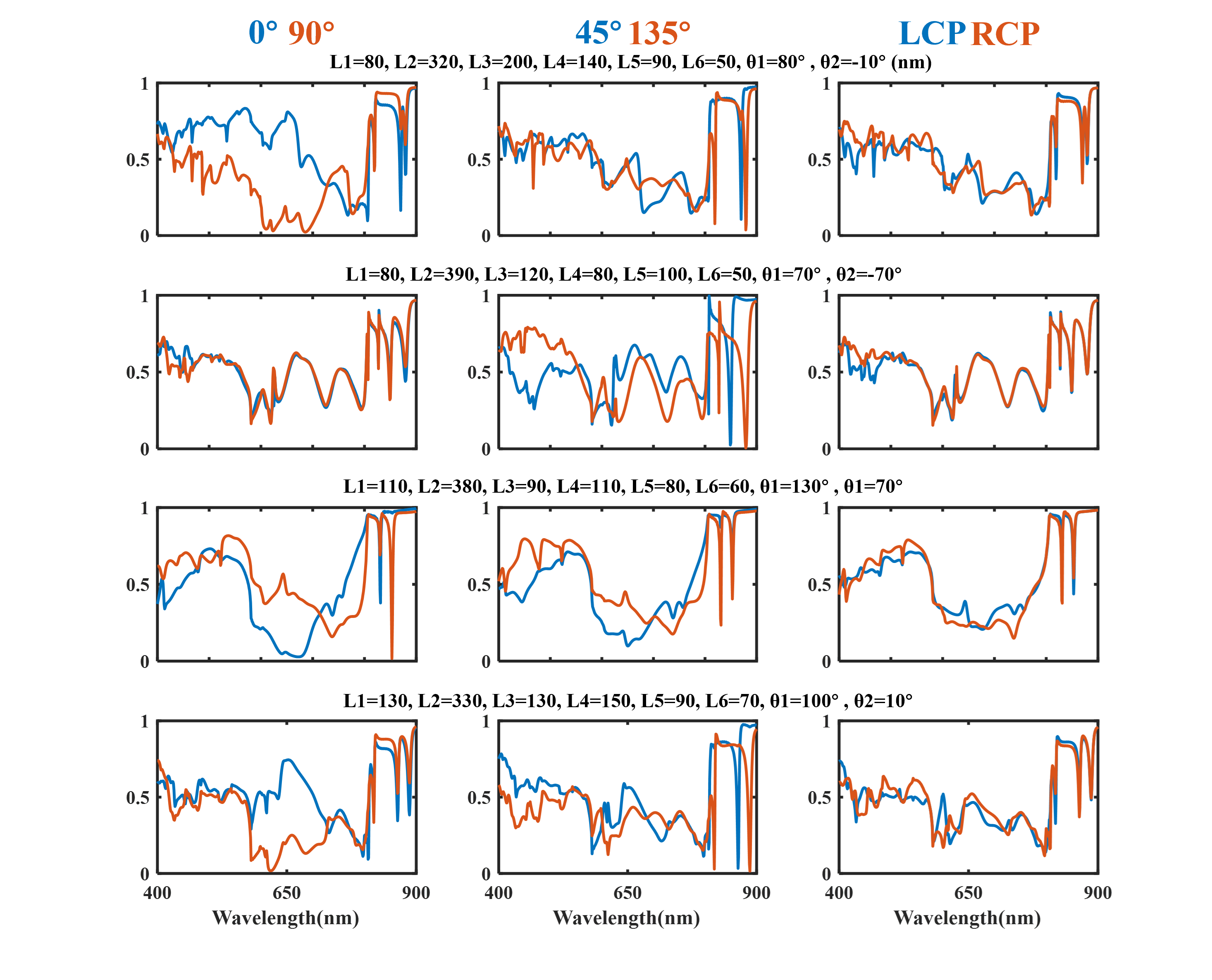


Figure S3. Spectral response curves of Structure B for various geometric parameters and polarization states.

**Supplementary Note 3: Analysis of the electric field distribution in the metasurface structure.**

We further employed the finite-difference time-domain (FDTD) method to numerically simulate the electric-field distributions of the two metasurface structures under different polarization states. The simulation results are shown in Figure S4. The electric-field distributions of the two structures in the transverse plane (x-y plane) are presented in Figure S4(a) and (c). As can be observed, under different incident polarization states, pronounced variations in local field enhancement occur at the edges, openings, and inter-element regions of the split-ring and L-shaped nanostructures. These variations indicate the selective excitation of localized resonant modes under different polarization conditions and underscore the strong modulation of the electromagnetic field induced by the geometric asymmetry of the structures. The corresponding electric-field distributions in the longitudinal plane (x-z plane) are shown in Figure S4(b) and (d). Under different polarization states, the electric-field within and around the metasurface layer exhibits distinct propagation behaviors. These differences include variations in propagation direction, energy distribution, and field attenuation characteristics.

These phenomena mainly arise from the scattering, interference, and coupling of Bloch modes in the periodic metasurface structures [1-2]. Because the two designs employ different mechanisms of in-plane symmetry breaking, the supported Bloch mode branches and their coupling conditions are highly sensitive to the incident polarization. As a result, distinctly different local field distributions and propagation behaviors are observed under different polarization states. These electric-field distribution results further confirm the diversity and complementarity of the spectro-polarimetric responses of the proposed metasurface structures.


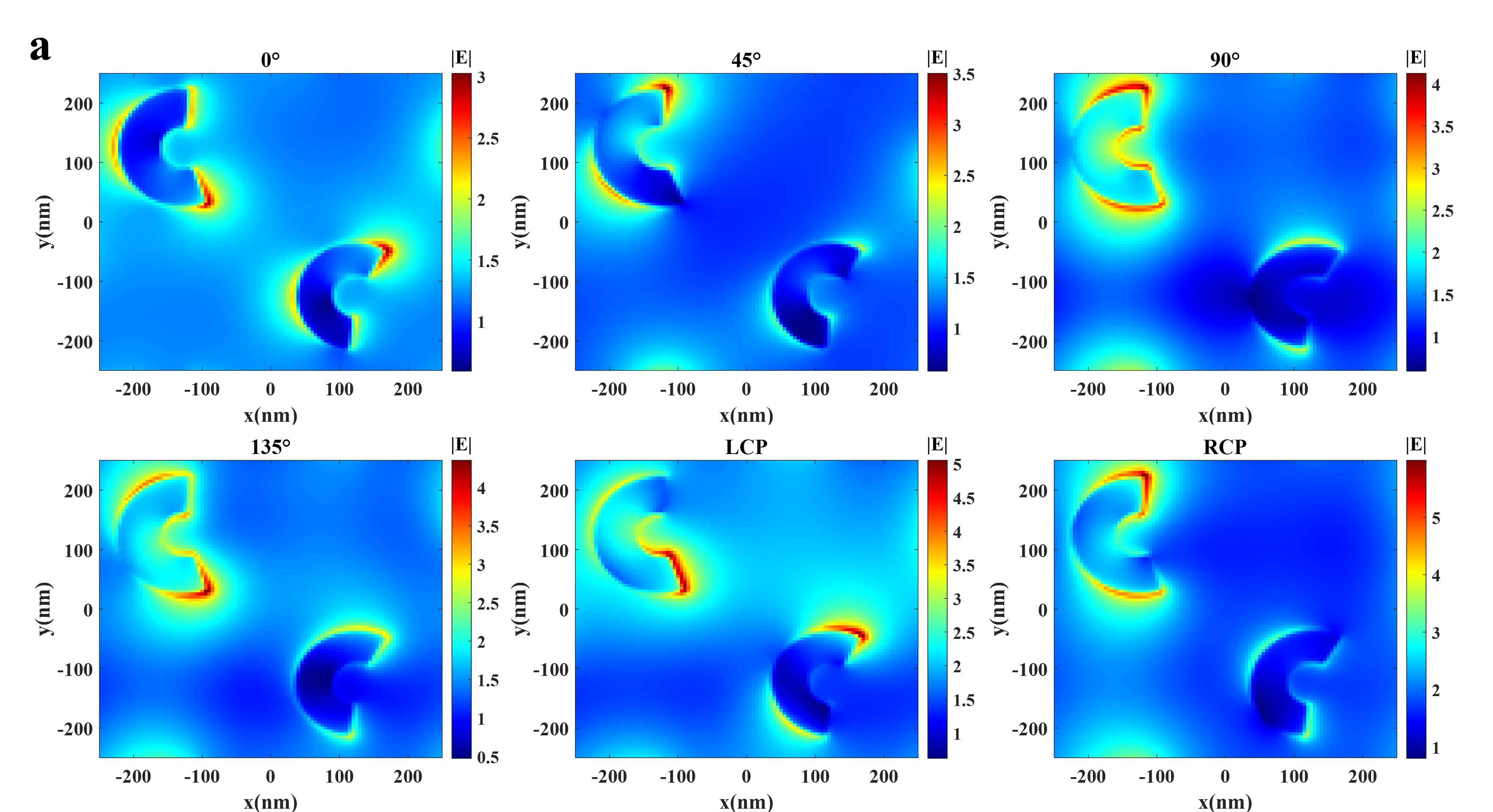

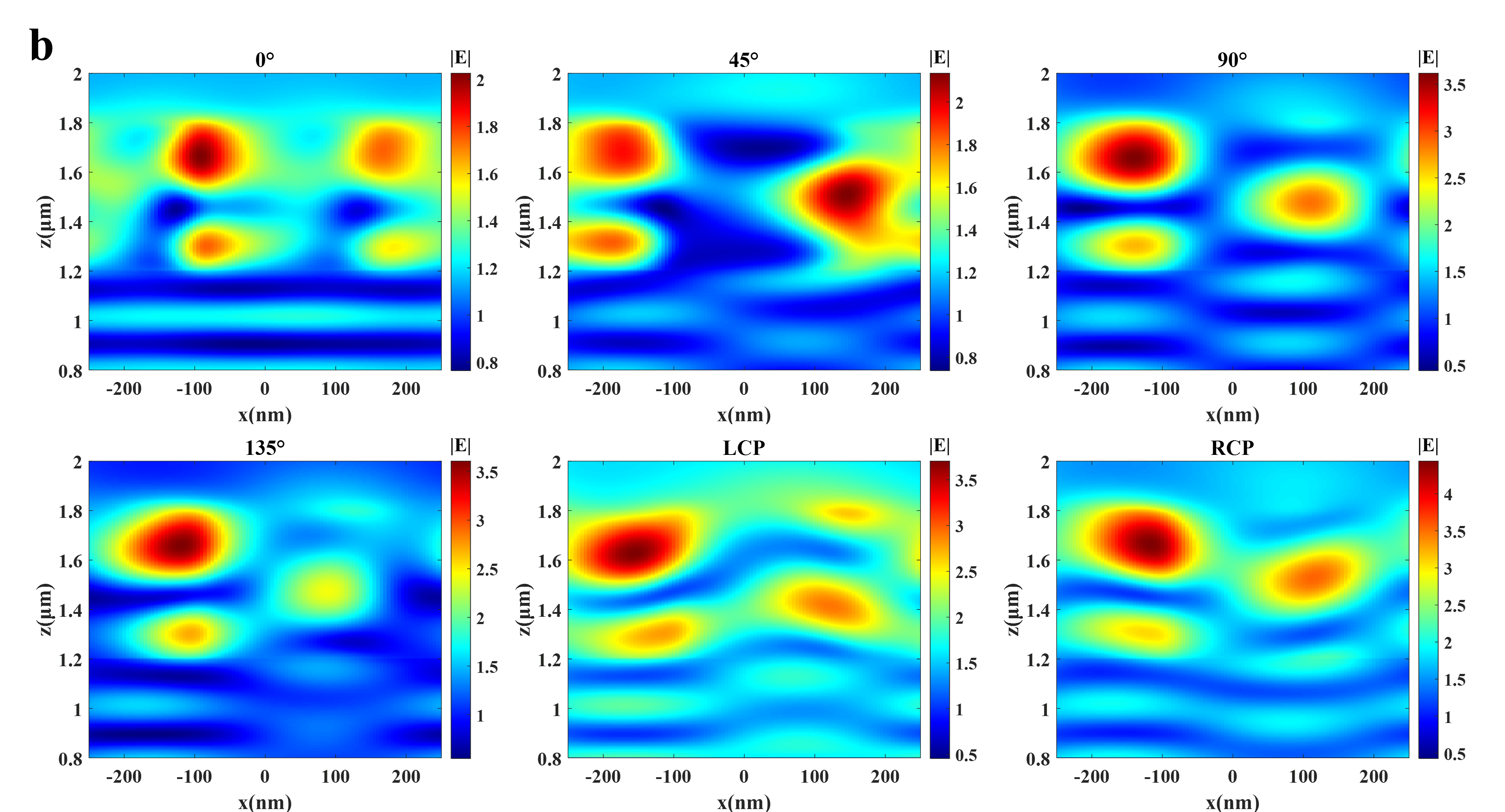

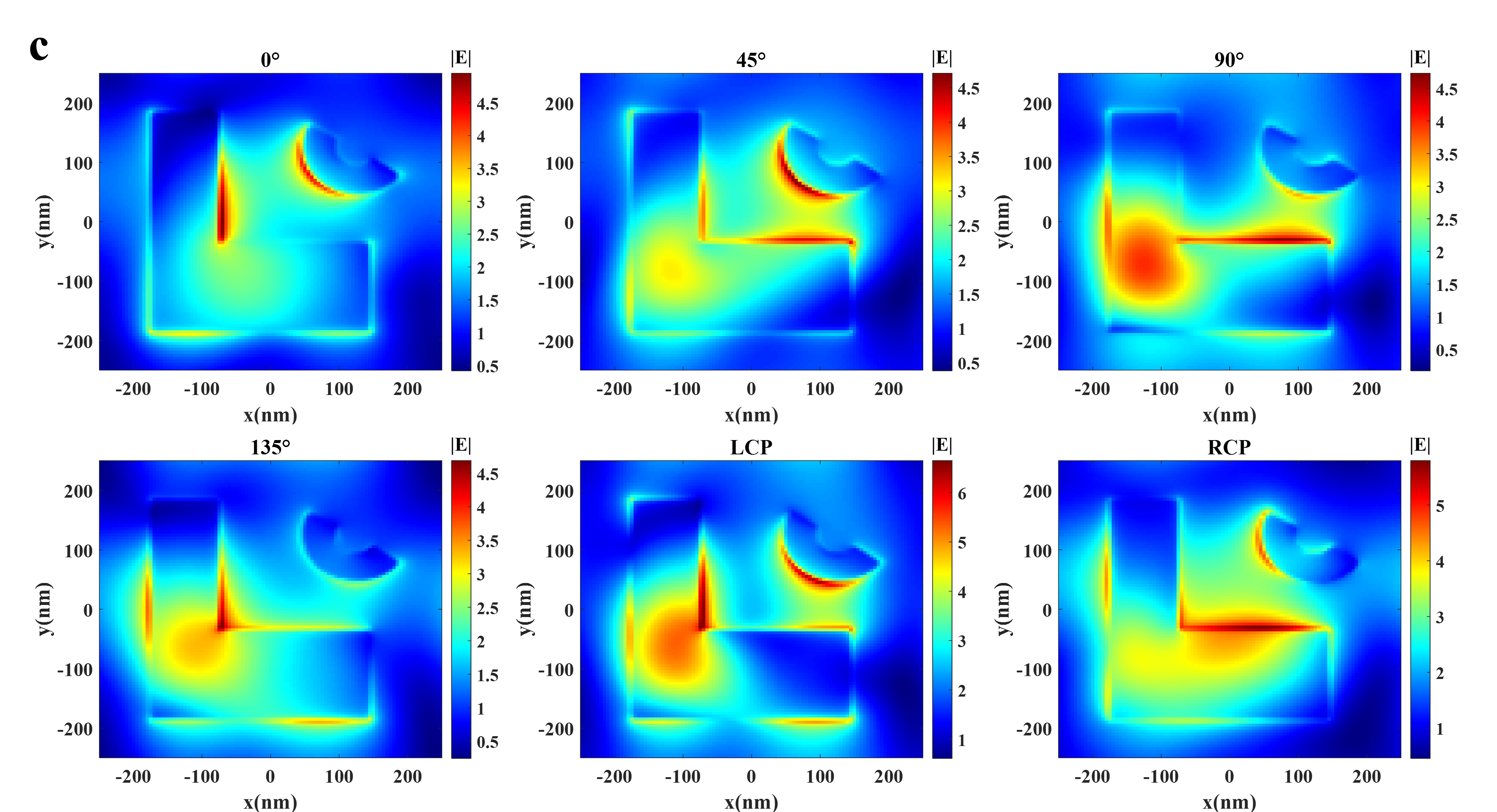

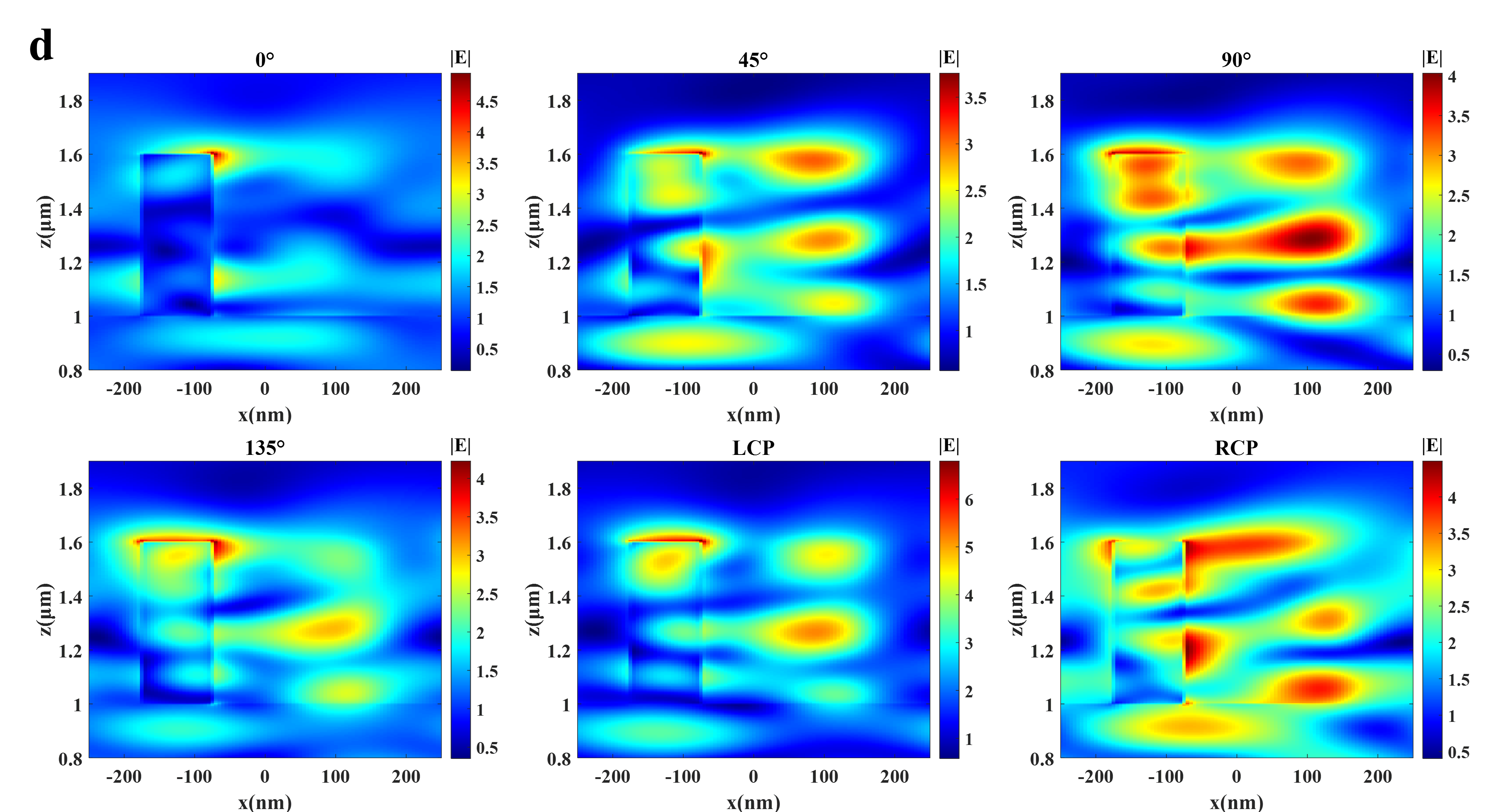


Figure S4. Electric field distribution in the x-y plane and x-z plane.

**Supplementary Note 4: Analysis of latent vector.**

This study investigates two distinct metasurface structures and aims to predict their transmittance and geometric parameters using a neural network model. To effectively differentiate between the two structures, latent vectors **Z**1 and **Z**2 were introduced in addition to the input geometric parameters. These latent vectors were sampled from two normal distributions with different means and standard deviations, thereby maximizing the separation between the two groups. The detailed parameter settings are provided in Table S1, and the corresponding distribution curves are shown in Figure S5. As illustrated, the two distributions remain clearly separated even with a dataset size of 10,000. Incorporating these latent vectors enables the neural network to effectively identify different metasurface types during training, improving both robustness and generalization capability.

Table S1. Parameters of the normal distributions for the latent vectors.

|  | Mean | Standard Deviation |
| --- | --- | --- |
| Structure A (**Z**1) | 0.1 | 0.1 |
| Structure B (**Z**2) | 0.9 | 0.1 |

Figure S5. Normal distribution of the latent vectors.

The geometric parameters of the two metasurface types have a dimensionality of 8. Since the dimension of the latent vectors can affect the model’s optimization, it is necessary to determine an optimal dimension. Four latent vector dimensions 4, 8, 12, and 16 were tested. Each latent vector was concatenated with the geometric parameters and input into the forward network for training. The Forward_Loss between the predicted spectral data and the ground truth was then calculated for the test set, and the spectral responses under different polarization states were analyzed. The results of this analysis are shown in Figure S6 and Figure S7.

Figure S6 presents box plots of prediction errors for different latent vector dimensions, illustrating the statistical characteristics of predictions across various polarization states. The results indicate that the latent vector dimension significantly influences the error distribution. For latent vector dimensions of 8, 12, and 16, the box plots show more outliers and longer boxes for each polarization direction, reflecting a wider error distribution and increased model sensitivity to noise. In contrast, the 4-dimensional latent vector exhibits the fewest outliers and relatively compact boxes, resulting in more stable predictions.

Figure S7 further presents the mean and standard deviation of prediction errors across different polarization directions for various latent vector dimensions. The bar charts show that, across all polarization states, latent vectors with 8, 12, and 16 dimensions generally produce higher mean errors than the 4-dimensional latent vector, particularly for 0°, 45°, 90°, and 135° polarizations. The line plots of standard deviations illustrate the variation of errors for each latent vector dimension. The 4-dimensional latent vector exhibits the smallest standard deviation across all polarization directions, indicating more stable and consistent predictions that are robust against noise.

Based on the analysis above, a latent vector dimension of 4 demonstrates stronger robustness and lower prediction errors. A smaller dimension effectively mitigates both overfitting and underfitting, allowing the model to provide more accurate and reliable predictions with fewer parameters. In contrast, increasing the latent vector dimension reduces the relative importance of the geometric parameters. The model tends to rely excessively on the latent vector to capture data complexity, neglecting critical geometric features and diminishing their contribution to predictions. Therefore, a 4-dimensional latent vector maintains high stability and low variance across multiple polarization states, enhancing both the generalization capability and prediction accuracy.


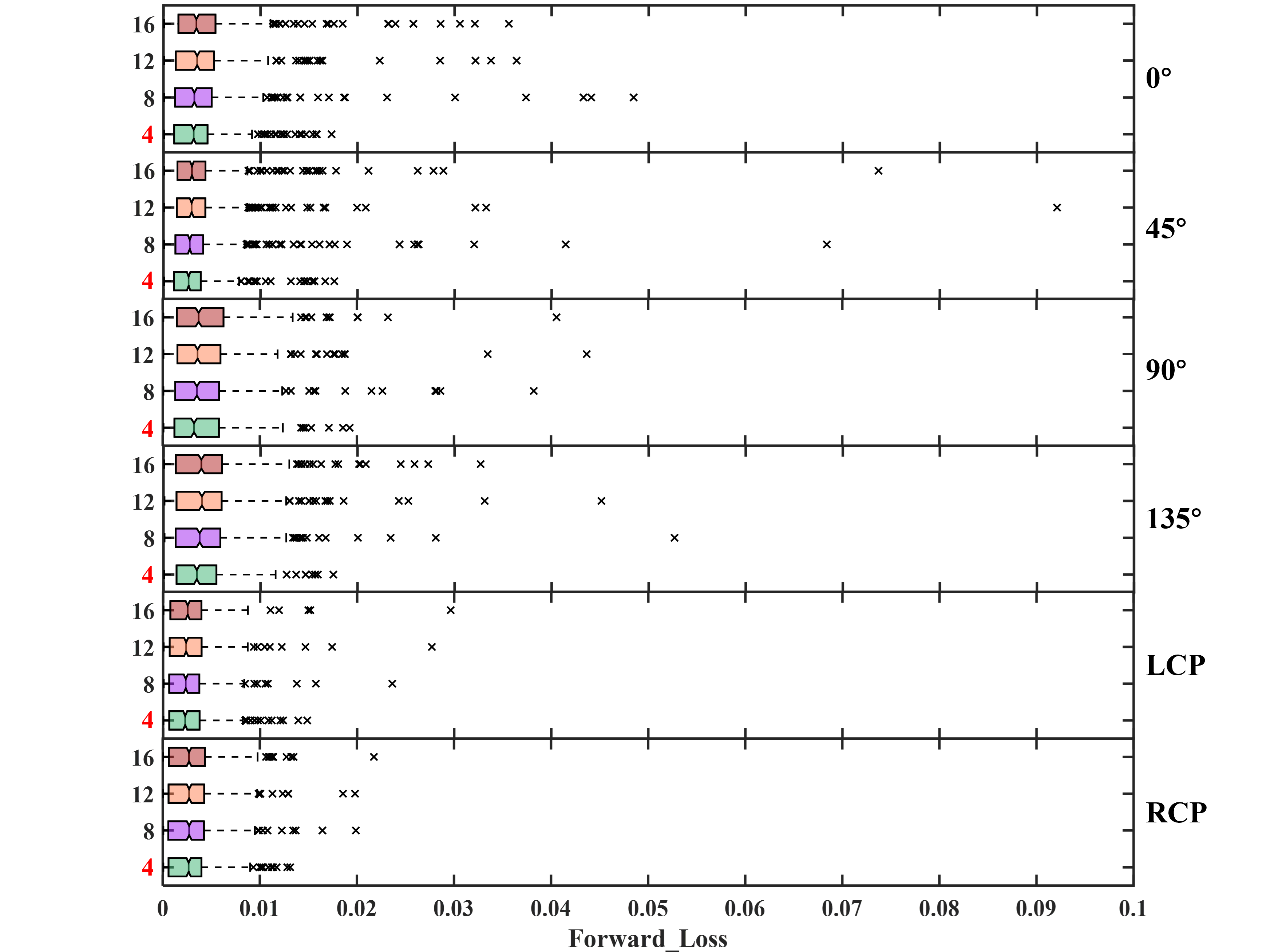


Figure S6. Box-plot depicting the loss of forward network on the test set.


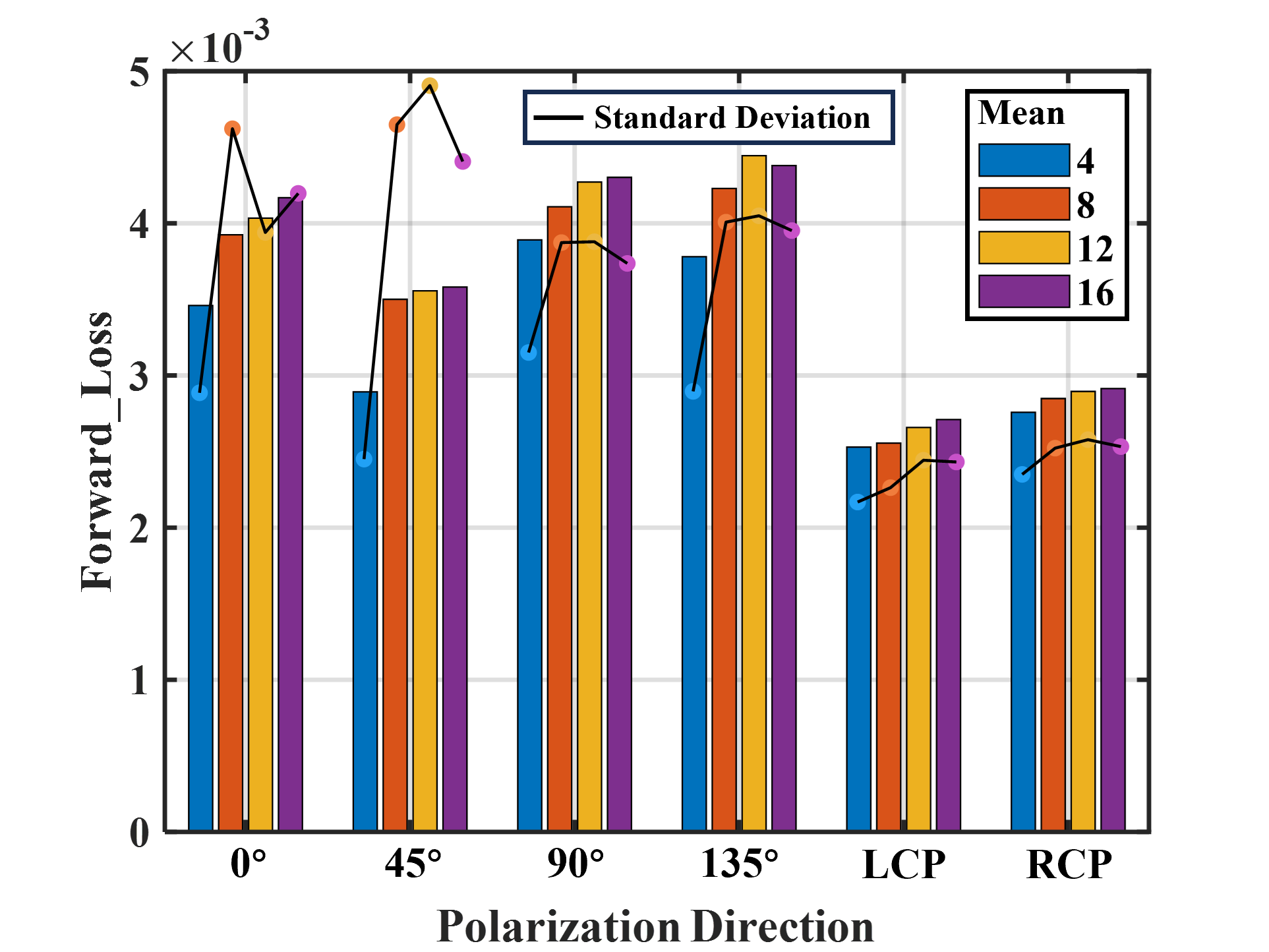


Figure S7. Statistical data depicting the loss of forward network on the test set.

**Supplementary Note 5: AE network training.**

Figure S8 illustrates the architecture of the autoencoder (AE) network. Both the encoder and decoder adopt a symmetric structure, each consisting of an input layer, an output layer, and three fully connected layers. The encoder has neurons layers of sizes 800, 600, and 400, ultimately mapping the input data into a 200-dimensional latent space. The decoder symmetrically reconstructs the data, ensuring effective compression and accurate recovery.

In processing the dataset, the spectral data from the two structures were first merged to form a dataset of size 5800×501×6. The spectral data for all six polarization states were then flattened and integrated into a 34800×501 dataset, which was randomly shuffled. This shuffling enhances training robustness and improves the model’s generalization ability. The preprocessing approach not only improves the model’s adaptability to diverse input data but also mitigates potential biases caused by data ordering, thereby enhancing the stability and accuracy of prediction and optimization tasks. The AE network was trained using the Adam optimizer for 4000 epochs with a mean squared error (MSE) loss constraint. The learning rate was set to $5\times10^{-3}$.

Figure S9(a) shows the training and validation loss curves. Both losses steadily decreased within the first 100 epochs and gradually converge to $1.2\times10^{-4}$ as the learning rate decreased. Figure S9(b-g) present the prediction results of the AE network, demonstrating that the predicted values closely match the simulated values. This confirms the network’s ability to accurately reconstruct the data. By using the AE network for dimensionality reduction, key features can be effectively extracted while redundant information is removed. As a result, computational complexity is reduced, and the model’s generalization ability is enhanced. This enables more accurate capture of the intrinsic structure of the output data, making subsequent prediction and optimization processes more robust.


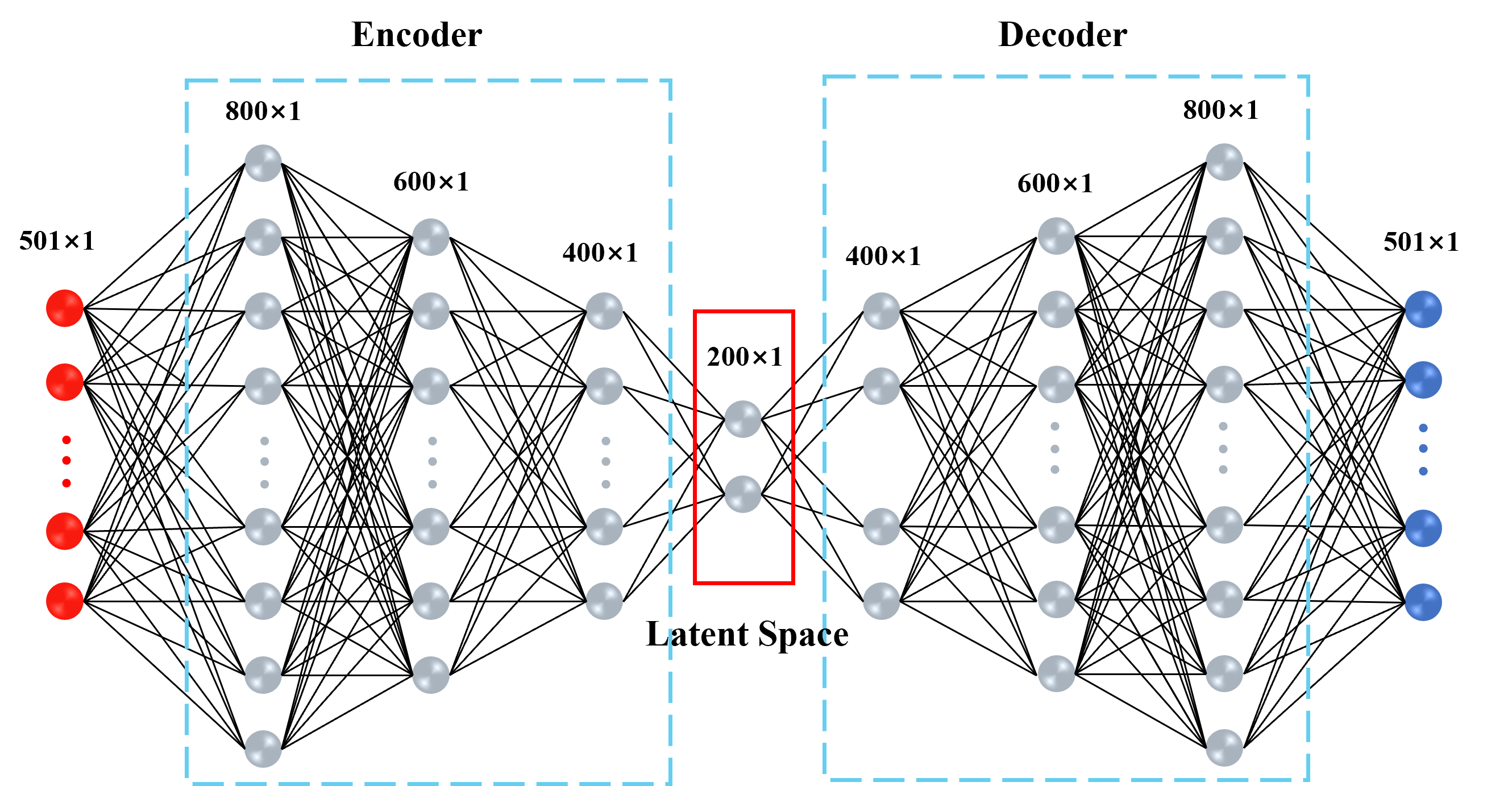


Figure S8. The schematic of AE network


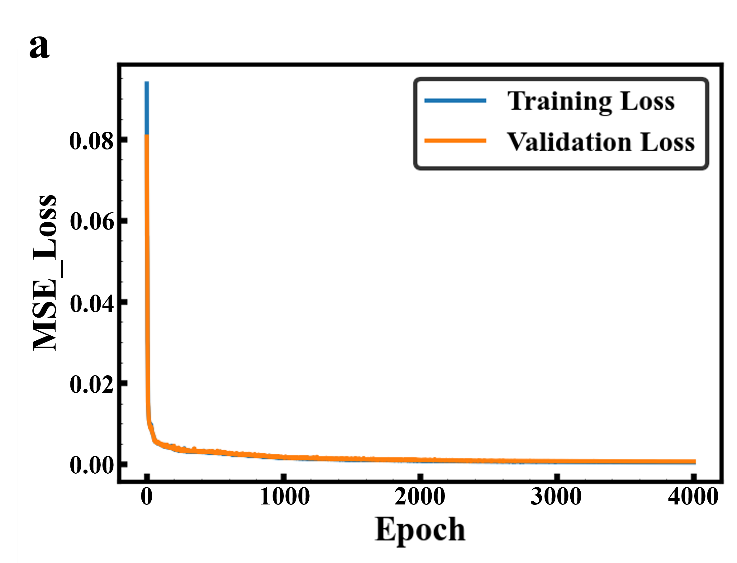


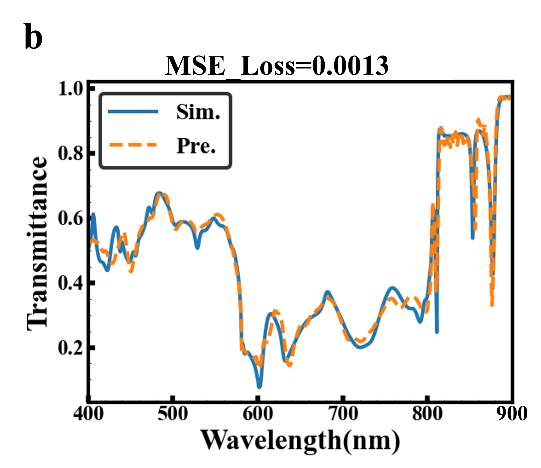

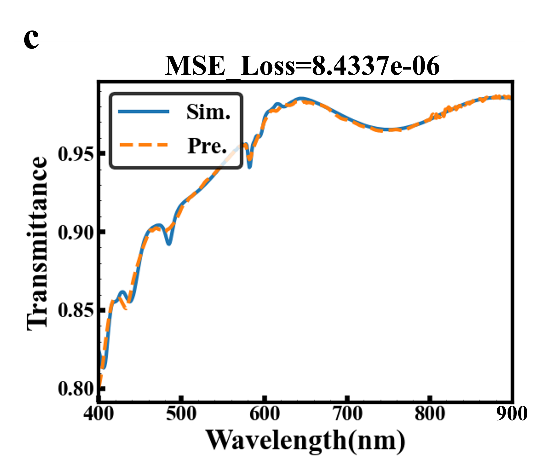

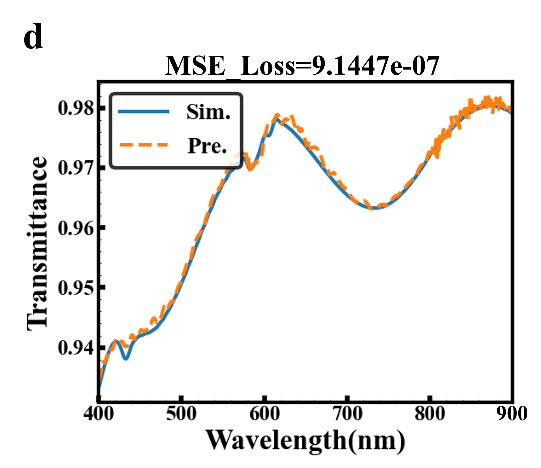

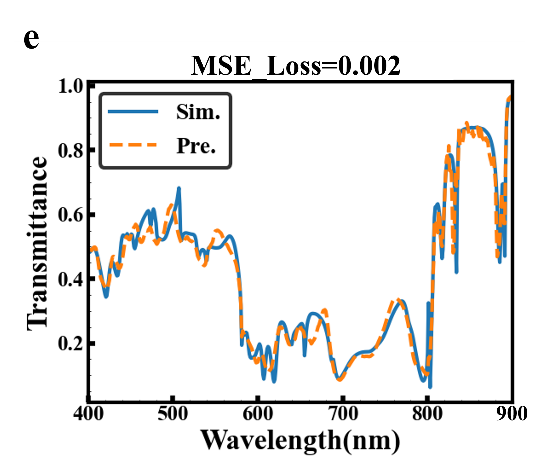

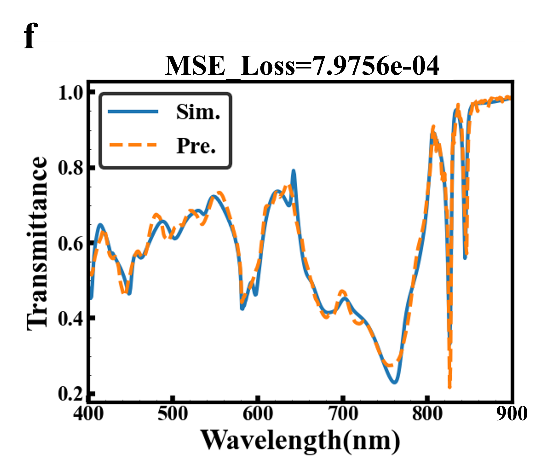

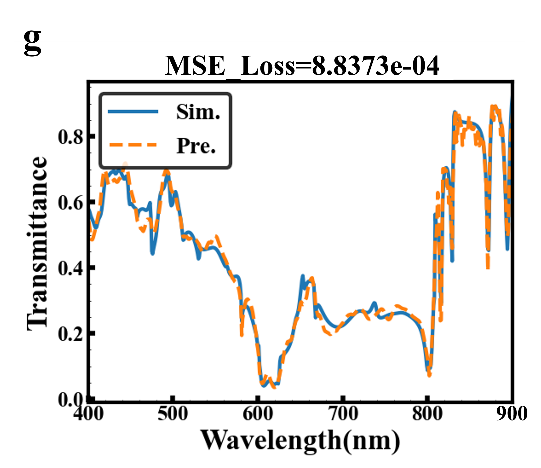


Figure S9. The training results of AE network. (a) The training and validation loss curves. (b-g) The prediction results of AE network.

**Supplementary Note 6: Forward network training.**

In the forward network architecture, a structure consisting of one shared hidden layer and six parallel subnetworks was designed. The shared hidden layer takes the 12-dimensional geometric parameters, including a 4-dimensional latent vector, as input and maps them into six sets of 64-dimensional feature representations. Each feature set is then fed into a subnetwork with an identical structure, producing six outputs of 200 dimensions each. These outputs are finally upscaled through the decoder of the pre-trained AE network [5], generating six sets of 501-dimensional transmittance data.

During the optimization process, an L2 regularizer was applied to penalize the activations of all hidden layers, encouraging the network to learn sparse features and internal representations of the original observations [6]. Table S2 lists the training parameters. The forward network was trained using the Adam optimizer for 600 epochs. The batch size was set to 128 to accelerate training. The learning rate was initially set as 5×10^-3^ in the first 350 epoch, then linearly decayed to 0, making the network converge smoothly.

Table S2. The training hyperparameters of forward network.

| **Hyperparameter** | **Value** |
| --- | --- |
| Epoch | 600 |
| Learning rate | $5\times10^{-3}\times\left[1-\text{Max}\left(0,\text{ }\frac{epoch-350}{350}\right)\right]$ |
| Optimizer | Adam |
| Batch size | 128 |
| Number of tasks | 6 |

Figure S10 presents the training and validation loss curves. Both losses decreased sharply during the first 50 epochs and then fluctuated around 0.0025 until 250th epoch, indicating that the network performance had saturated at this learning rate. Finally, the losses gradually converge to 0.001 as the learning rate decreased. Figure S11 shows the full-Stokes spectro-polarimetric responses of the two metasurface structures under different geometric parameter conditions. The results indicate that, for both metasurface types, the forward network can accurately predict the spectral responses under varying polarization states. This demonstrates the network’s strong data discrimination capability, high prediction accuracy, and robustness.


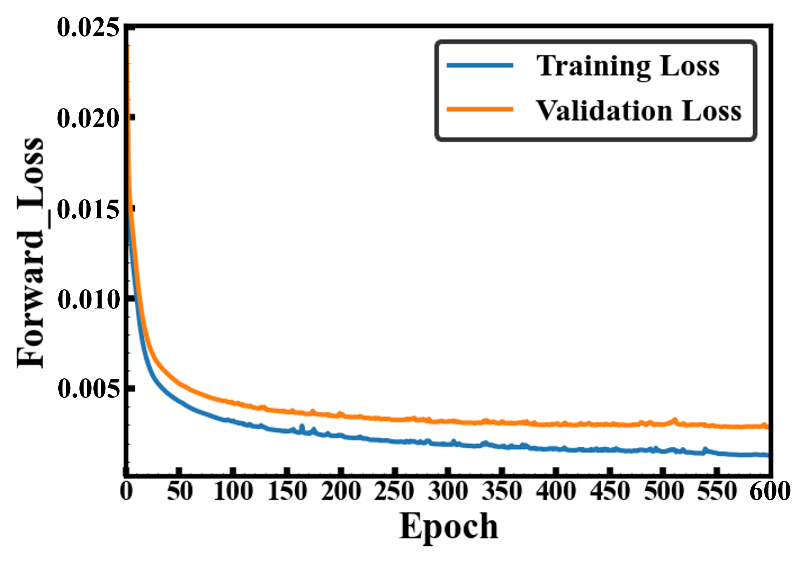


Figure S10. The training and validation loss curves of forward network.


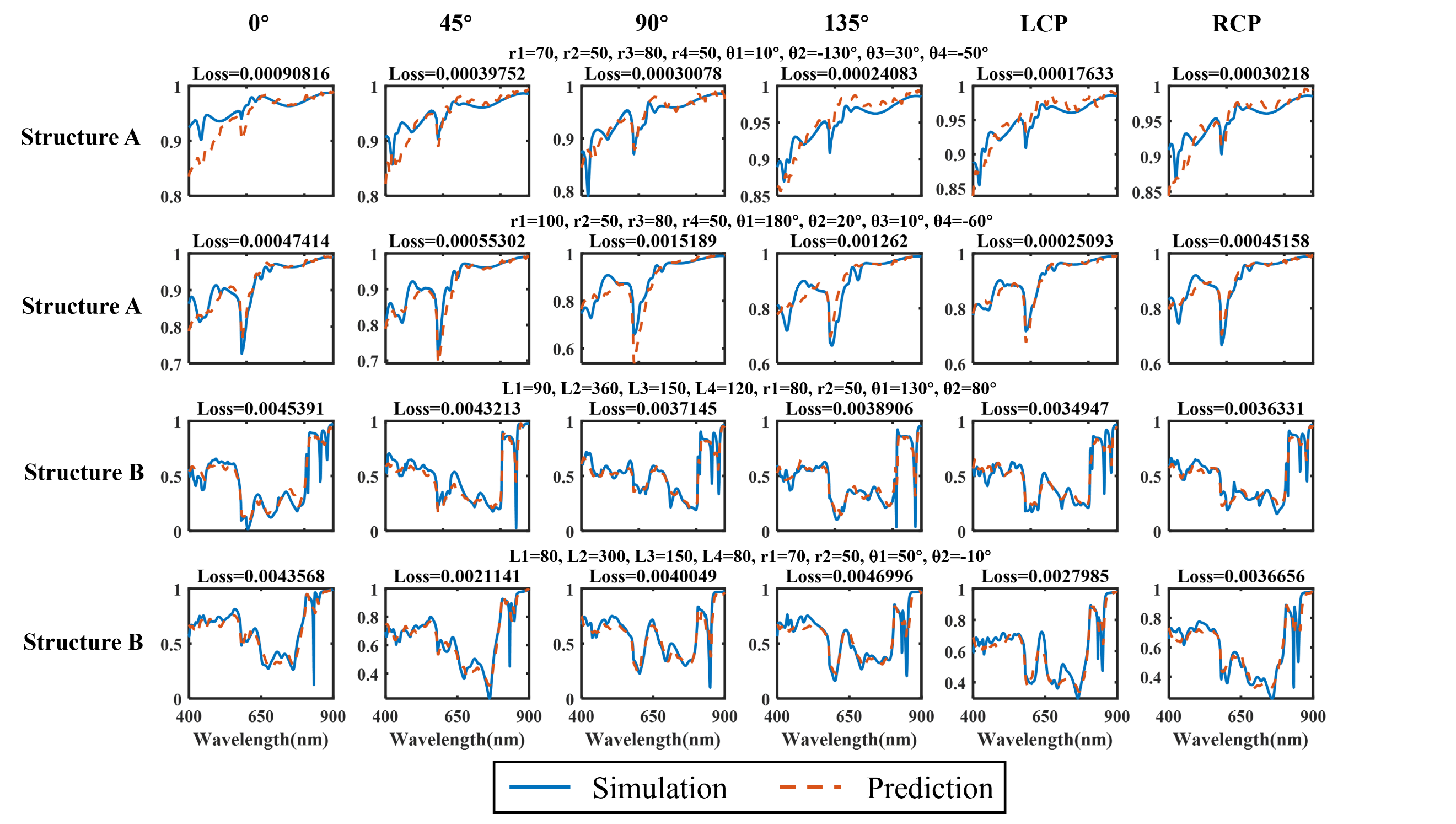


Figure S11. The training results for forward network under multiple geometric parameters.

**Supplementary Note 7: Inverse network dataset.**

A total of 10,000 samples from the established $\text{C}_{5800}^{2}$ group inverse network data were selected to form the final dataset for training the inverse design network. The table below presents five randomly chosen samples from these 10,000, where A and B correspond to the two metasurface types proposed in this work. The dataset spans a wide range of correlation coefficients, from highly correlated to weakly correlated. This ensures that the network can learn the complex mappings between different structural parameters. Comparing prediction performance across correlation levels allows further evaluation of the model’s inverse design capability and generalization under varying degrees of structural association.

Table S3. Inverse design dataset with different correlation coefficients (units: nm).

| Group 1 (C=0.13) | | | | | | | | |
| --- | --- | --- | --- | --- | --- | --- | --- | --- |
| (A) para1 | 70 | 20 | 75 | 40 | 0 | -80 | 20 | -120 |
| (B) para2_1 | 100 | 400 | 150 | 80 | 76 | 20 | 30 | -100 |
| (B) para2_2 | 110 | 370 | 170 | 80 | 88 | 30 | 30 | -140 |
| (B) para2_3 | 90 | 380 | 190 | 140 | 88 | 30 | 170 | 70 |
| (B) para2_4 | 110 | 310 | 80 | 80 | 92 | 20 | 110 | -40 |
| Group 2 (C=0.24) | | | | | | | | |
| (B) para1 | 100 | 310 | 160 | 150 | 100 | 40 | 150 | 20 |
| (A) para2_1 | 95 | 30 | 75 | 20 | 60 | 60 | 70 | -70 |
| (A) para2_2 | 85 | 50 | 75 | 40 | 80 | -60 | 50 | -60 |
| (A) para2_3 | 100 | 50 | 85 | 40 | 30 | -100 | 0 | 0 |
| (A) para2_4 | 90 | 30 | 100 | 60 | 80 | 40 | 130 | 100 |
| Group 3 (C=0.32) | | | | | | | | |
| (A) para1 | 75 | 30 | 80 | 60 | 180 | 110 | 80 | -80 |
| (A) para2_1 | 70 | 40 | 80 | 60 | 170 | 120 | 50 | 0 |
| (A) para2_2 | 85 | 40 | 95 | 60 | 50 | -100 | 80 | -60 |
| (A) para2_3 | 95 | 30 | 95 | 20 | 100 | 30 | 80 | -10 |
| (A) para2_4 | 70 | 50 | 80 | 30 | 90 | 50 | 10 | -110 |
| Group 4 (C=0.46) | | | | | | | | |
| (B) para1 | 90 | 370 | 200 | 110 | 98 | 20 | 170 | 30 |
| (B) para2_1 | 130 | 370 | 170 | 150 | 84 | 50 | 50 | 0 |
| (B) para2_2 | 150 | 390 | 90 | 120 | 76 | 50 | 20 | -100 |
| (B) para2_3 | 140 | 310 | 90 | 110 | 82 | 30 | 50 | -40 |
| (B) para2_4 | 110 | 330 | 150 | 100 | 86 | 60 | 50 | -20 |
| Group 5 (C=0.50) | | | | | | | | |
| (A) para1 | 85 | 50 | 80 | 30 | 70 | -40 | 120 | 20 |
| (A) para2_1 | 75 | 50 | 100 | 50 | 60 | -110 | 130 | -40 |
| (A) para2_2 | 90 | 60 | 90 | 50 | 0 | -160 | 0 | -180 |
| (A) para2_3 | 80 | 30 | 100 | 80 | 40 | -40 | 0 | -80 |
| (A) para2_4 | 100 | 70 | 85 | 20 | 70 | -50 | 90 | -20 |

**Supplementary Note 8: Inverse network training.**

In the inverse design network, both geometric parameters and correlation coefficients are used as network inputs. A multitask learning framework is employed to enable the network to output multiple sets of parameters corresponding to the given geometric parameters and correlation coefficients. Furthermore, during training, the inverse network is combined with the forward network to form a composite conditional multitask tandem architecture. This design ensures high stability and efficient optimization performance, effectively addresses the “one-to-many” problem in inverse design. The training parameters of the inverse network are listed in Table S4.

Figure S12 presents the training and validation loss curves of the sequential network. As shown, the inverse loss decreases rapidly during the first 50 epochs and then gradually stabilizes, reaching a minimum value of approximately 0.0025 around the 500th epoch. Although minor fluctuations are observed within the first 500 epochs, the overall trend remains downward, indicating that the model continues to learn and converge effectively. Moreover, the small gap between the training and validation losses suggests that the model exhibits good generalization performance, with no significant overfitting observed.

After training, the test dataset was input into the inverse network to obtain the predicted results. Figure S13 compares four sets of simulated and predicted geometric parameters under four different correlation coefficient conditions, where A and B represent the two metasurface types proposed in this work. Based on these parameters, FDTD simulations were performed to calculate the transmittance under various polarization states, as shown in Figure S14. The results demonstrate that the transmittance predicted by the inverse network closely matches the true values across all polarization directions, showing excellent overall waveform consistency. These findings confirm that the proposed deep learning model possesses strong capability for joint spectral and polarization modeling and enables on-demand design of spectro-polarimetric encoding metasurface, offering an effective approach for the efficient inverse design of multidimensional optical devices.

Table S4. The training hyperparameters of Inverse network.

| **Hyperparameter** | **Value** |
| --- | --- |
| Epoch | 500 |
| Learning rate | $1\times10^{-3}\times\left[1-\text{Max}\left(0,\text{ }\frac{epoch-300}{300}\right)\right]$ |
| Optimizer | Adam |
| Batch size | 256 |
| Number of tasks | 4 |
| $\eta_{1}$ | 0.3 |
| $\eta_{2}$ | 0.7 |


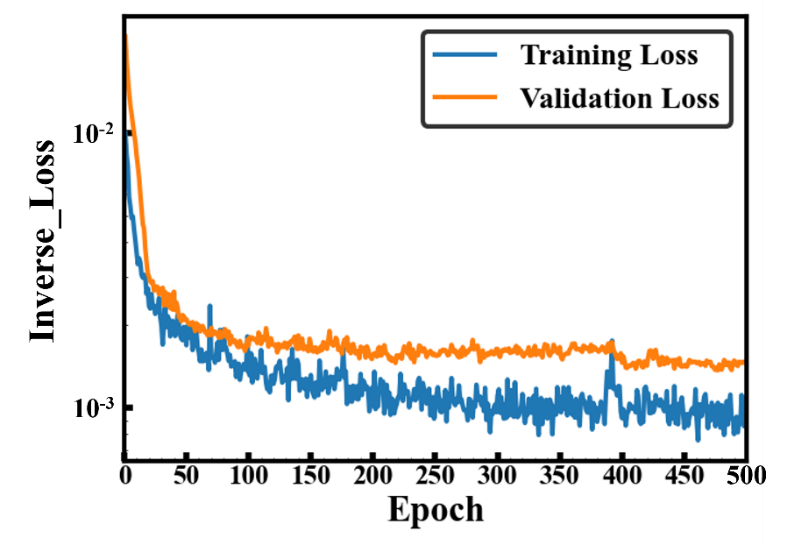


Figure S12. The training and validation loss curves of tandem network.


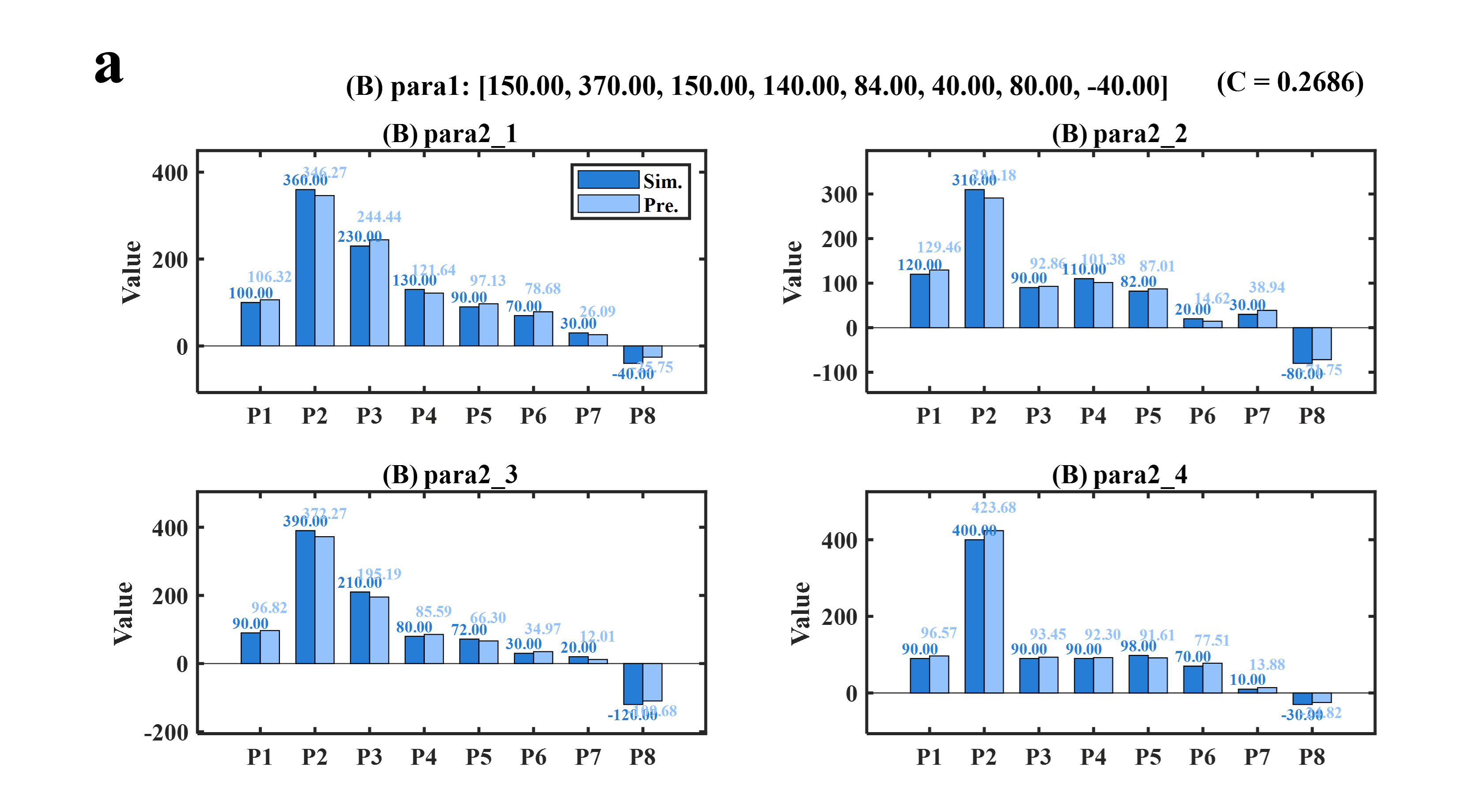

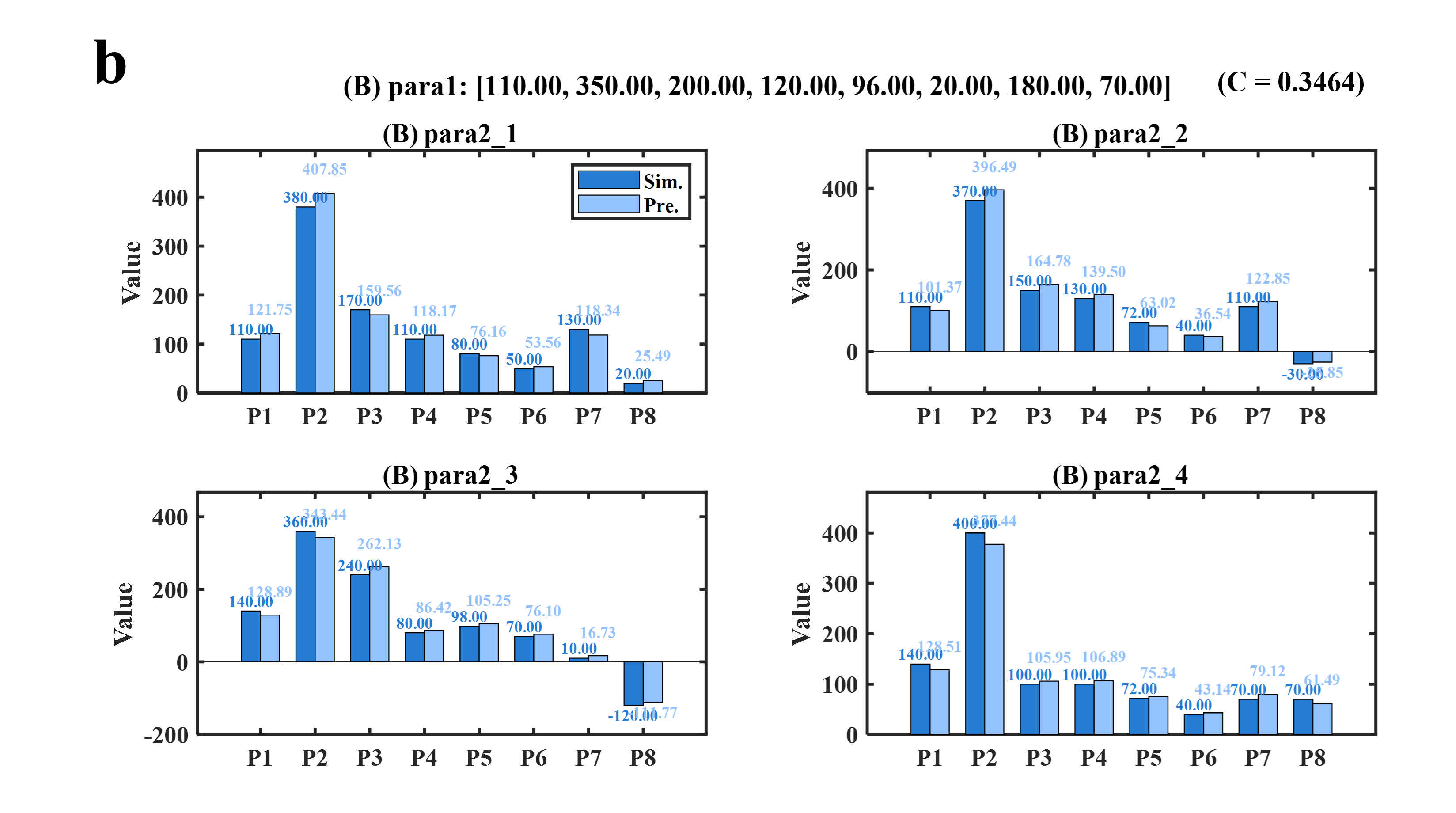

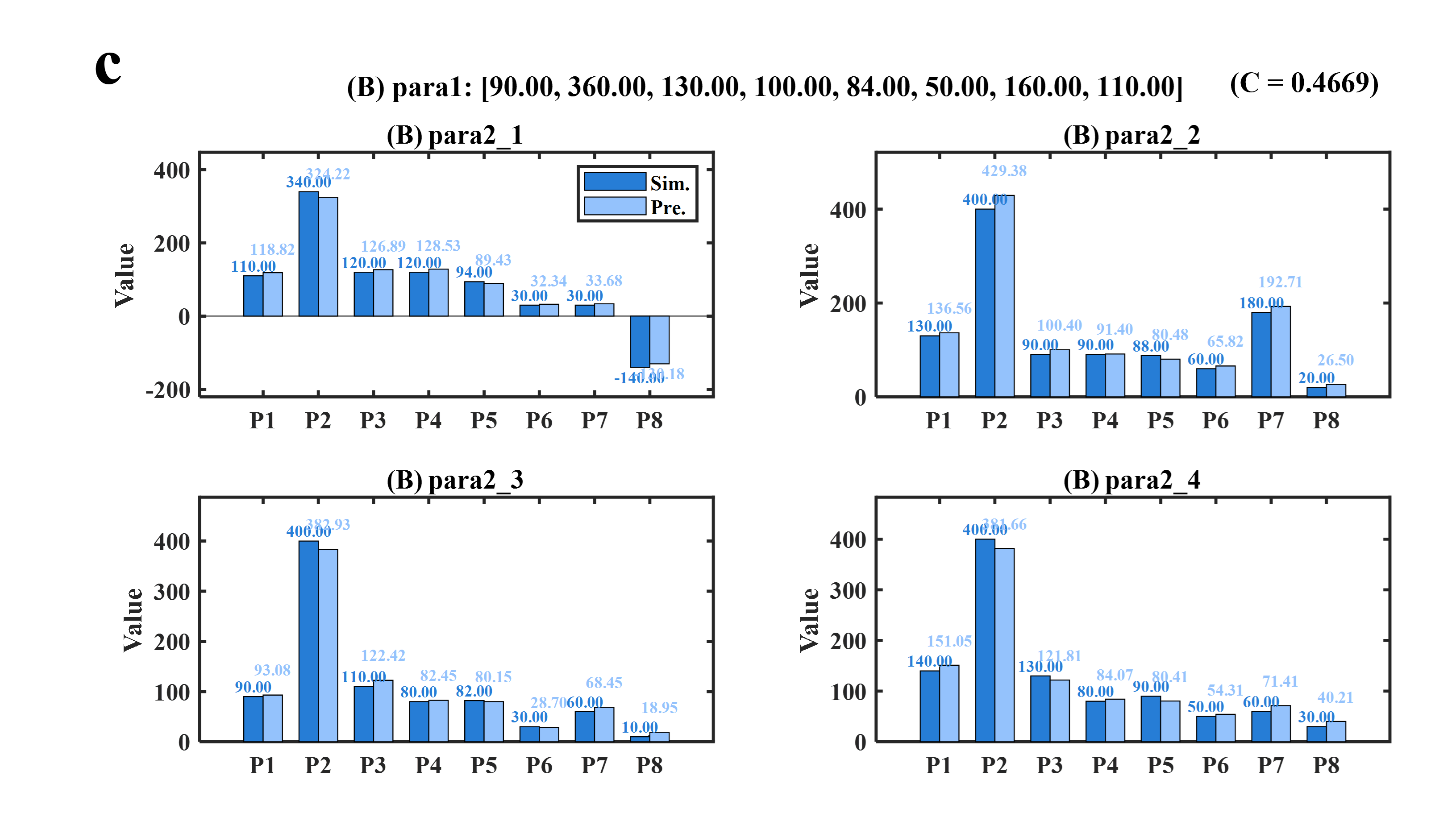

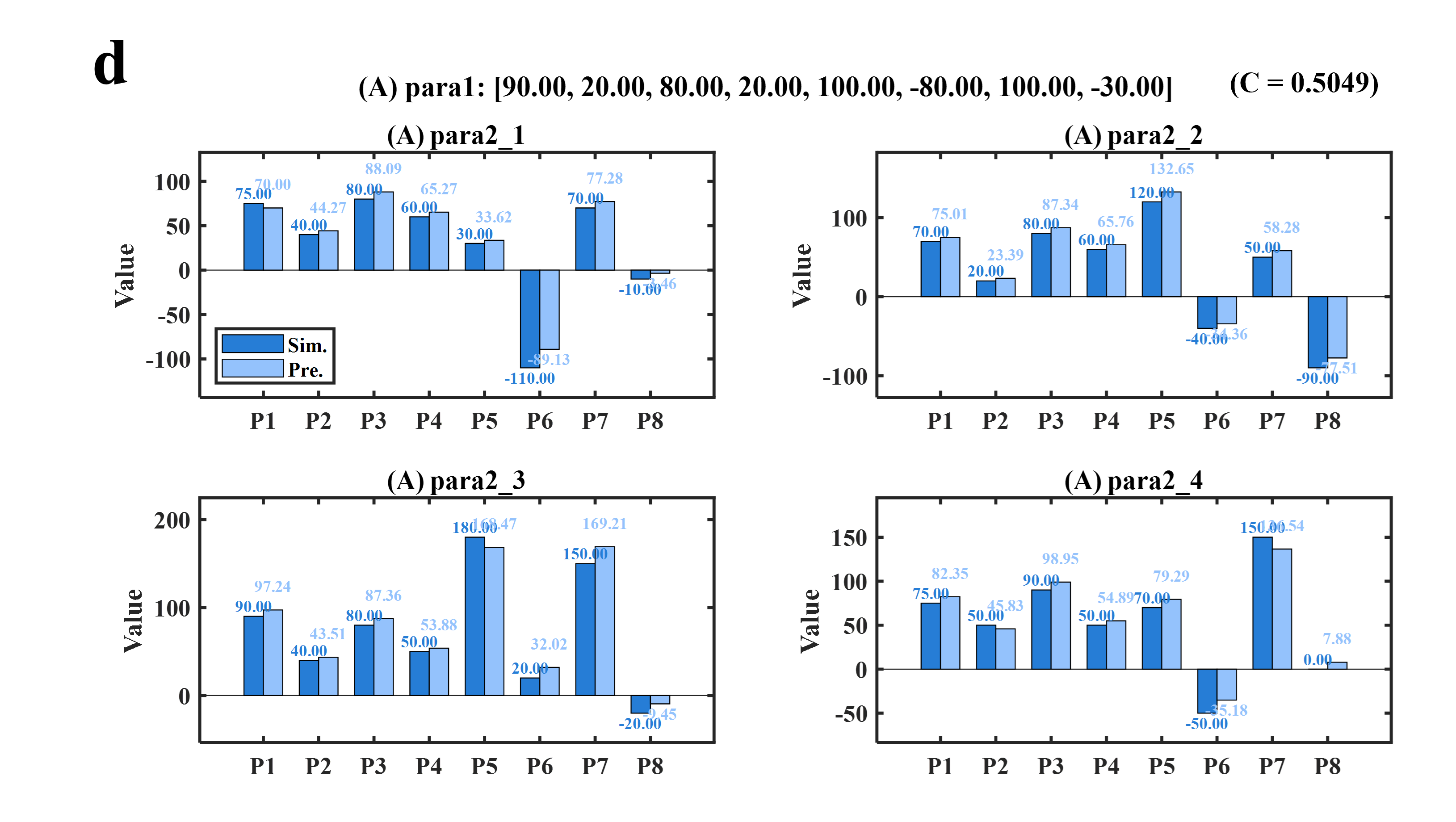


Figure S13. Comparison between simulated and predicted results obtained from the inverse design network (unit: nm). A and B represent the two types of metasurface structures.


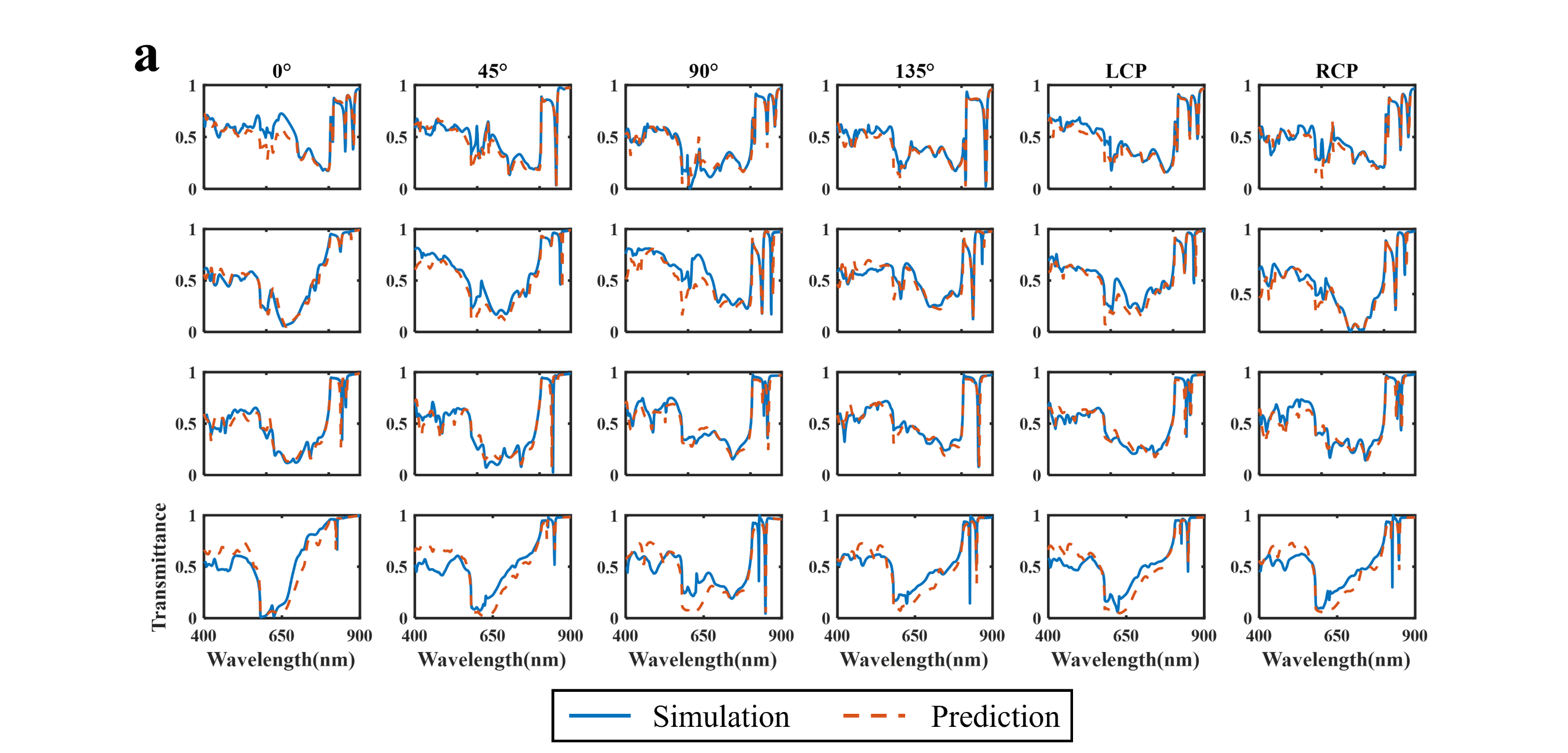

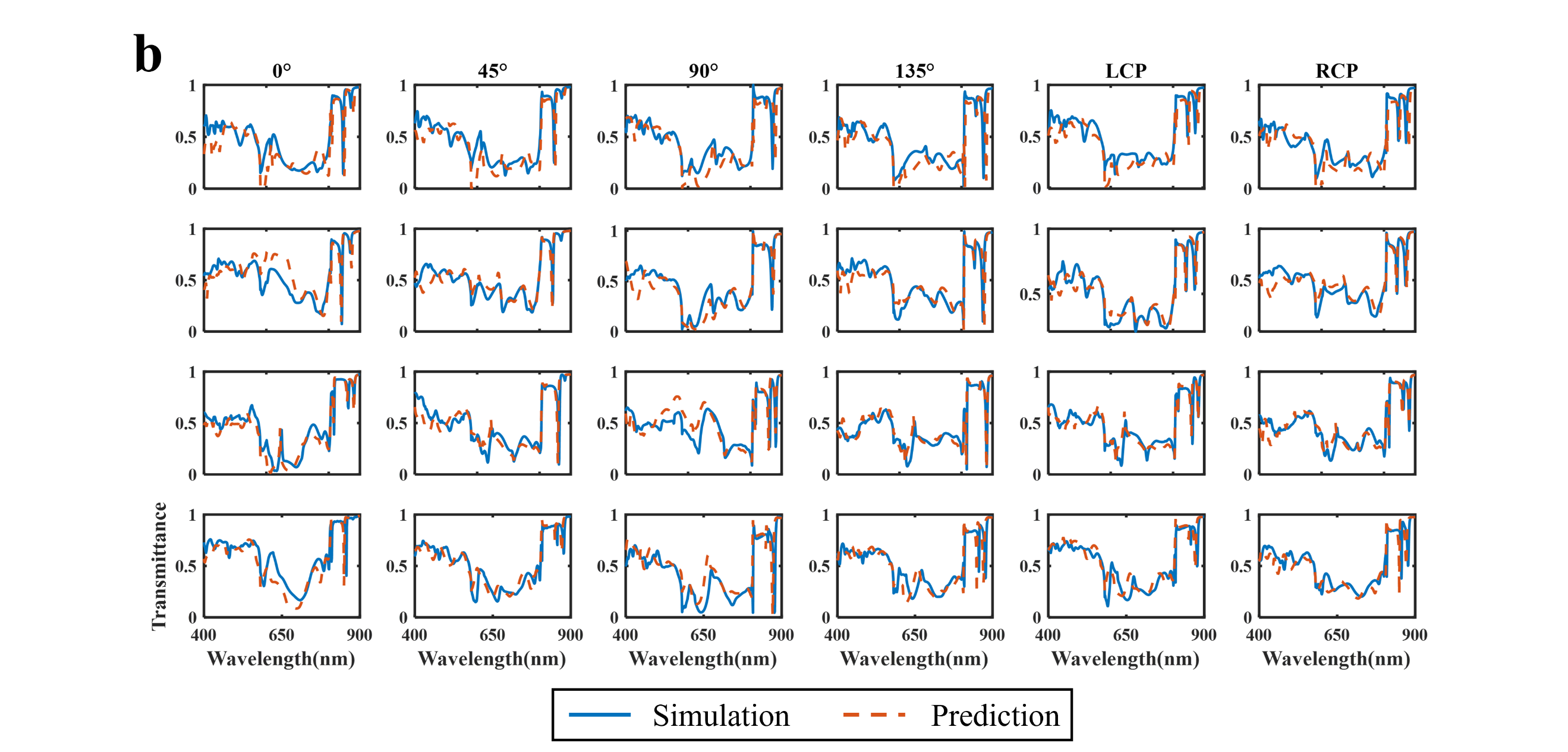

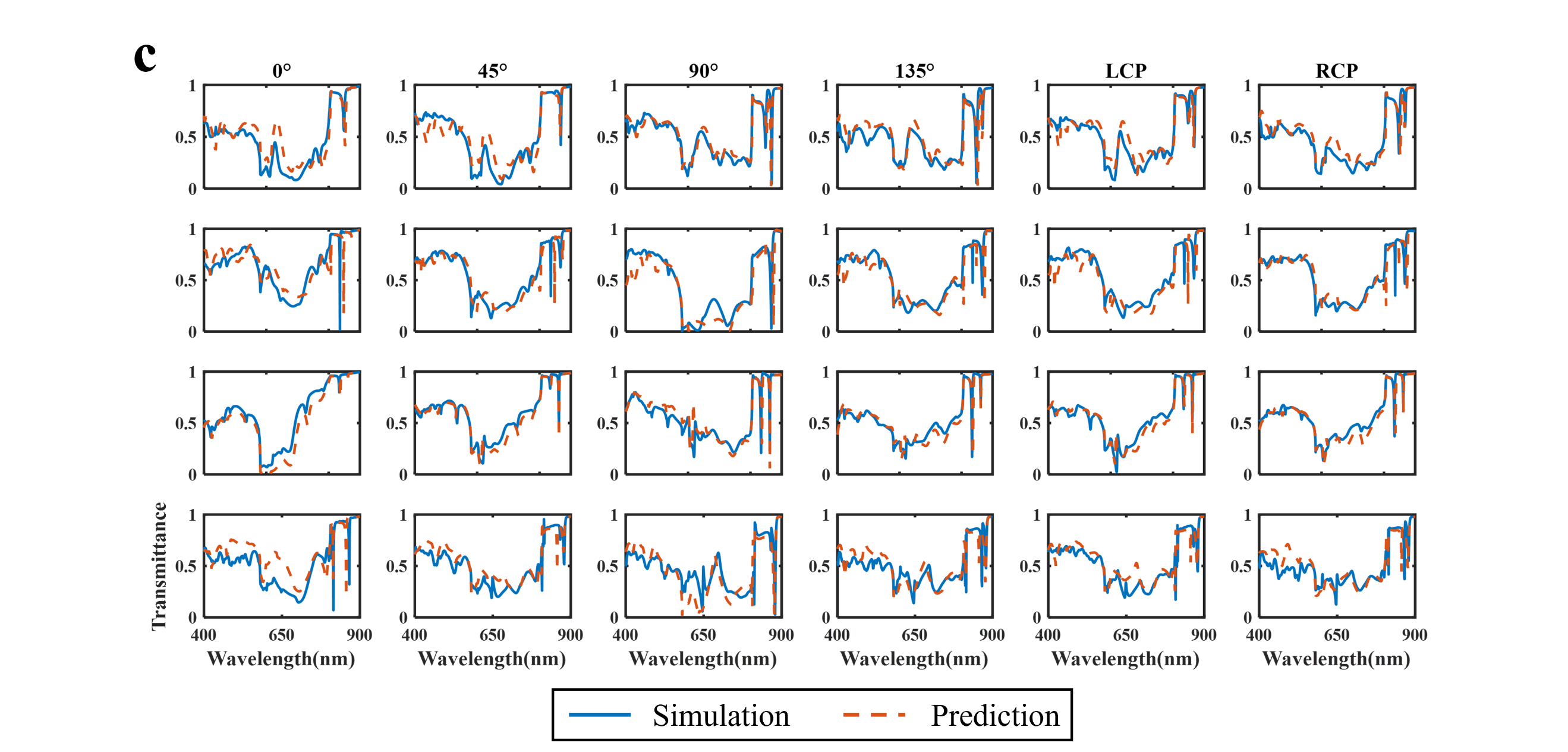


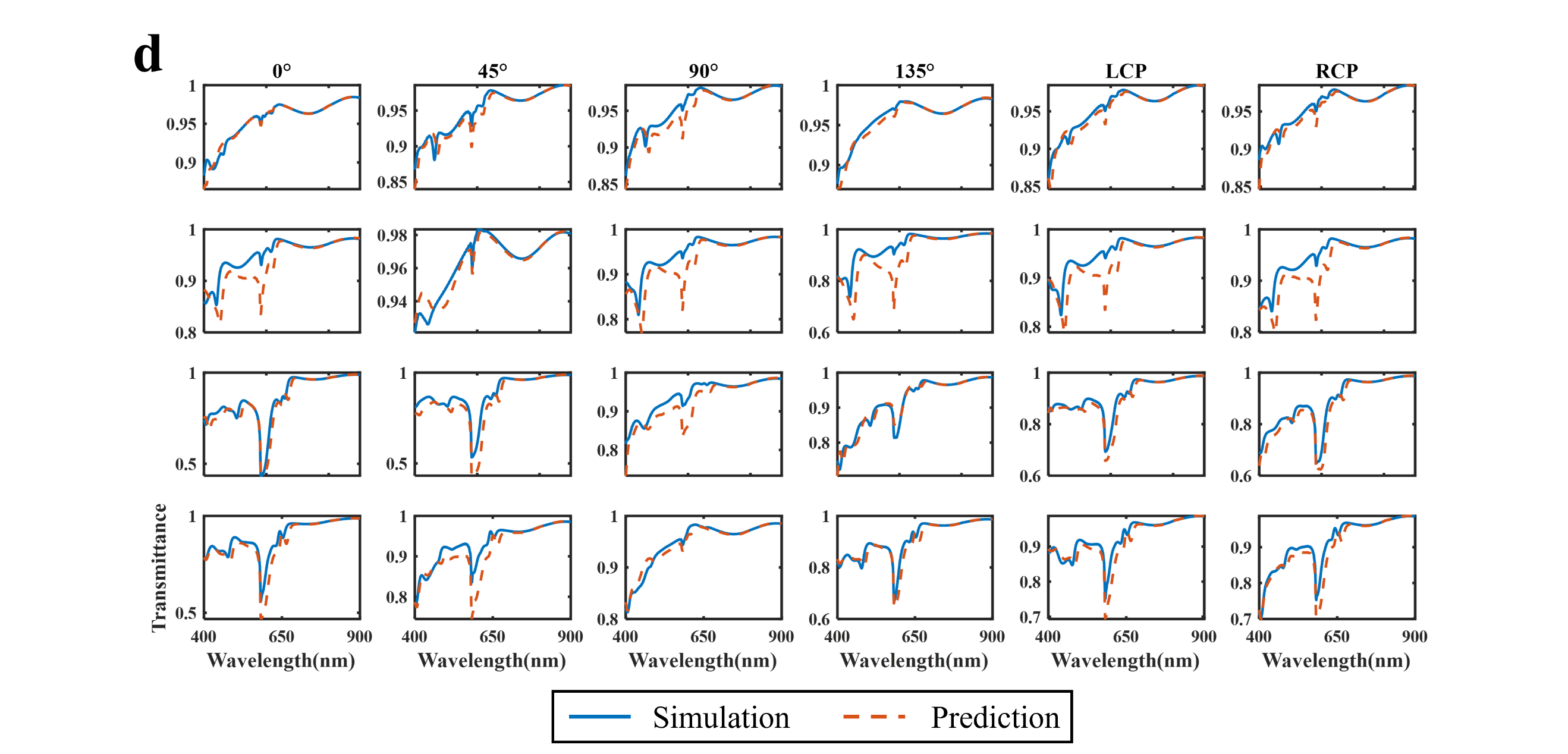


Figure S14. Comparison between the transmittance spectra obtained from simulated and predicted geometric parameters.

**Supplementary Note 9: Arrays of the FHPEM.**

**
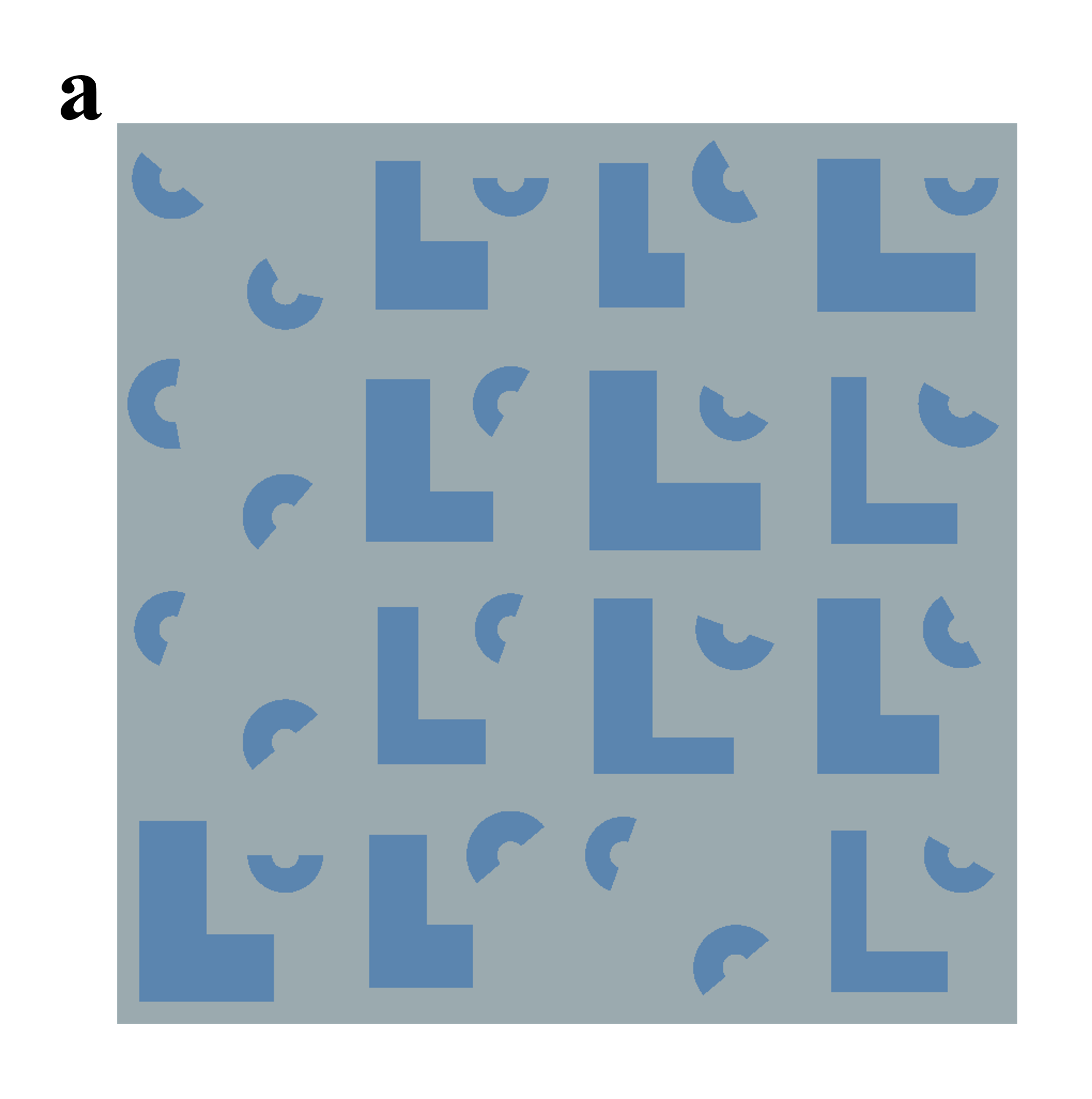

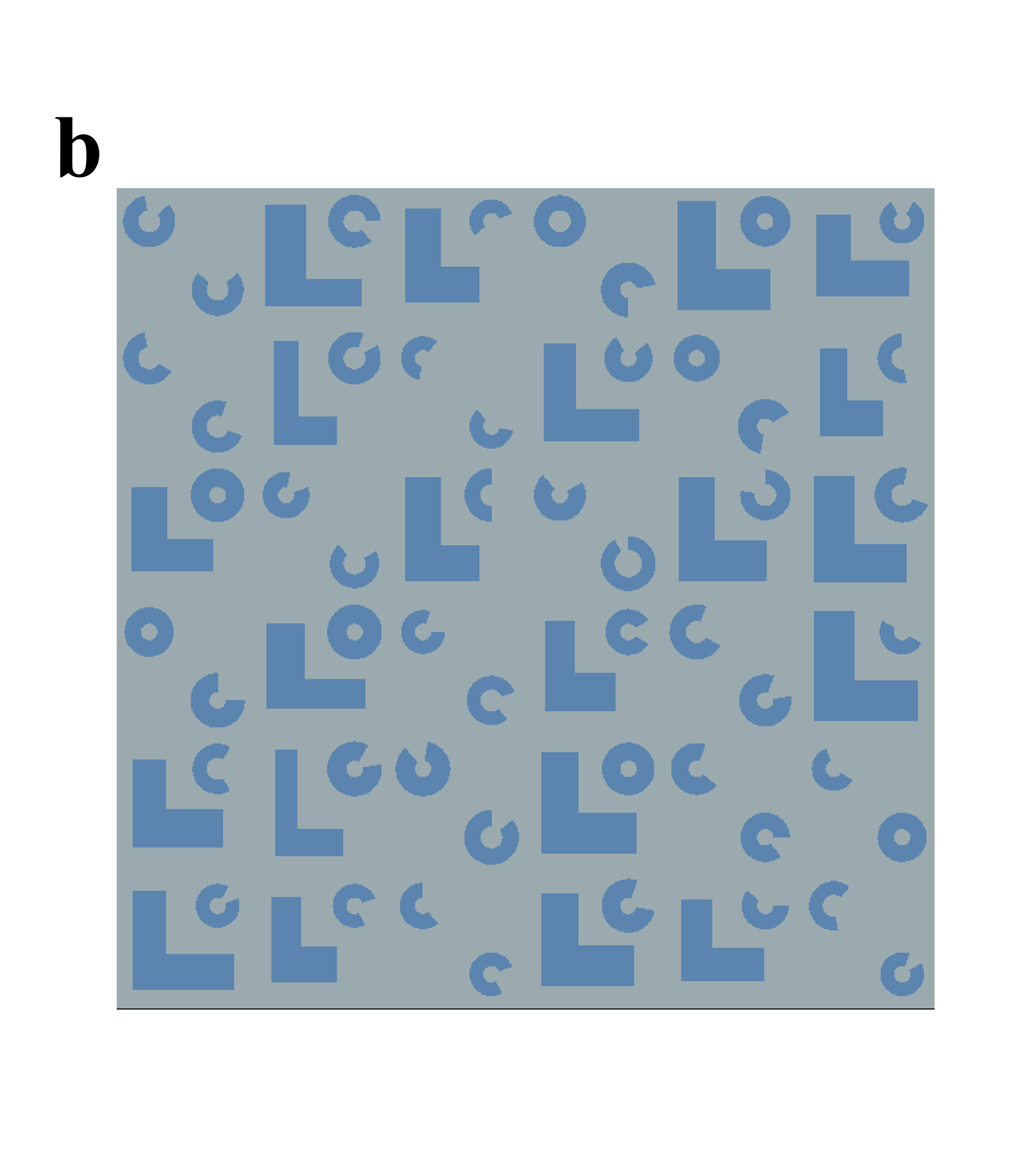

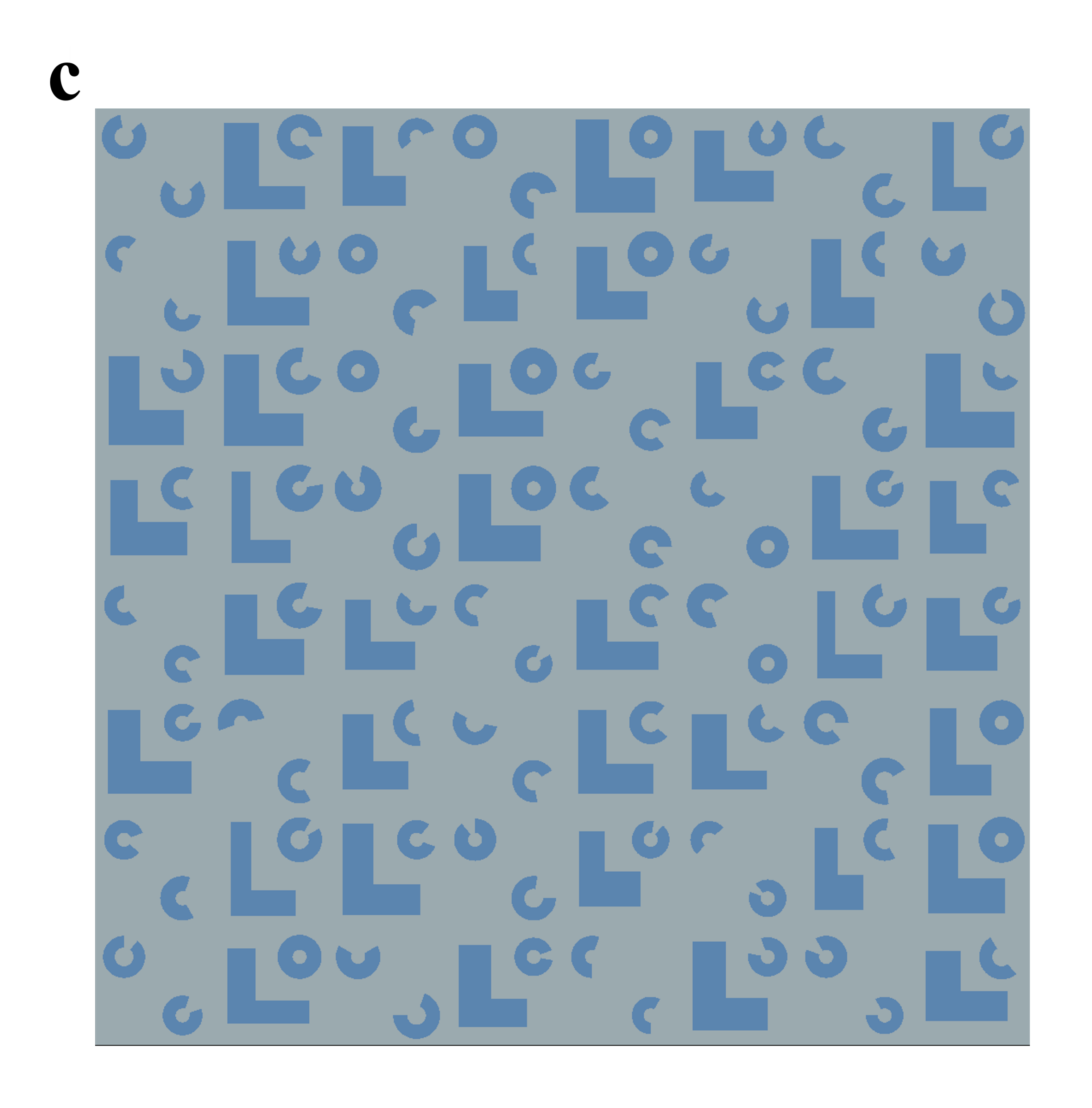

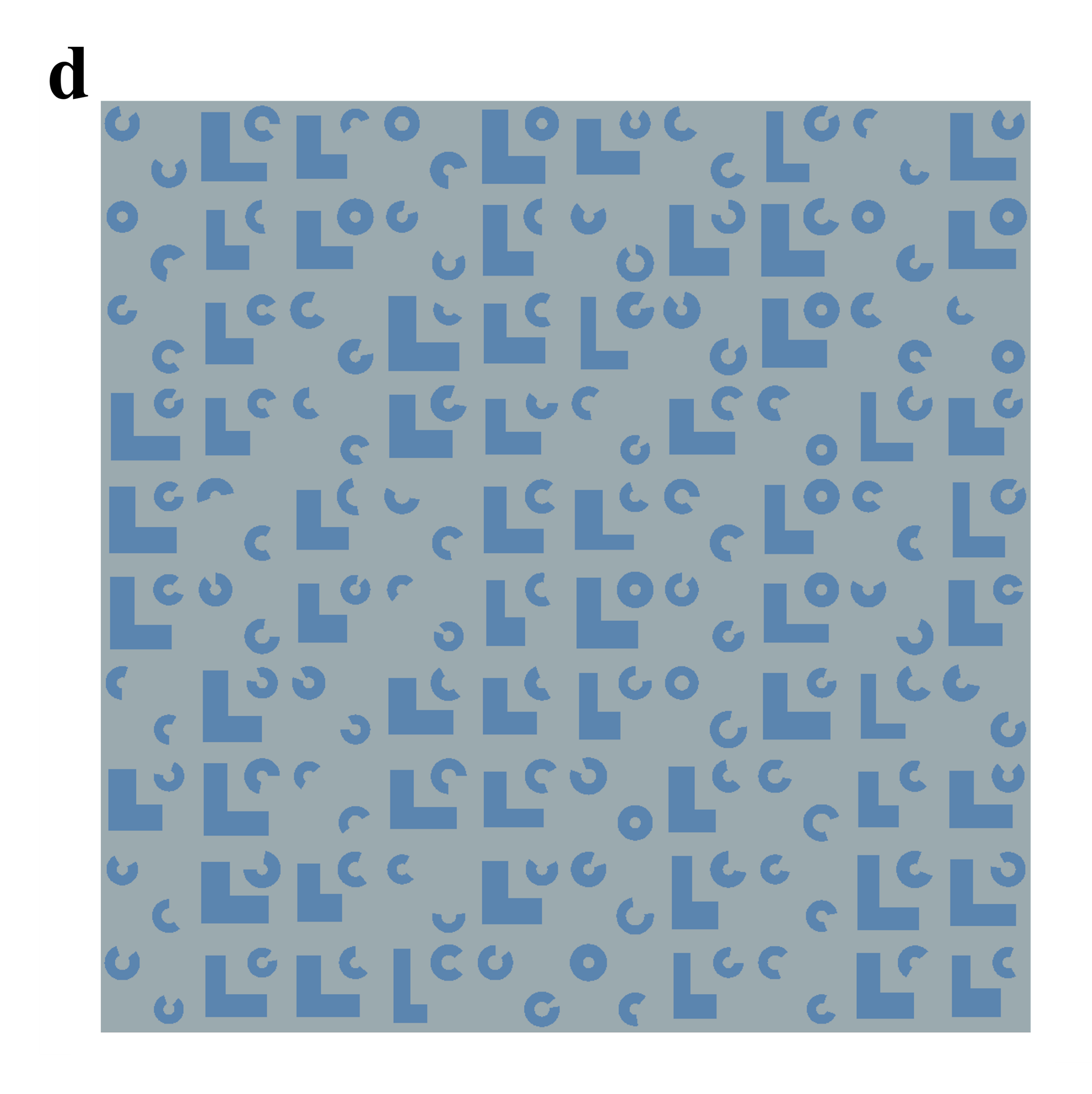
**

Figure S15. Arrays of FHPEM with *n* = 16, 36, 64, and 100.

**Supplementary Note 10: Dataset for the reconstruction network.**

**
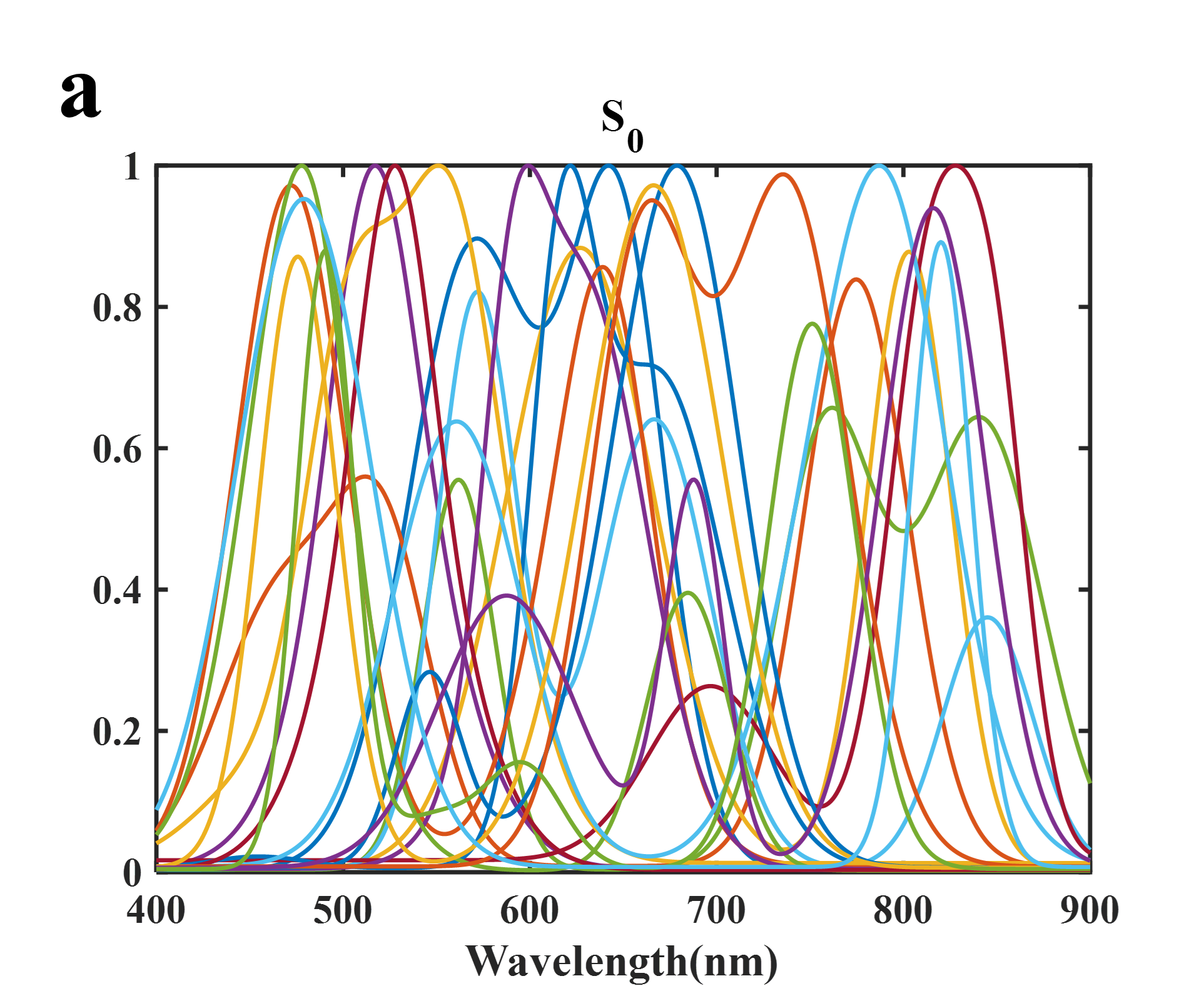

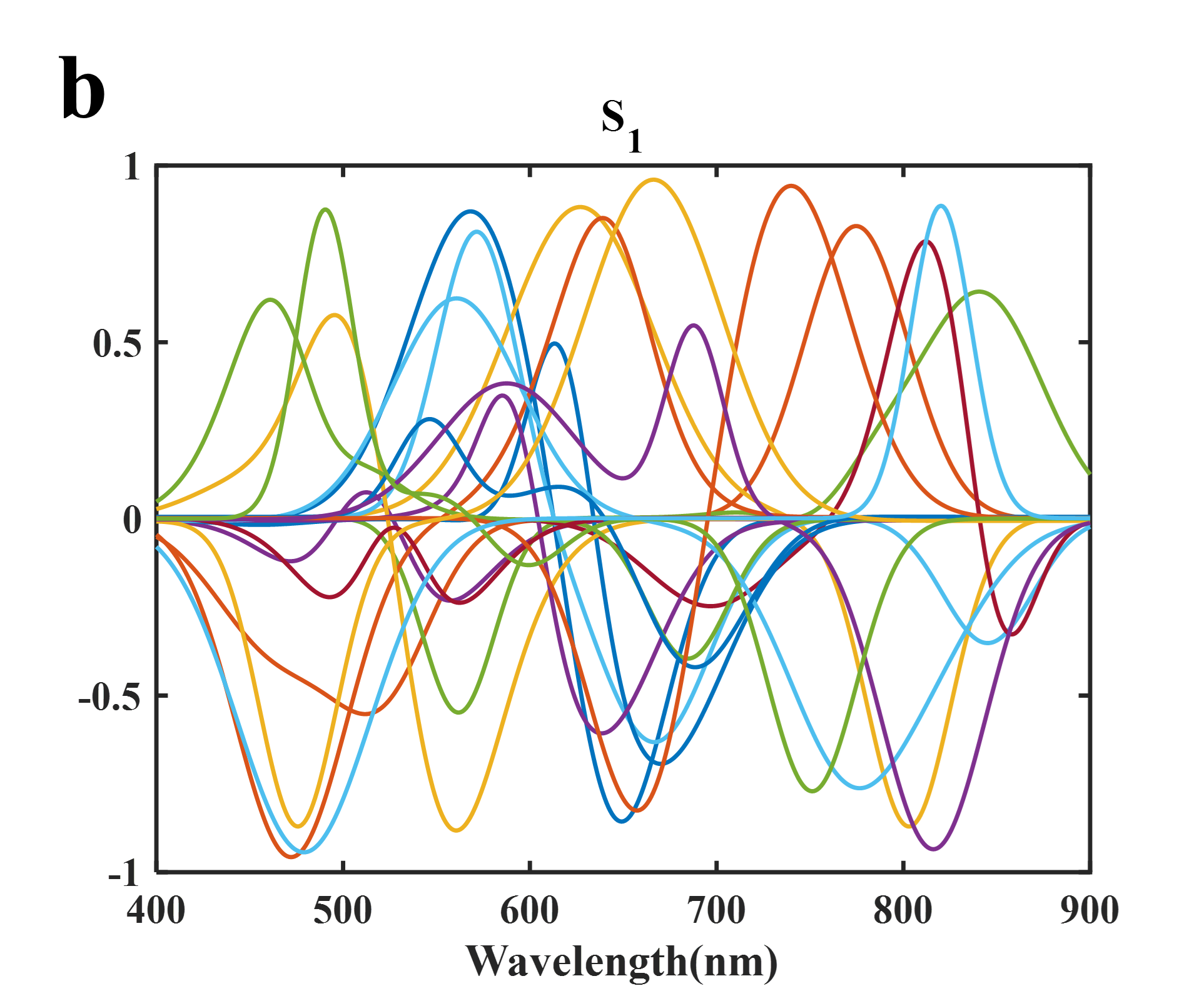

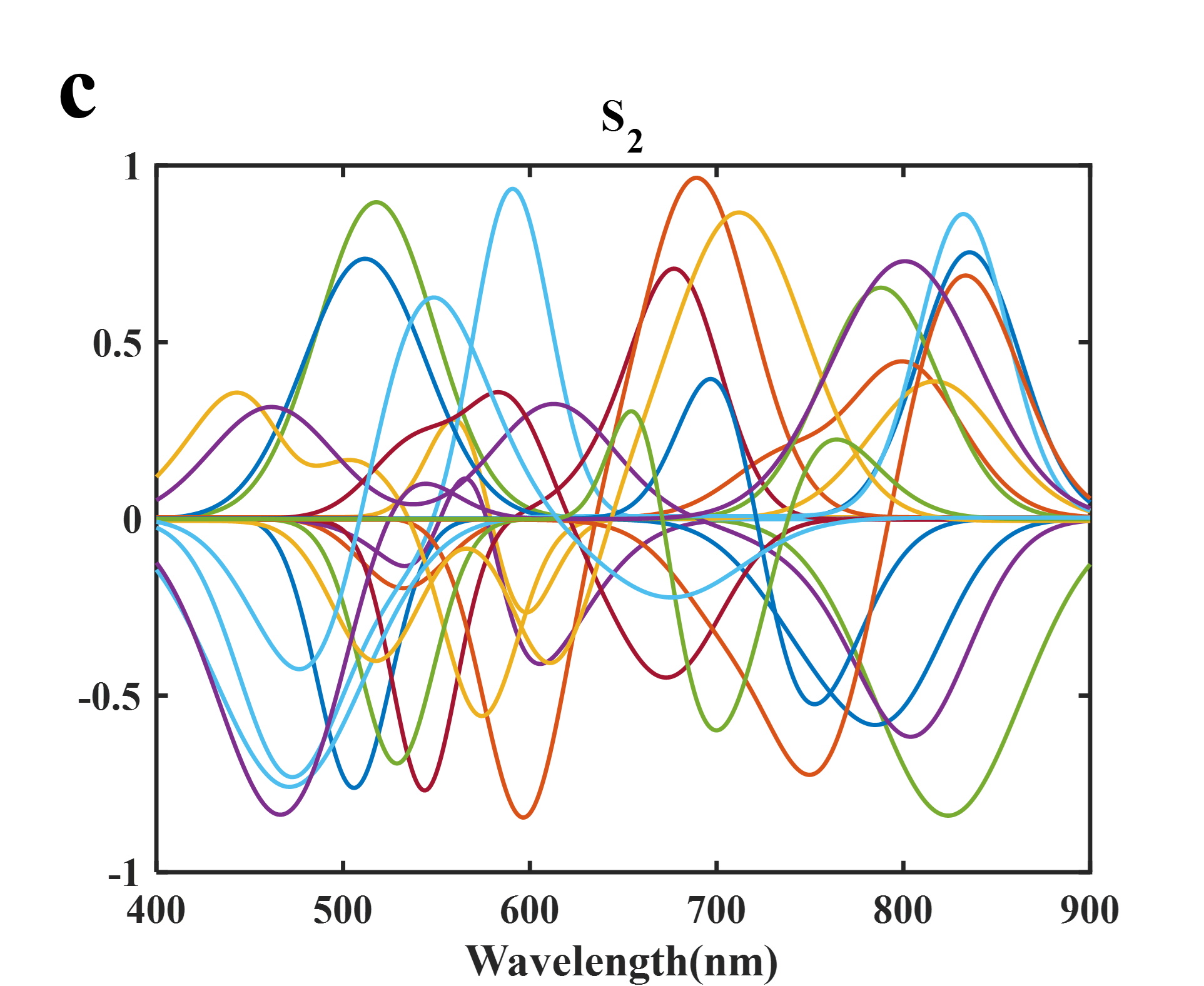

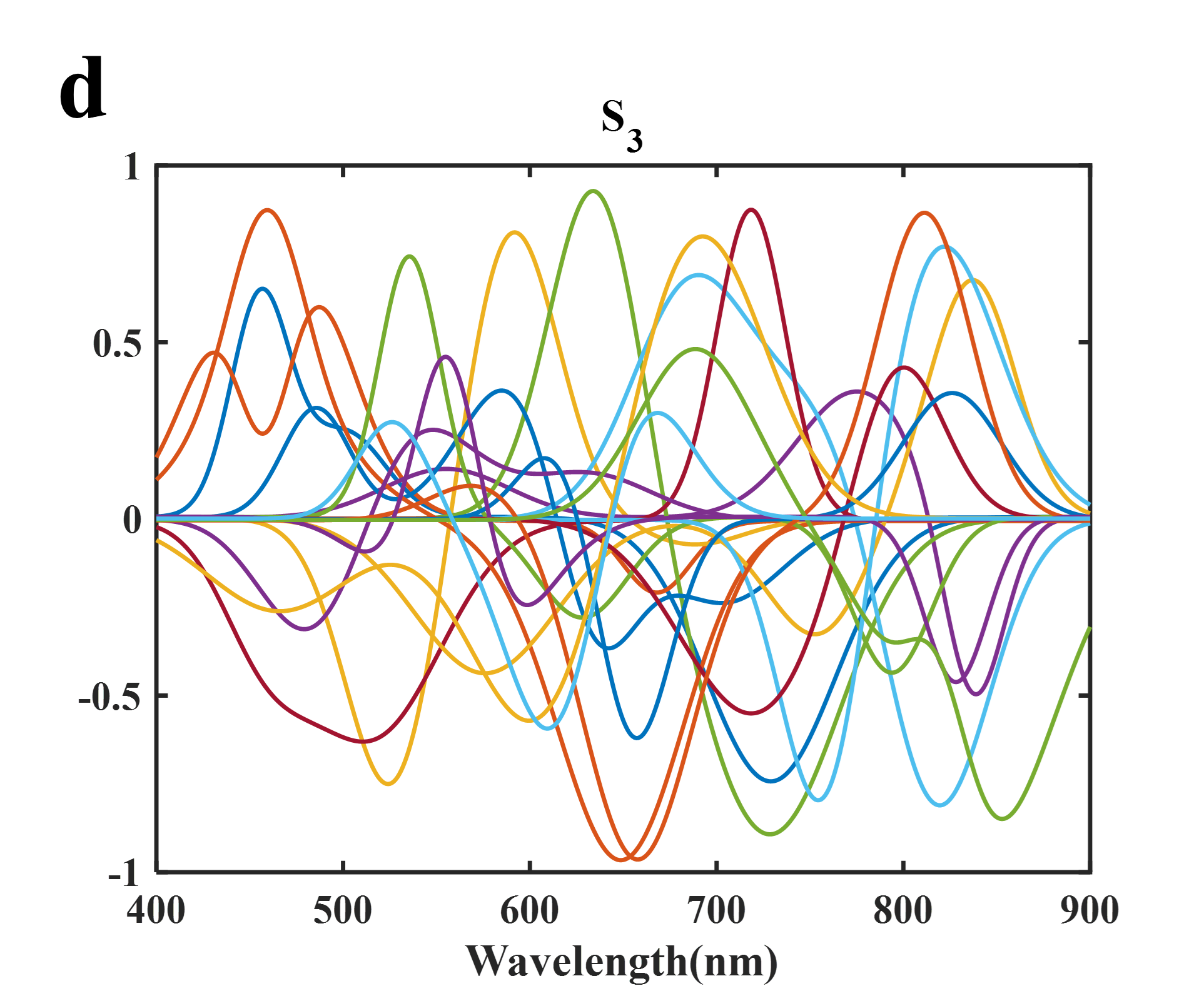
**

Figure S16. Spectro-polarimetric dataset. Each incident spectral polarization information consists of six different polarization states (0°, 45°, 90°, 135° linear polarization, LCP and RCP) forming a full-Stokes vector.

**Supplementary Note 11: Fabrication Details of the FHPEM.**


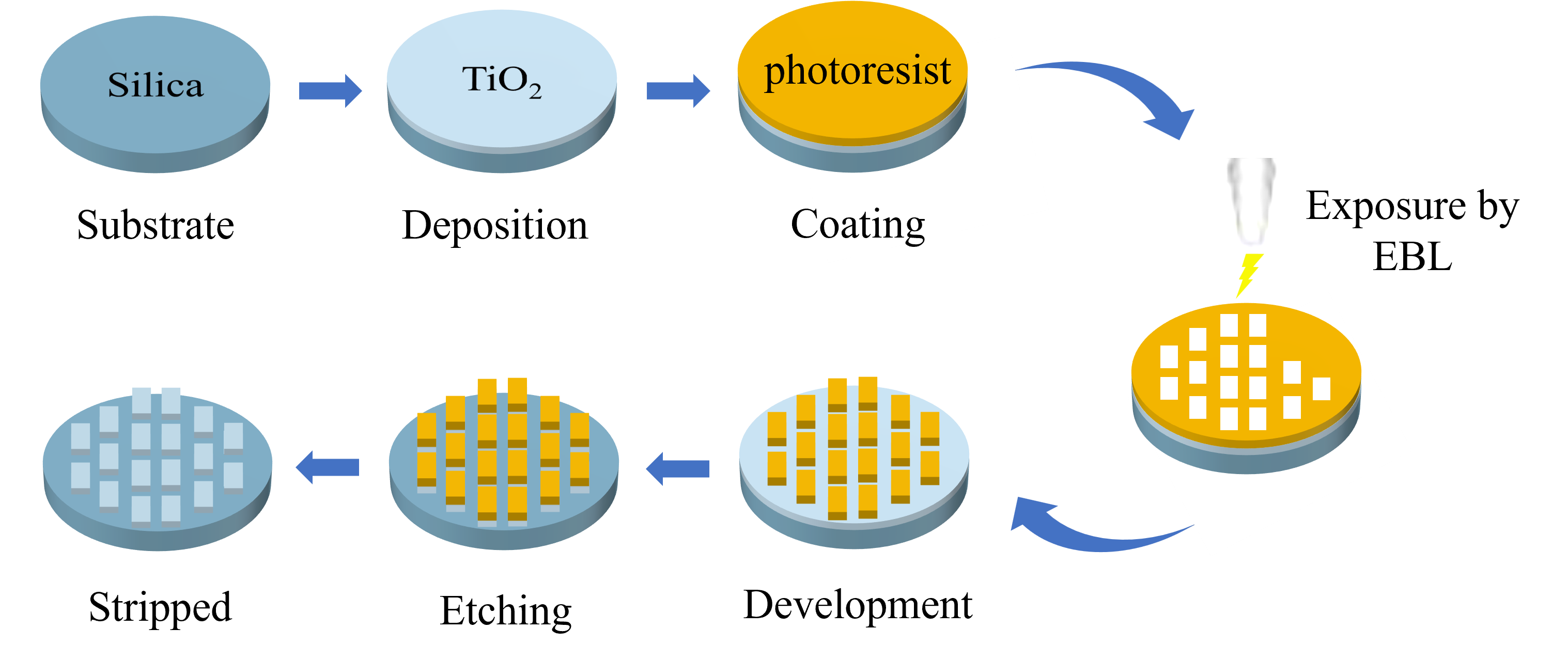


Figure S17. Flowchart of the fabrication process for the sample.

**Supplementary Note 12: Experimental Setup.**

To measure the spectral response of the fabricated metasurface samples under different polarization states, the experimental setup shown in Figure S18 was constructed. Monochromatic light of various wavelengths was first emitted from a monochromator and split by a beam splitter. The reflected beam was directed to a spectrometer to monitor the wavelength of the monochromatic light, while the transmitted beam sequentially passed through a linear polarizer and a quarter-wave plate to generate the desired polarization state. Finally, the light was incident on the metasurface sample and collected by a detector, enabling measurement of the sample’s spectral response under different polarization conditions.


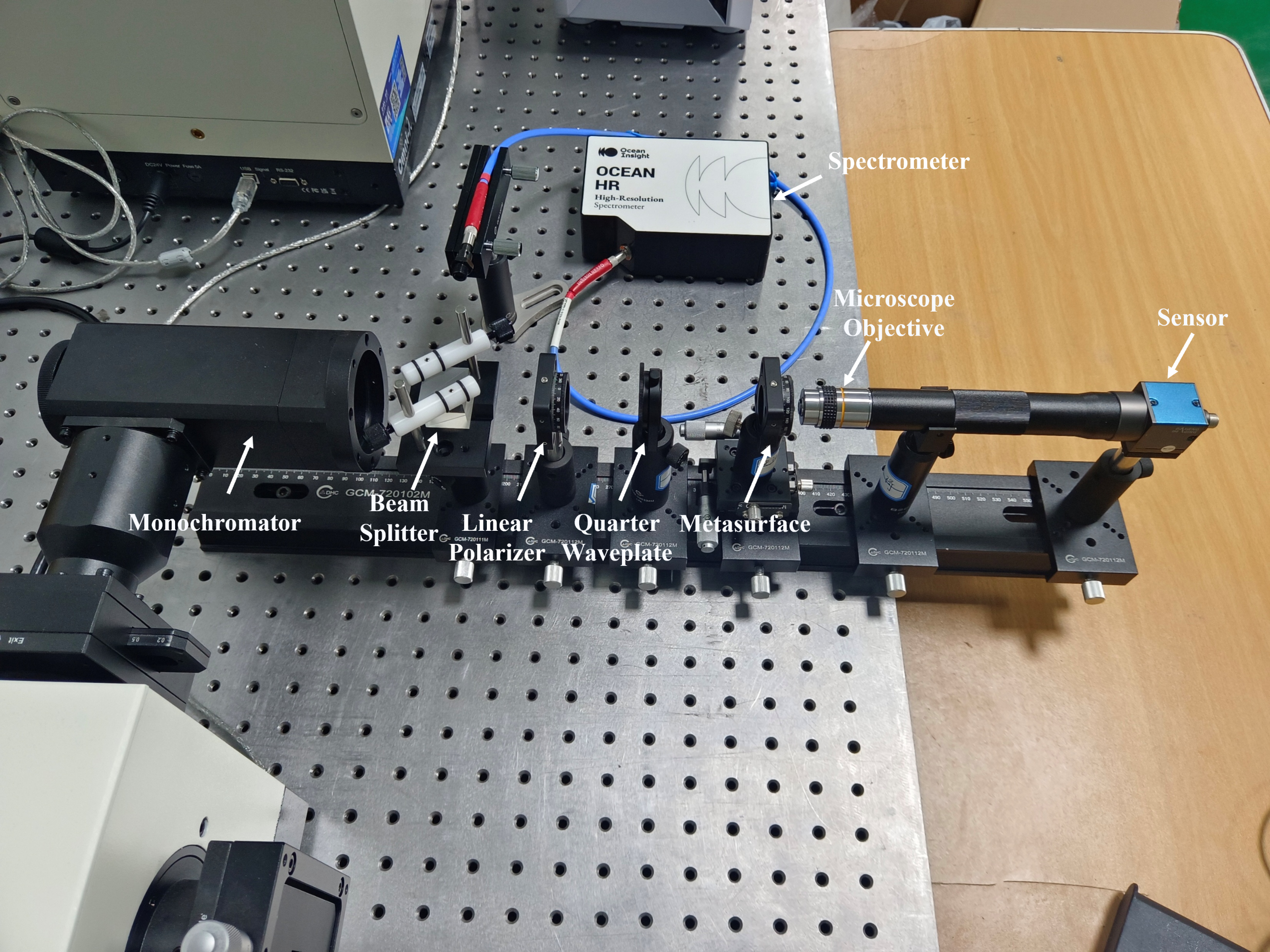


Figure S18. Experimental setup.

**Supplementary Note 13: The SEM images of the metasurface sample.**


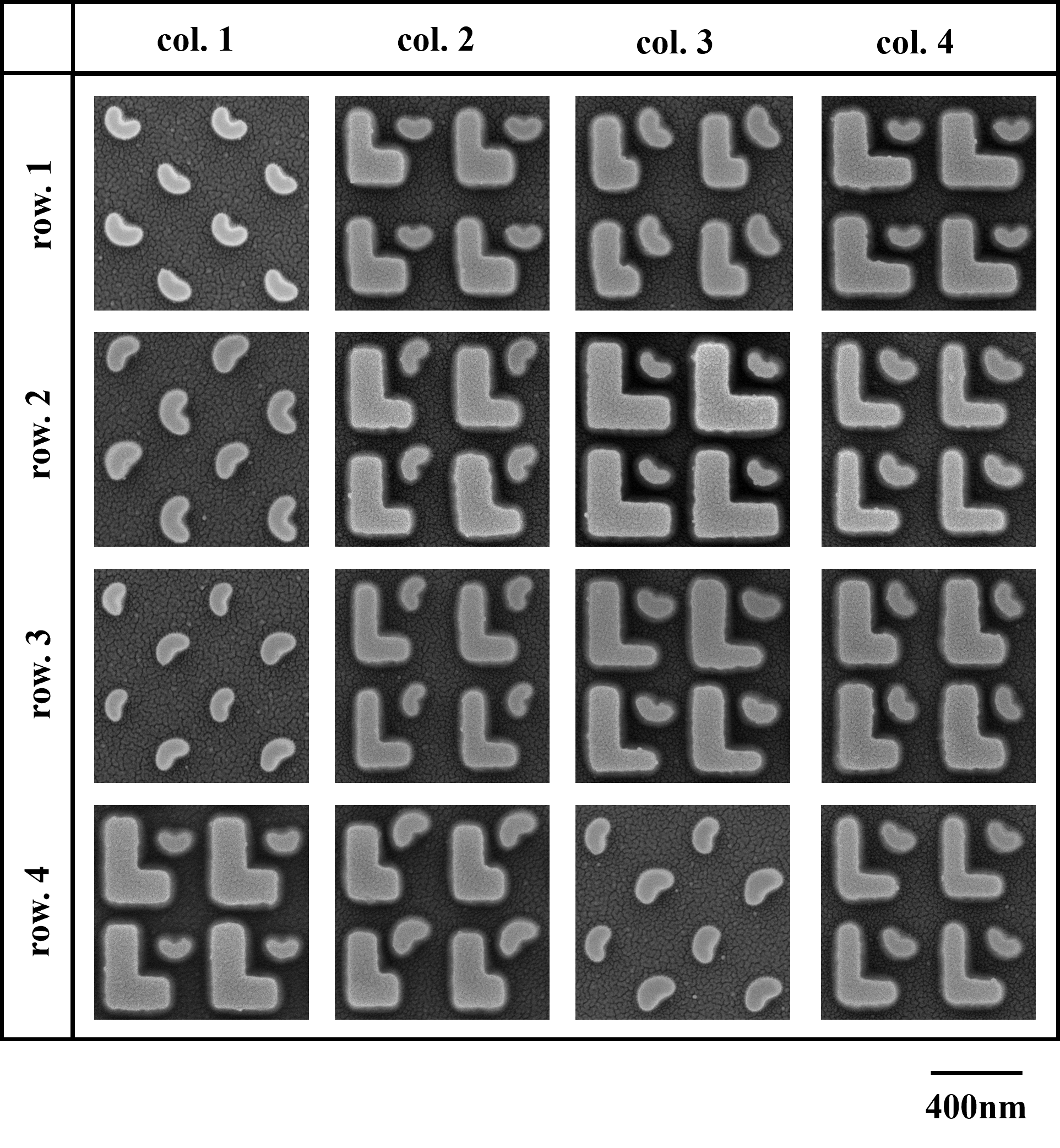


Figure S19. The SEM images of 16 meta-atoms.

**Supplementary Note 14: Experimental setup for spectral resolution.**

To verify the spectral performance of the metasurface sample, the experimental setup shown in Figure S20 was constructed. A monochromator and a 532 nm monochromatic light source were first used, with the monochromator providing light centered around 532 nm. The emitted light was then focused by a beam splitter and passed through a linear polarizer to generate 0° linearly polarized light. Subsequently, the polarized beam was incident on the metasurface sample and detected by a photodetector. Finally, a spectral reconstruction algorithm was applied to the detector images to evaluate the spectral resolution of the metasurface sample.


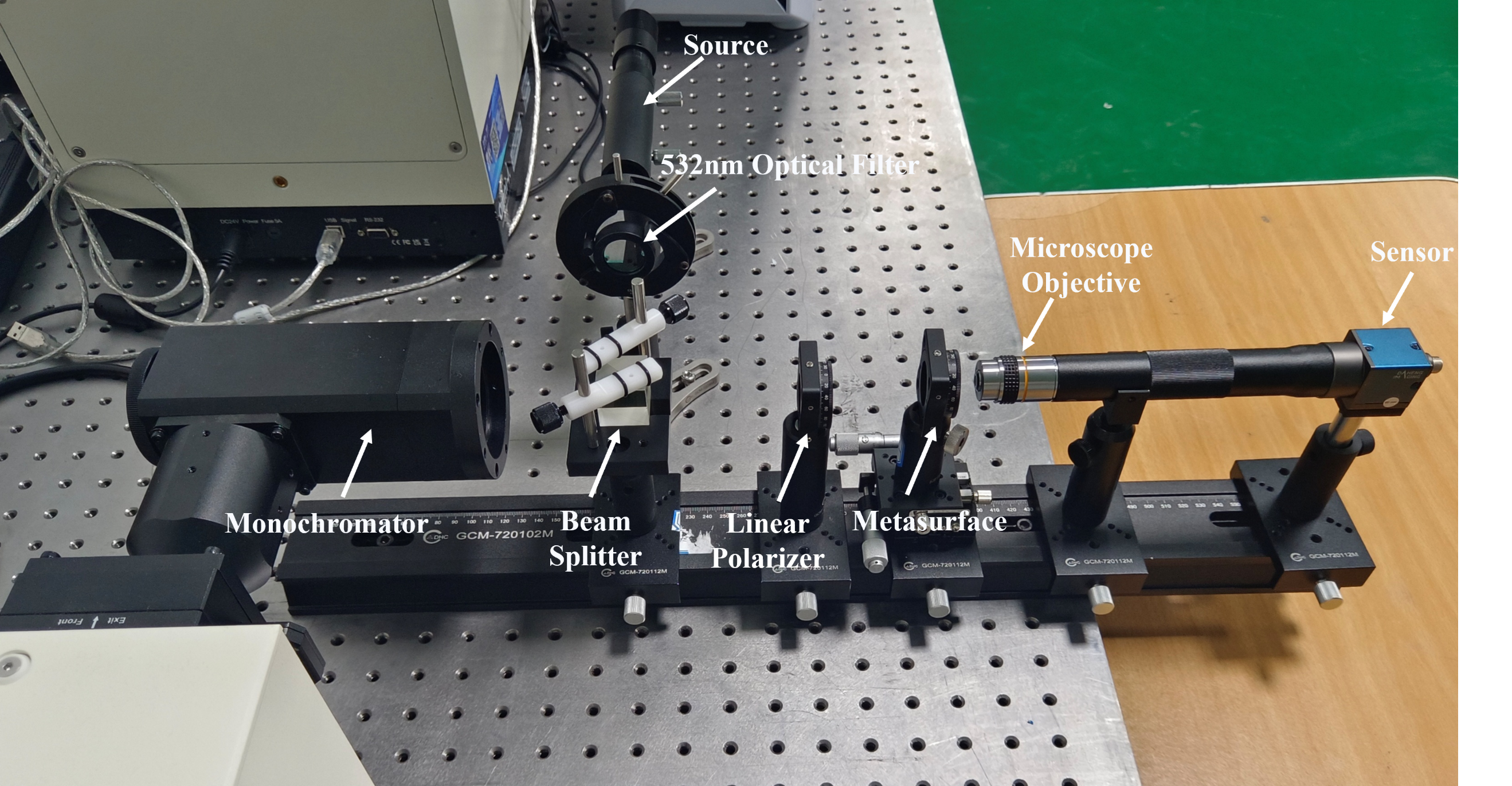


Figure S20. Spectral resolution experimental setup.

**Supplementary Note 15: Experimental setup for the information reconstruction.**

To verify the spectro-polarimetric modulation capability of the metasurface sample, the experimental setup shown in Figure S21 was established. In this setup, light from Source 1 and Source 2, each with different polarization directions, was combined using a beam splitter and then directed onto the metasurface. The transmitted light was subsequently collected by a detector. The polarization directions of Source 1 and Source 2 were adjusted through different combinations of a linear polarizer and a quarter-wave plate, enabling the generation of various polarization combinations.


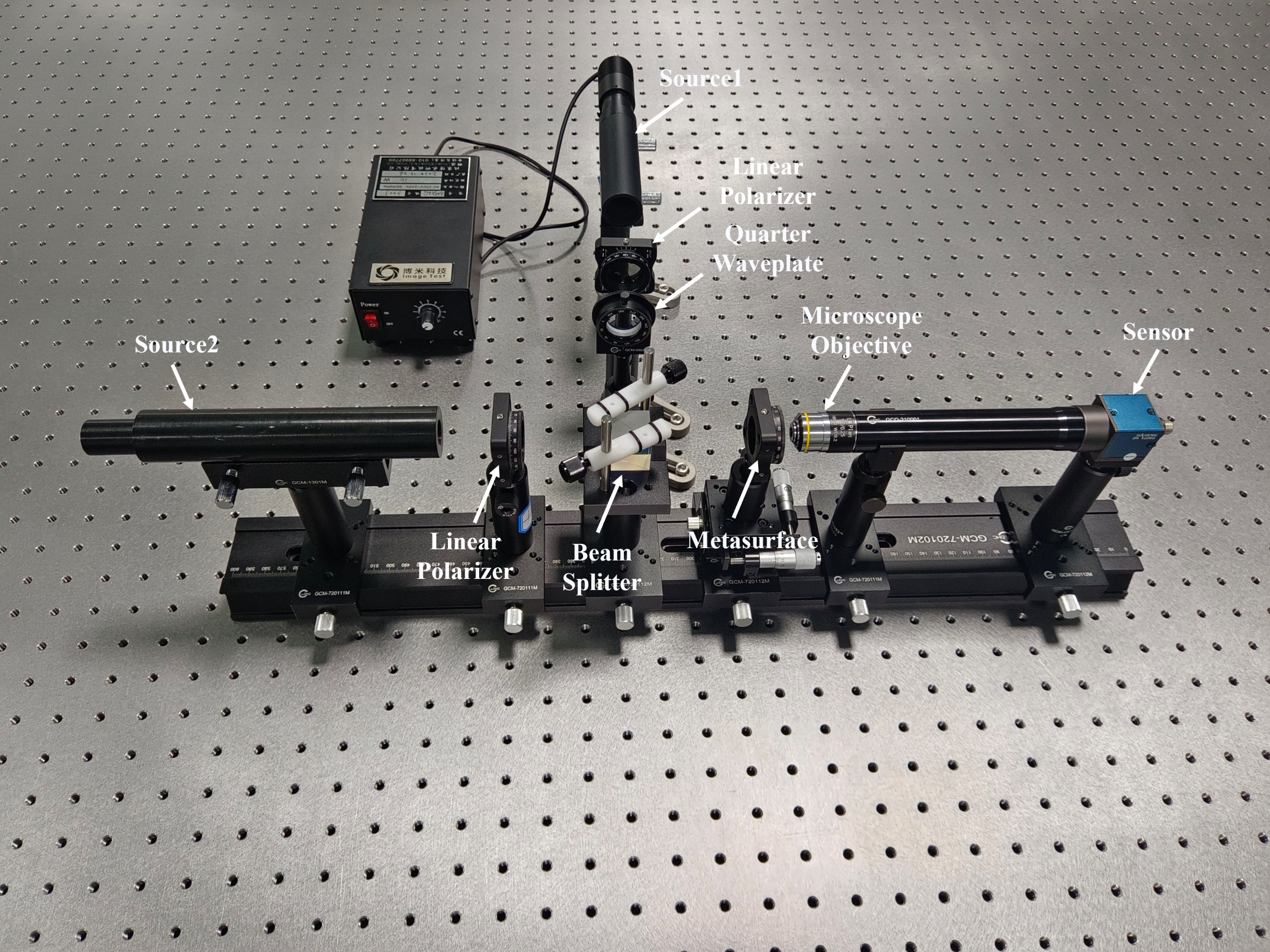


Figure S21. Full-stokes spectro-polarimetric information reconstruction experimental setup.

**Supplementary Note 16: Validation of spectral resolution and reconstruction accuracy under different polarization states.**

We experimentally validated the spectral resolution and broadband reconstruction accuracy of the designed spectro-polarimetric encoding metasurface arrays under incident linear polarizations of 45°, 90°, and 135°, as well as left- and right-handed circular polarizations (LCP and RCP). The experimental setup is shown in Figure S21, and the corresponding results are presented in Figure S22. The results demonstrate that the metasurface arrays consistently achieve a spectral resolution of 4nm under all aforementioned polarization conditions. Moreover, across a broad spectral range, the reconstructed spectra remain in excellent agreement with the ground-truth spectra. The optimal reconstruction performance reaches an MSE of 0.0017, a PSNR of 27.48 dB, and an SSIM of 0.8611, indicating high reconstruction accuracy and robust stability of the system under different polarization states.


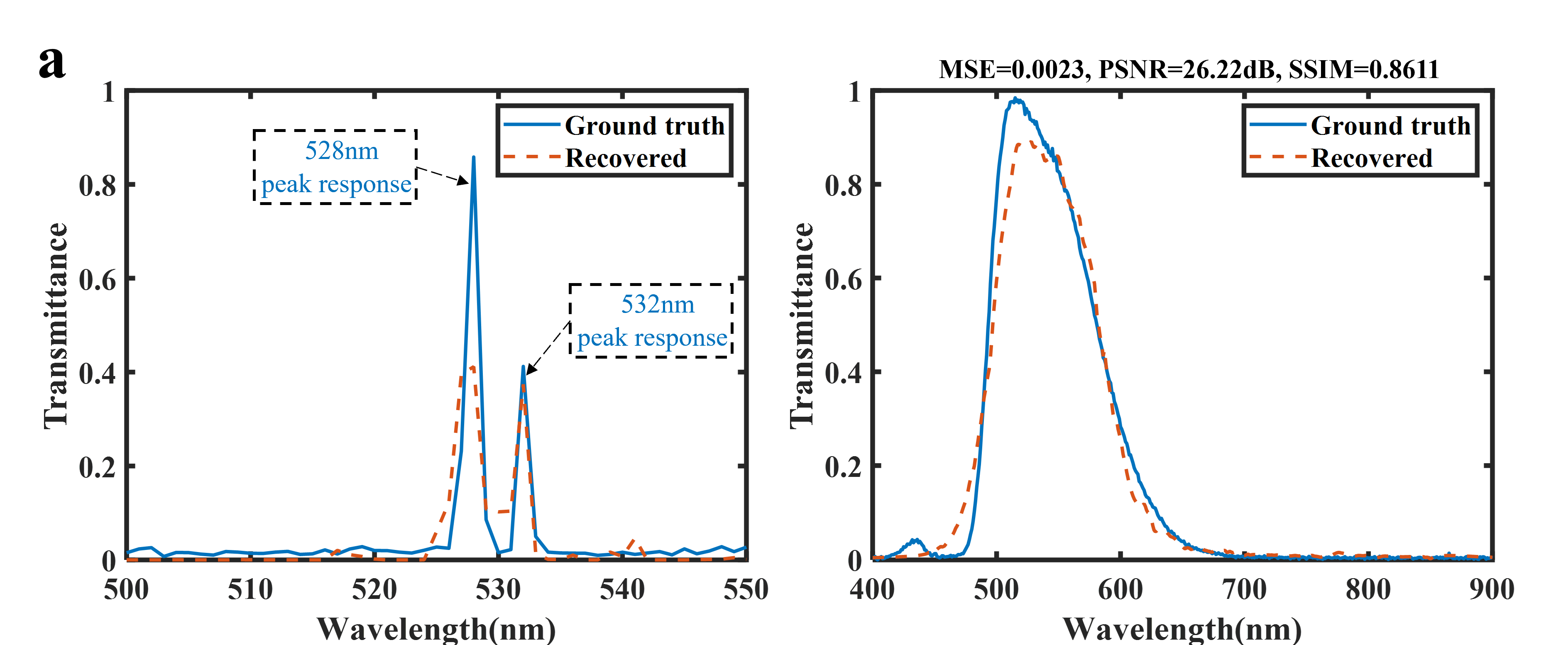

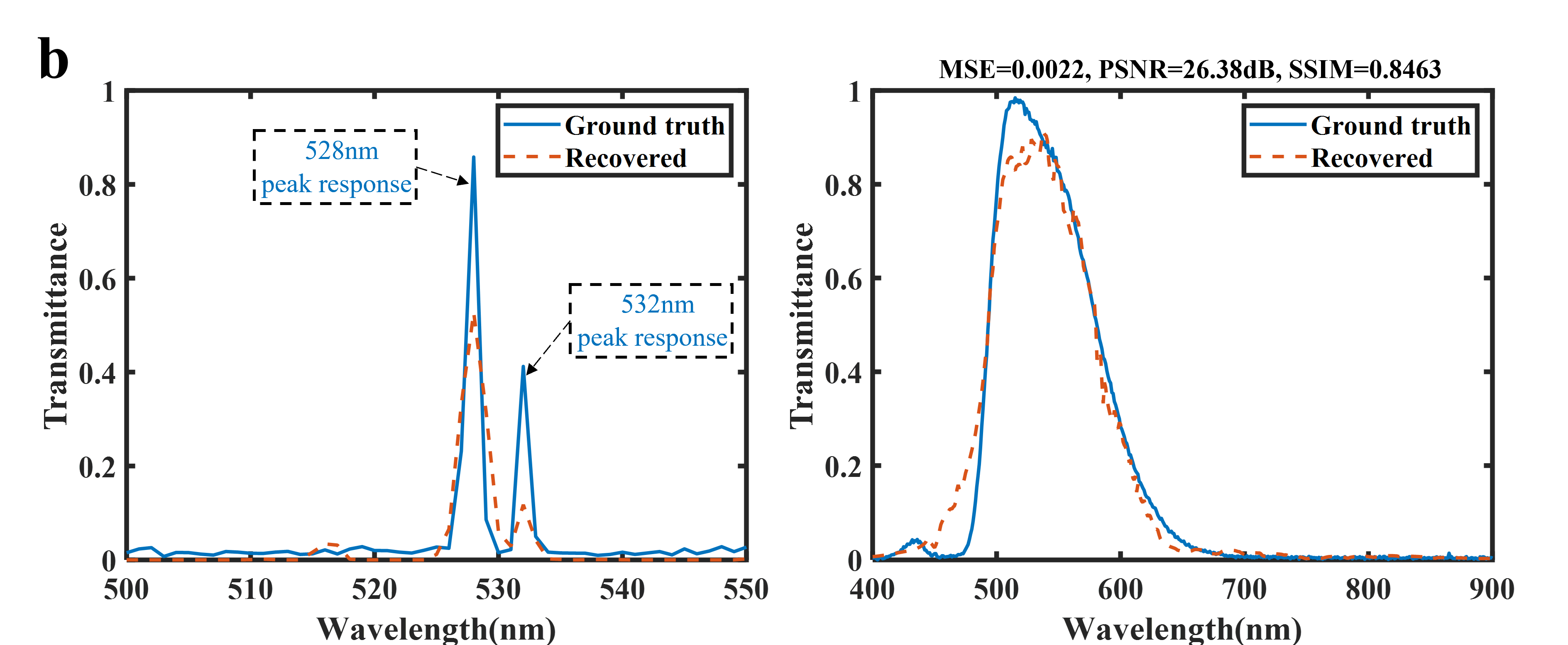


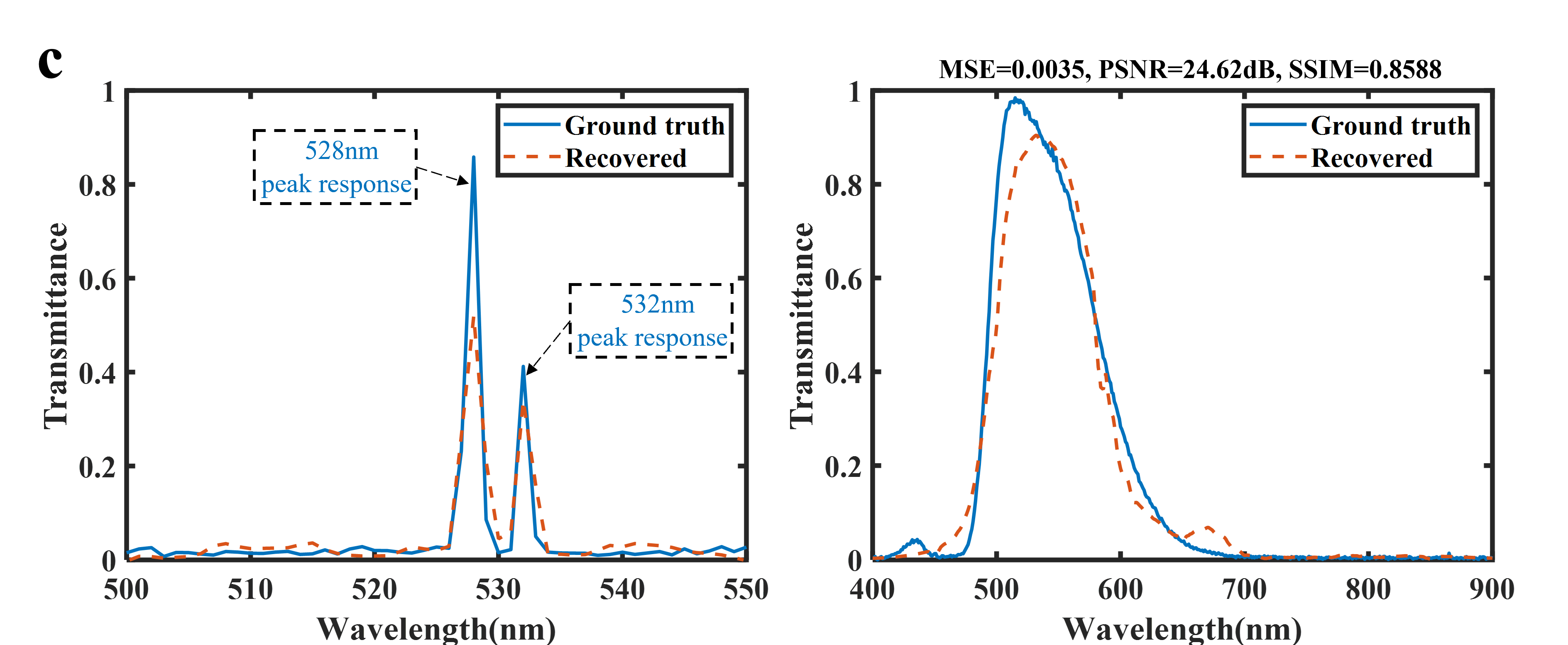


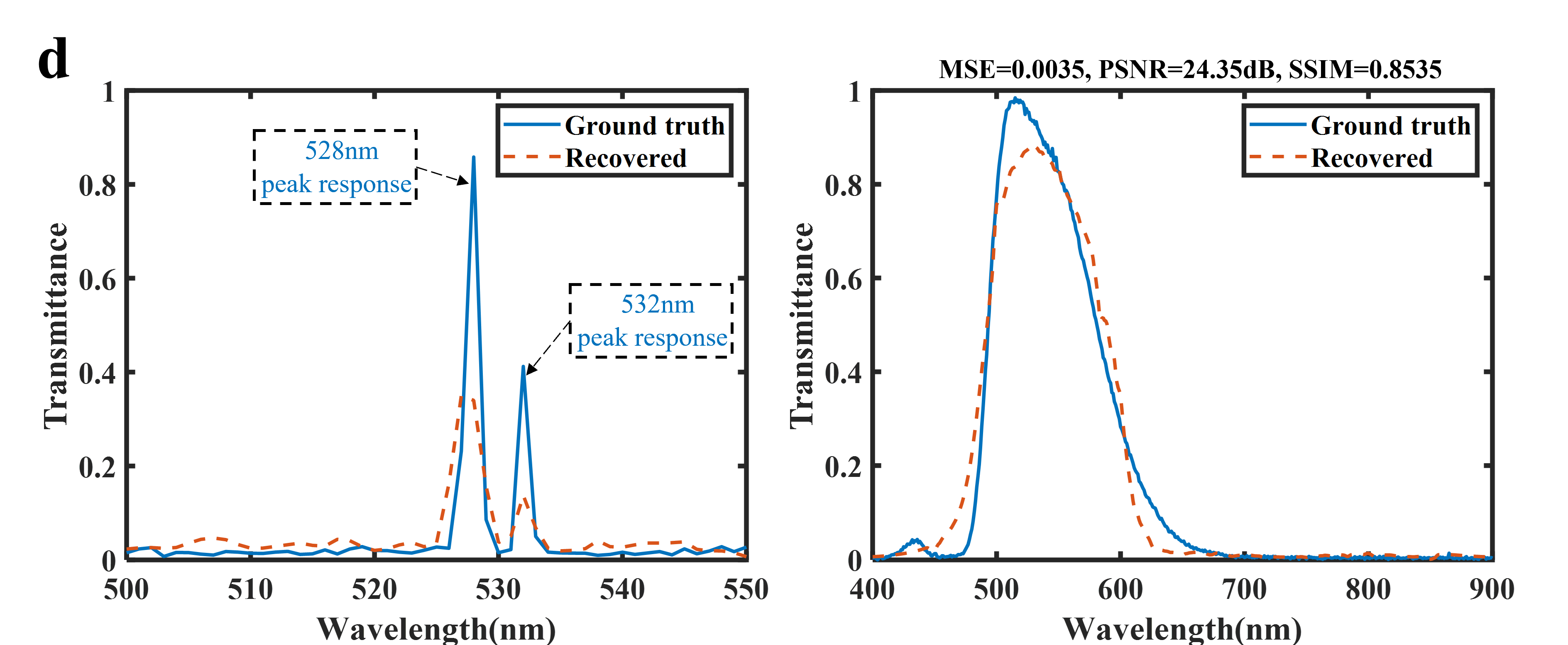


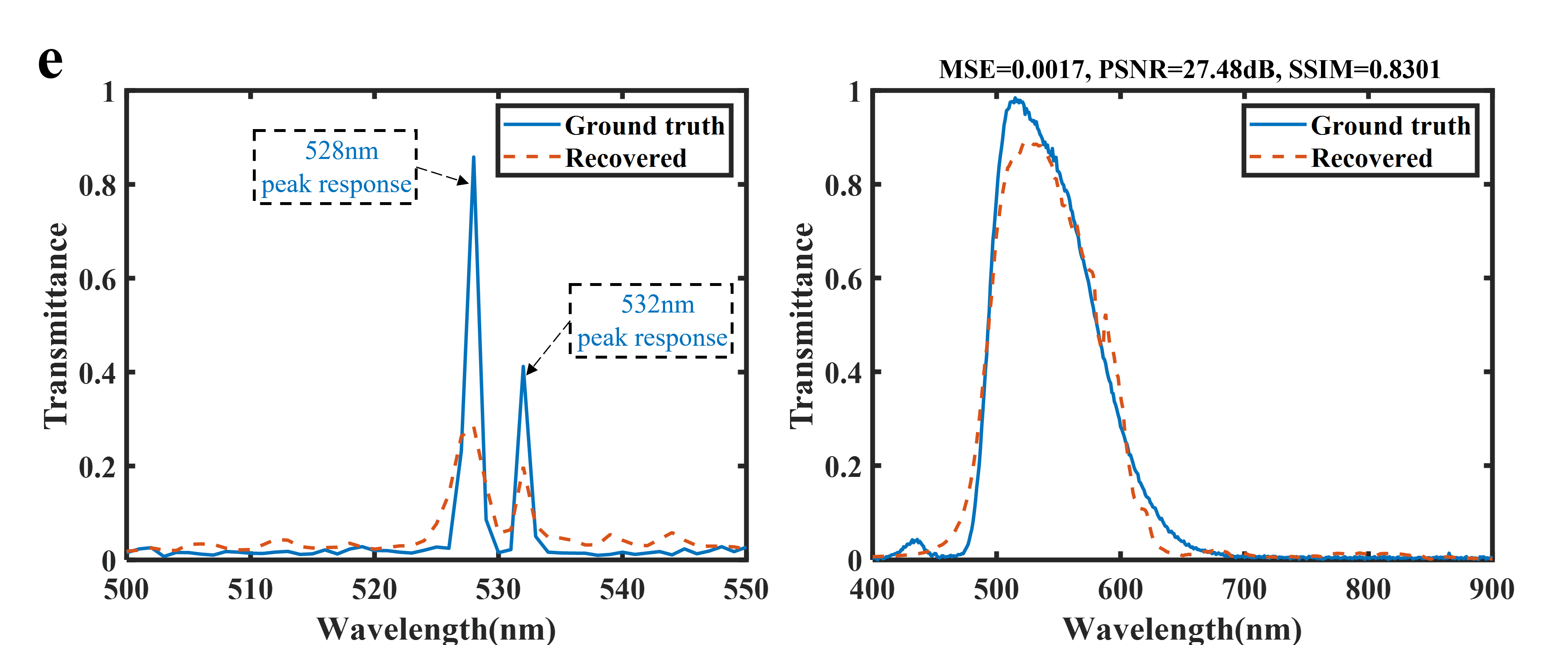


Figure S22. Verification of spectral resolution and broadband spectral reconstruction under different polarization states: (a) 45° linearly polarized incidence; (b) 90° linearly polarized incidence; (c) 135° linearly polarized incidence; (d) LCP polarized incidence; (e) RCP polarized incidence.

**Supplementary Note 17: Experimental setup for spectro-polarimetric imaging** **and dataset construction.**

To further validate the spectro-polarimetric imaging capability of the designed metasurface arrays, a spectro-polarimetric imaging experimental system was established. By colocating the metasurface-based imaging system with a push-broom spectro-polarimetric imaging system [7] under identical imaging conditions, a spectro-polarimetric encoding metasurface dataset was constructed for network training and evaluation. The experimental scene is illustrated in Figure S23. Since the push-broom spectro-polarimetric imaging system supports only linear polarization measurements at 0°, 45°, 90°, and 135°, circular polarization information is not directly accessible. To acquire circular polarization data, a quarter-wave plate was placed in front of the system [8], and the fast-axis orientation was determined through prior calibration. This configuration enables the acquisition of left-handed and right-handed circular polarization (LCP and RCP) images, allowing full-Stokes polarization information to be collected.

Considering the data scale requirements of the reconstruction network, data augmentation was applied to the originally captured images, including random cropping and rotation. Due to slight differences in the field of view and pixel mapping between the two imaging systems, spatial misalignment may occur between the encoded images and their corresponding spectro-polarimetric images. To address this issue, scale-invariant feature transform (SIFT) registration was employed to align the two image types, achieving precise spatial correspondence. This alignment provides a reliable data foundation for subsequent network training and reconstruction. In total, 500 pairs of encoded images and corresponding full-Stokes hyperspectral images were obtained for network training and validation. The image size is 1024×1224, and the spectral dimension is set to 41.

In addition, to eliminate measurement discrepancies introduced by differences in detector quantum efficiency (QE), optical response, and imaging parameters between the two systems, radiometric calibration was independently performed for both the metasurface encoding imaging system and the push-broom spectro-polarimetric system [9]. This calibration process removes system response inconsistencies while preserving the true spectral radiance distribution of the scene, thereby ensuring data comparability between the two systems. As a result, the calibrated data more accurately represent the actual radiative energy distribution of the target scene across spectral and polarization channels, effectively avoiding systematic biases caused by detector response variations.


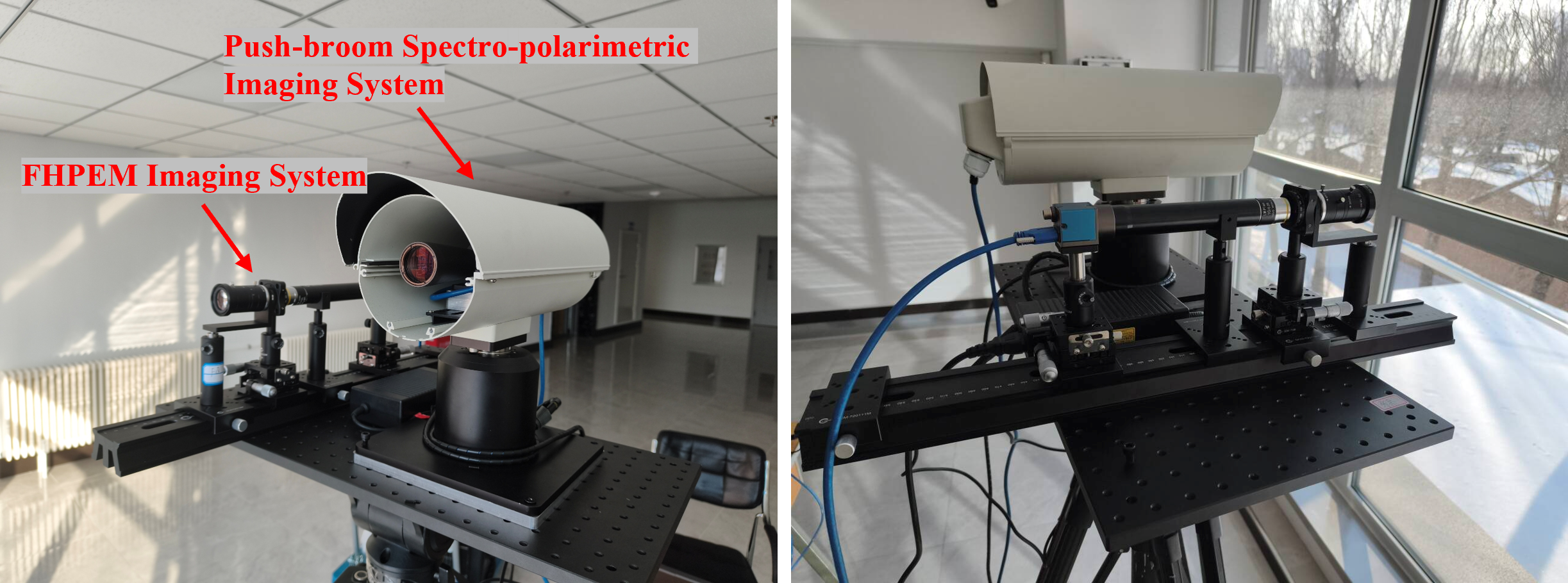


Figure S23. Experimental setup for spectro-polarimetric imaging.

**Supplementary Note 18: Spectro-Polarimetric Image Reconstruction Network.**

During the construction of the reconstruction network, a U-Net architecture [10] was adopted for spectro-polarimetric image reconstruction. This choice is motivated by the characteristic encoder-decoder structure of U-Net, which employs skip connections to effectively fuse multi-scale features during the downsampling and upsampling processes. Such a design allows the network to preserve both global contextual information and fine spatial details. This capability is particularly important for spectro-polarimetric reconstruction tasks, which require not only the recovery of fine spatial structures from encoded images but also the accurate reconstruction of correlations across different spectral and polarization channels.

In the proposed framework, the input to the network is the spectro-polarimetric encoded image, while the output is the reconstructed spectro-polarimetric image spanning a wavelength range from 450 nm to 850 nm with a total of 41 spectral channels, comprising the four Stokes components (S0, S1, S2, and S3). Therefore, the U-Net architecture is well suited for achieving stable and high-accuracy spectro-polarimetric image reconstruction.


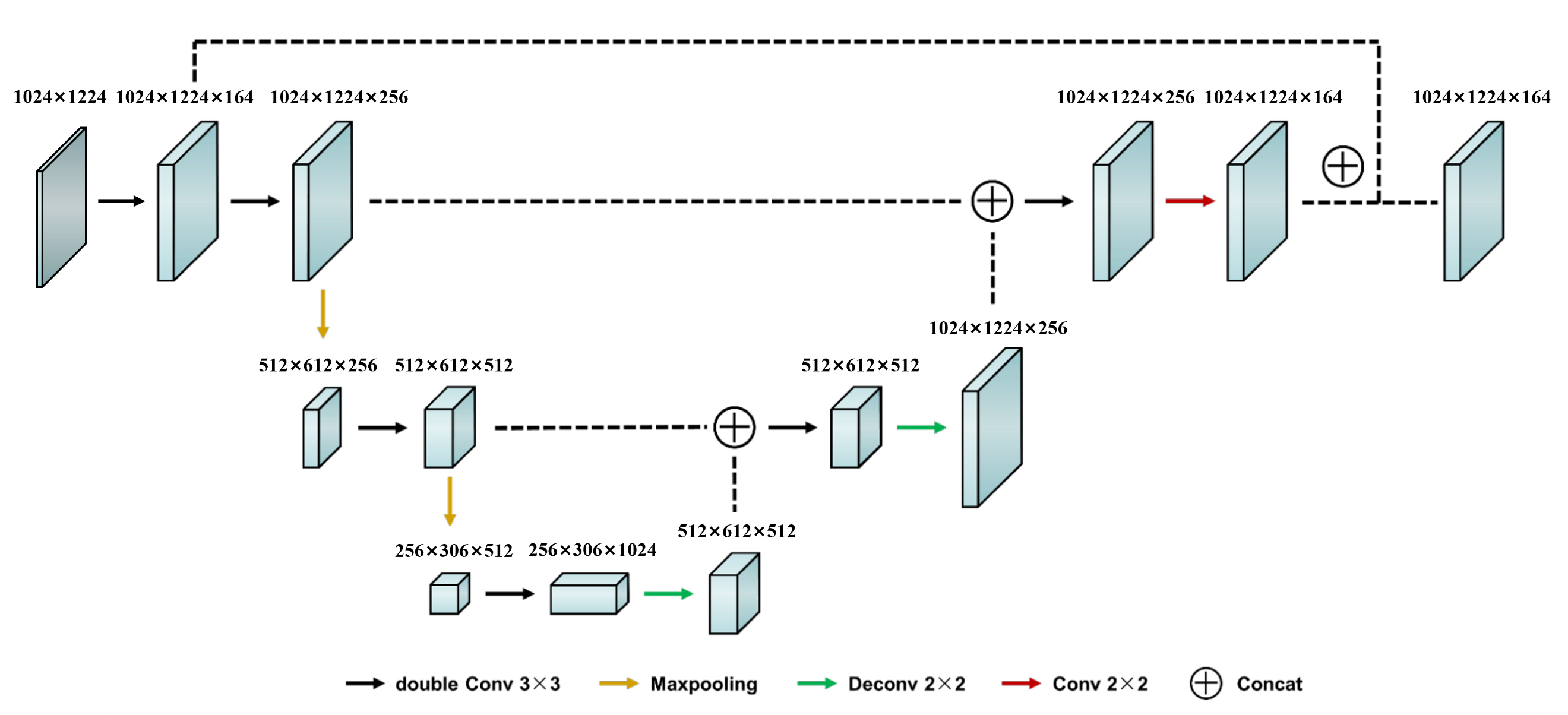


Figure S24. Architecture of the U-Net network.

**Supplementary Note 19:** **Ground-truth spectro-polarimetric images** **for different target scenes.**


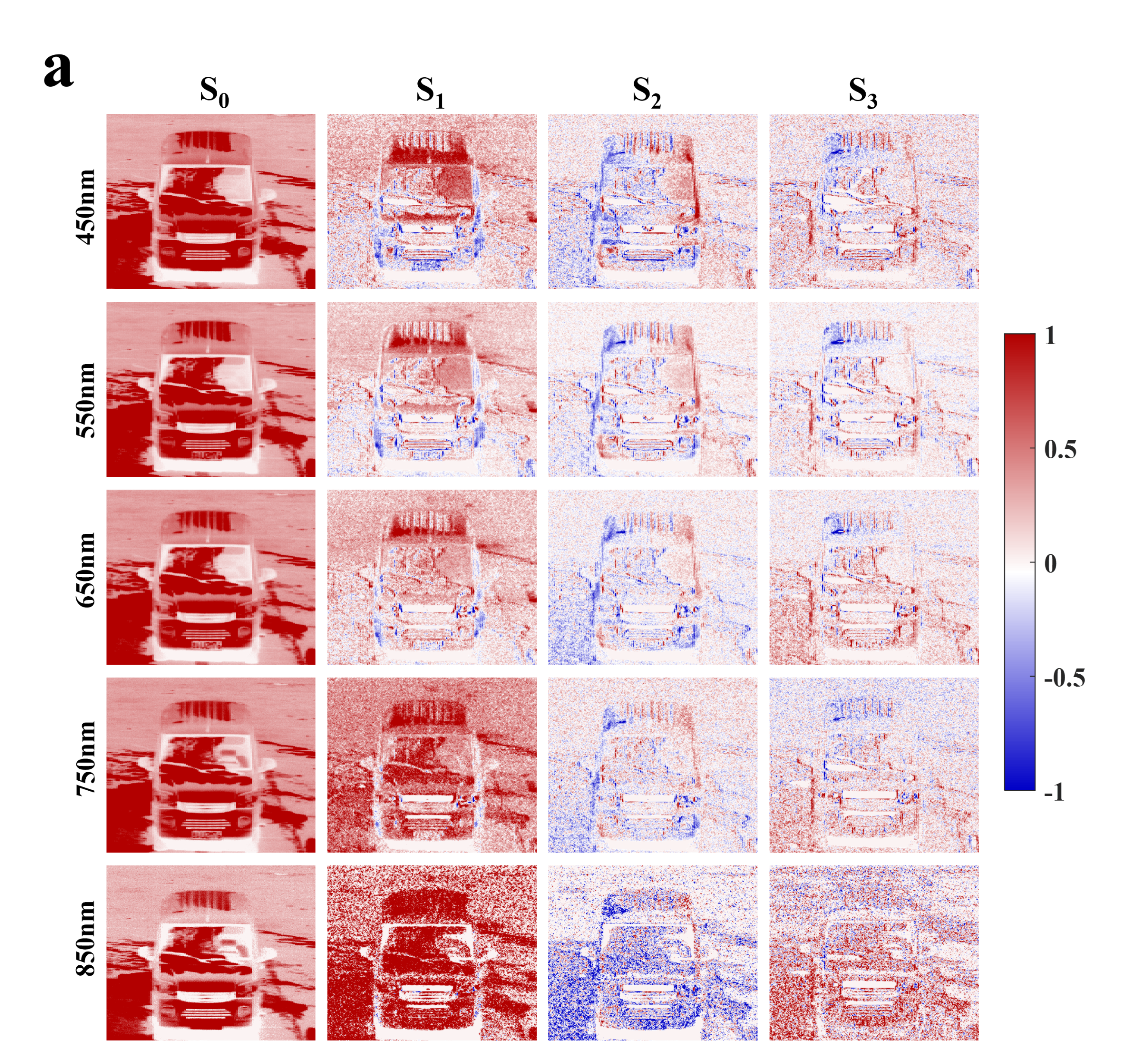


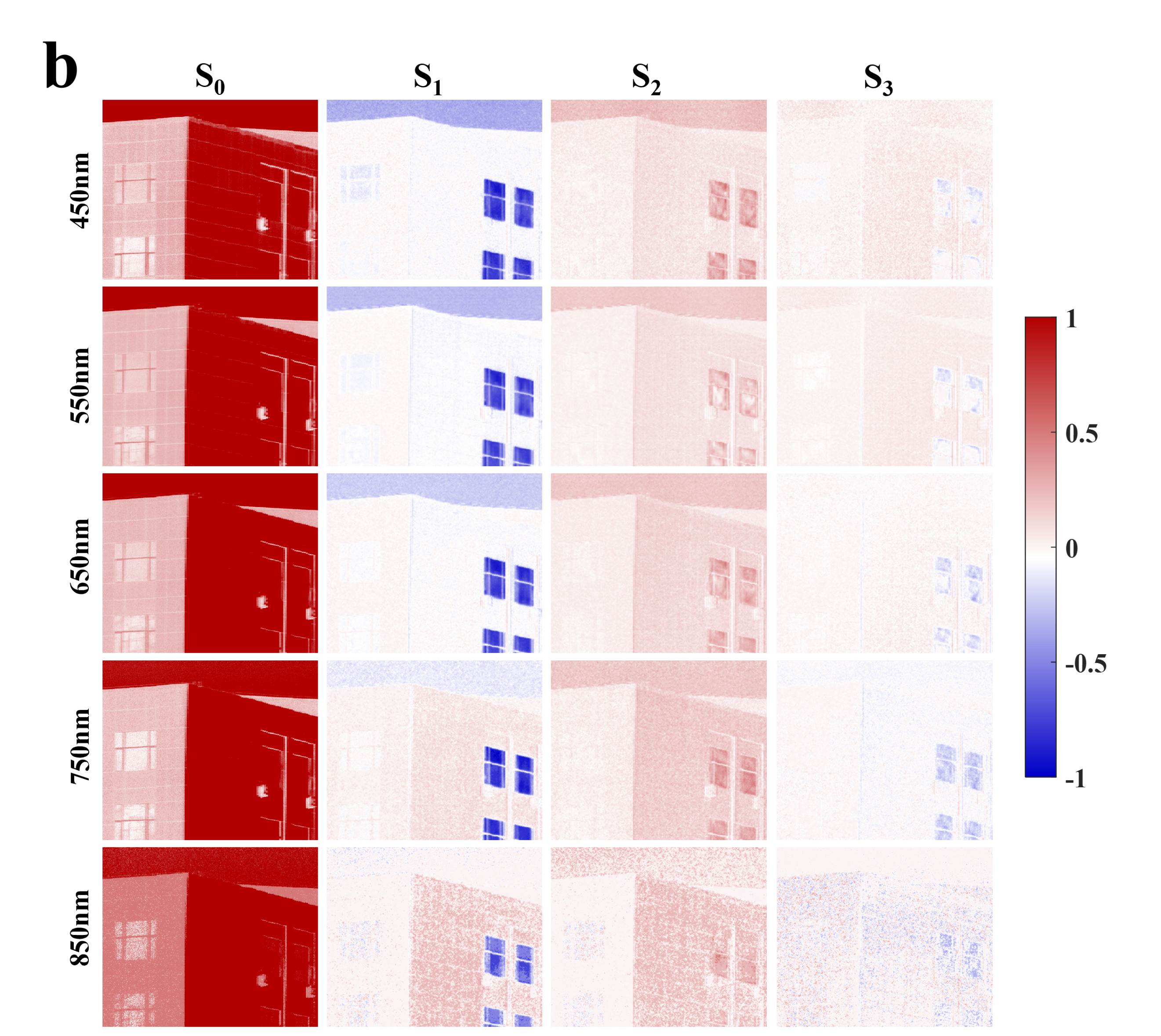


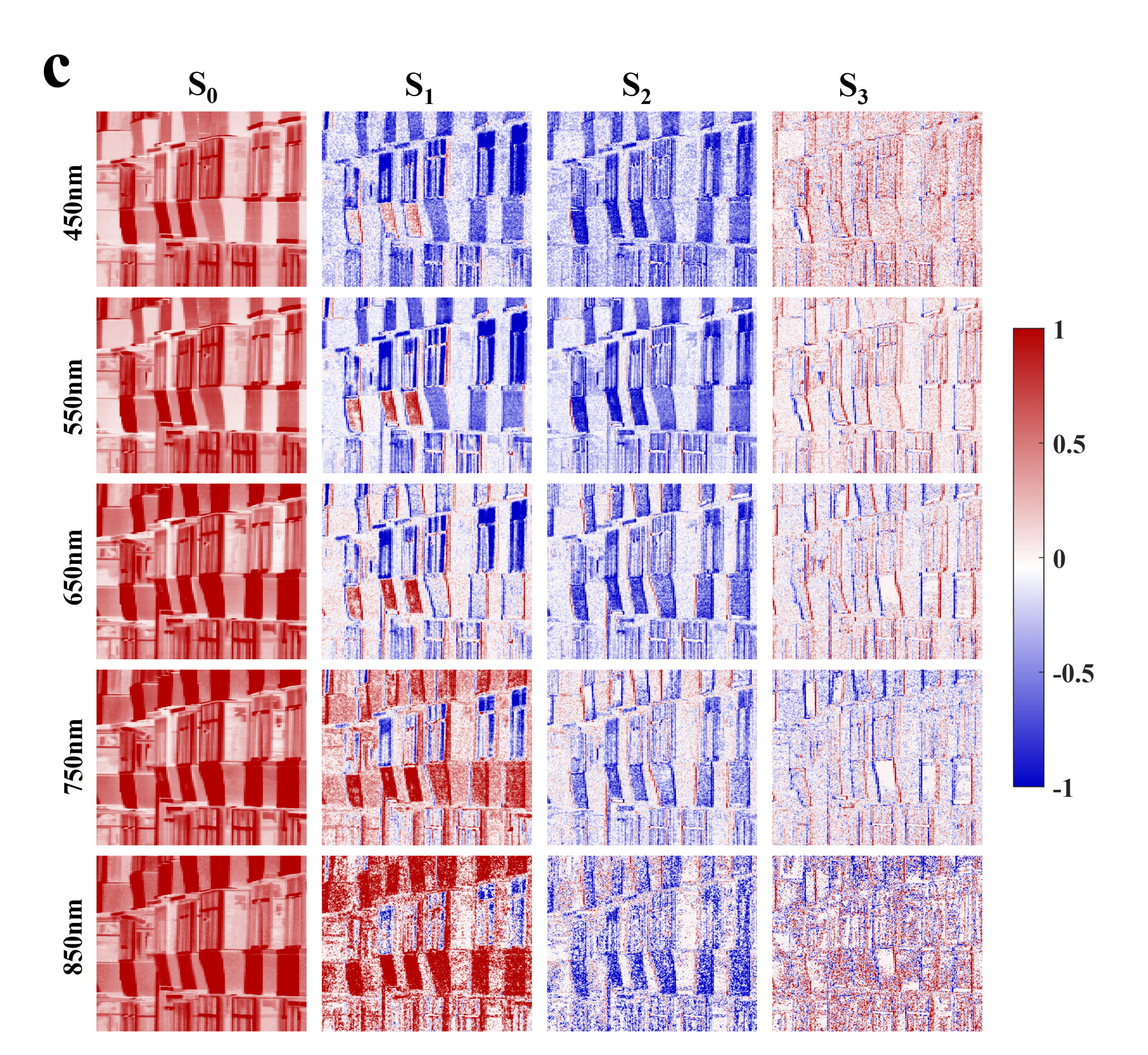


Figure S25. Ground-truth images for different target scenes.

**Supplementary Note 20: Ground-truth polarization-resolved spectral images for different target scenes.**


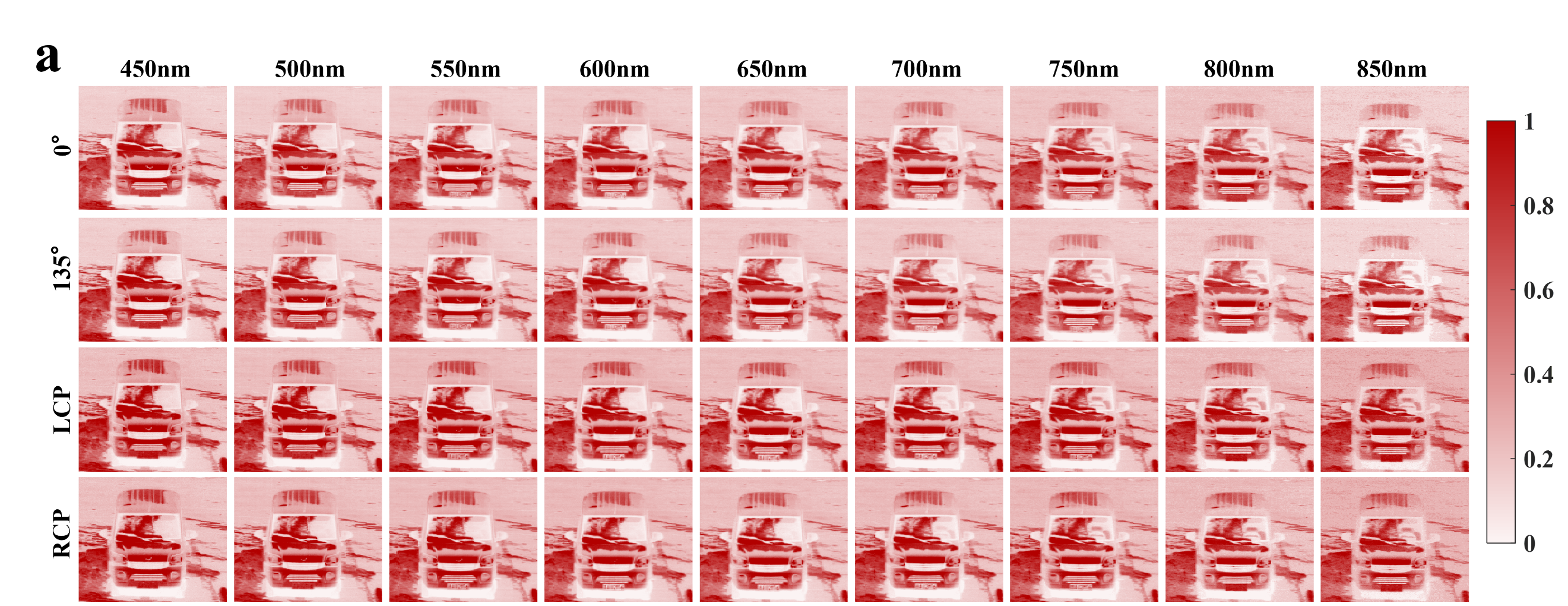

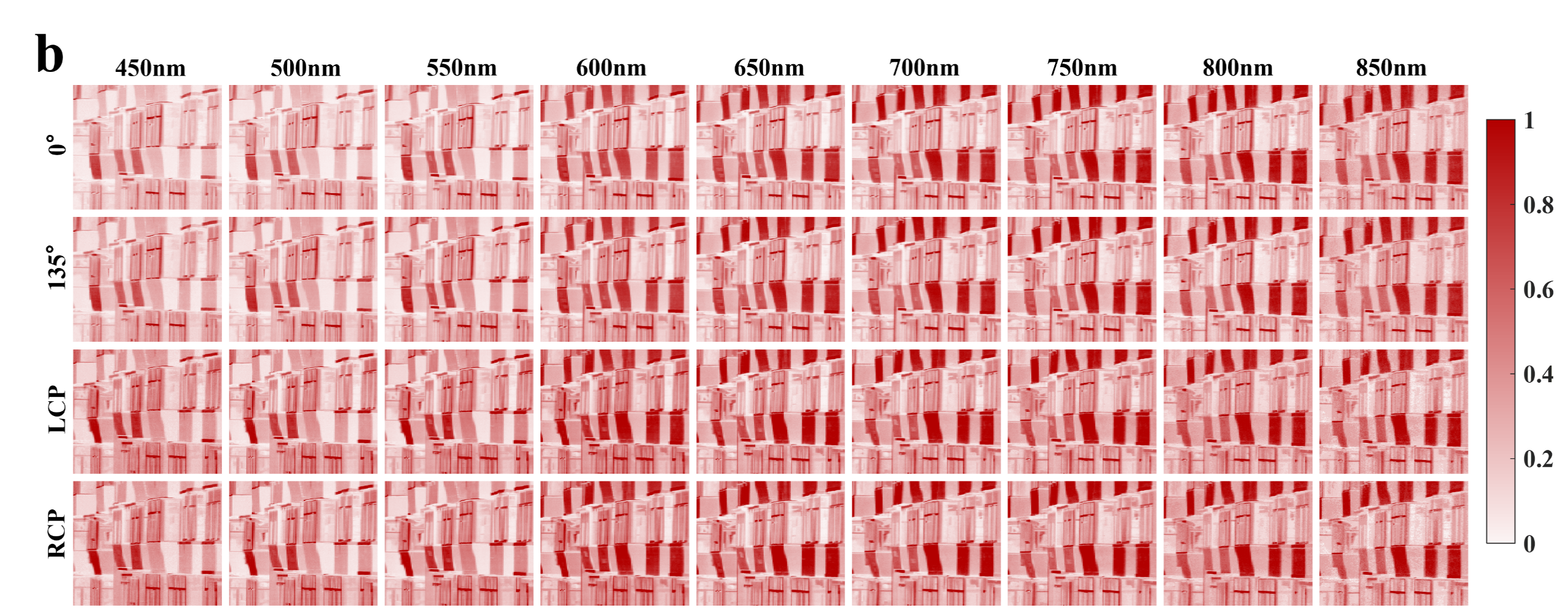


Figure S26. Ground-truth polarization-resolved spectral images.

**Supplementary Note 21: Network architecture for polarization-resolved spectral image reconstruction.**

Figure S27 illustrates the architecture of the U-Net-based reconstruction network used for polarization-resolved spectral image reconstruction. Detailed descriptions of the network design are provided in Supplementary Note 18. The reconstructed spectral images cover a wavelength range from 450 nm to 850 nm, uniformly sampled with 41 spectral channels.


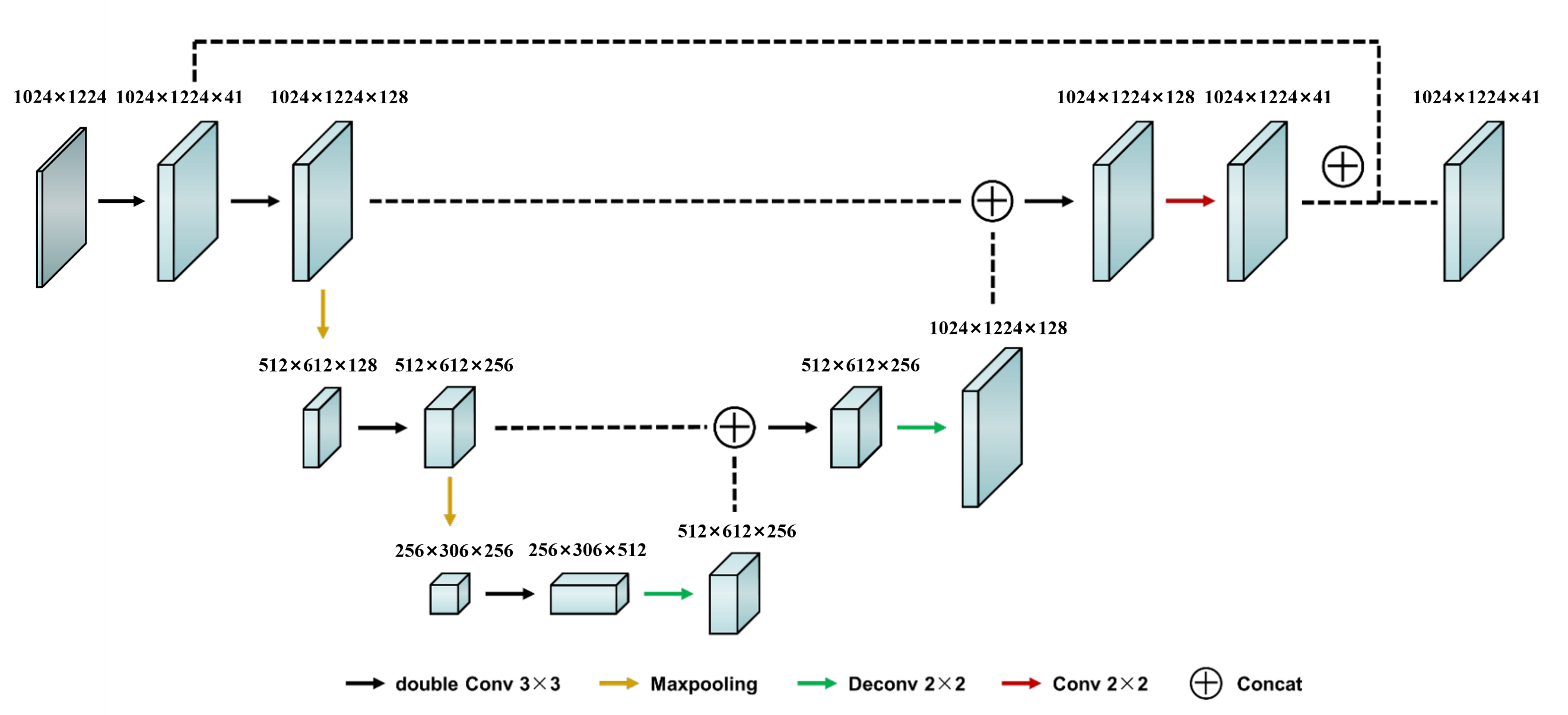


Figure S27. Architecture of the U-Net network for polarization-resolved spectral image reconstruction.

**Reference**

1. V. Karagodsky, C. J. Chang-Hasnain, *Optics Express* **2012**, 20, 10888.
2. M. Saldutti, P. Bardella, J. Mork, M. Gioannini, *Ieee Journal of Selected Topics in Quantum Electronics* **2019**, 25, 4500511.
3. L. Li, H. Zhao, C. Liu, L. Li, T. J. Cui, *Elight* **2022**, 2, 7.
4. A. I. Kuznetsov, A. E. Miroshnichenko, M. L. Brongersma, Y. S. Kivshar, B. Luk'yanchuk, *Science* **2016**, 354, aag2472.
5. Y. Kiarashinejad, S. Abdollahramezani, A. Adibi, *Npj Computational Materials* **2020**, 6, 12.
6. A. Y. Ng, *21st International Conference on Machine Learning* **2004**, 615-622.
7. H. Shi, C. Gong, Q. Wang, J. Liu, J. Wang, Y. Li, H. Sun, C. Wang, Y. Ma, X. Kang, H. Jiang, *Optics and Laser Technology* **2025**, 191, 113409.
8. J. Zuo, J. Bai, S. Choi, A. Basiri, X. Chen, C. Wang, Y. Yao, *Light Sci Appl* **2023**, 12, 218.
9. R. Hruska, J. Mitchell, M. Anderson, N. F. Glenn, *Remote Sensing* **2012**, 4, 2736-2752.
10. O. Ronneberger, P. Fischer, T. Brox, *CORR*, **2015**.
